# Supplementary material for: The association of COVID-19 lockdowns with adverse birth and pregnancy outcomes in 28 high-income countries: a systematic review and meta-analysis
Source: Nat Hum Behav. 2025 Apr 30;9(7):1420–30. doi: 10.1038/s41562-025-02139-z (PMC12283399; doi:10.1038/s41562-025-02139-z)
Supplement: Supplementary file 1 — Supplementary Material 1–3 (materials related to the methodology). Supplementary Material 1. Outcome definitions. Supplementary Material 2. Search term. Supplementary Material 3. Details regarding the deprivation indicator variable. Supplementary Material 4. Table of included studies. Supplementary Material 5. Table of included studies (time-adjusted analysis). Supplementary Material 6. PRISMA flowchart. Supplementary Material 7–9 (supplementary results). Results and analyses of outcomes’ subclassifications and subgroup analysis by region. Supplementary Material 8. Investigation of publication bias. Supplementary Material 9. Investigation of power and minimum detectable effects. Supplementary Material 10 and 11 (supplementary methods information). List of excluded studies from meta-analysis and full text screening. Supplementary Material 12. PRISMA checklist (information relevant to discussion). Supplementary Material 13. List of supplementary figures titles and captions. Supplementary Figs. 1–15. [file 41562_2025_2139_MOESM1_ESM.pdf]

nature human behaviour

Supplementary information https://doi.org/10.1038/s41562-025-02139-z

The association of COVID-19 lockdowns with adverse birth and pregnancy outcomes in 28 high-income countries: a systematic review and meta-analysis

In the format provided by the authors and unedited

Supplementary Material

Table of Contents

Supplementary Material 1: Outcome Definitions. 2

Supplementary Material 2: Search term (PubMed) 6

Supplementary Material 3: Deprivation Indicator Variable 7

Supplementary Material 4. Table of studies included in meta-analysis with key characteristics & risk of bias assessment. 13

Supplementary Material 5: Tables of studies included in time-adjusted meta-analysis with key characteristics & risk of bias assessment. 43

Supplementary Material 6: PRISMA Flowchart of study selection 47

Supplementary Material 7: meta-analyses of outcomes by sub-groups and subclassifications 48

Perinatal/Neonatal Outcomes 48

Preterm Birth: 48

Low birthweight: 48

Neonatal admissions: 48

Maternal Health Outcomes 49

Caesarean Section: 49

Maternal Readmission 49

Maternal Mental Health Outcomes 49

Supplementary Material 8: funnel plots and Egger's test results 50

Supplementary Material 9: Power calculations and minimum detectable effects. 50

Supplementary Material 10: studies excluded from meta-analysis with brief reasons. 52

Supplementary Material 11: list of studies excluded at full text screening with brief reasons 59

Supplementary Material 12: PRISMA 2020 Checklists 73

Supplementary Material 13. List of Supplementary Figures & Captions 77

Supplementary Material 1: Outcome Definitions.

Outcomes (Neonatal/Perinatal)	Definition
Preterm birth (PTB)<br><br>Sub-classifications explored in supplementary analysis (Supplementary material 1) include:<br>- extreme<br>- very<br>- moderate to late<br>- iatrogenic<br>- spontaneous	Birth/delivery at less than 37 weeks gestational age.<br><br>Sub-classification definitions:<br><br>Extreme PTB: birth <28 weeks gestational age<br><br>Very PTB: birth between >28-<32 weeks gestational age<br><br>Moderate - late PTB: birth between >32-<37 weeks gestational age<br><br>Iatrogenic: medically indicated or initiated PTB<br><br>Spontaneous: spontaneous onset of preterm labour and birth (vaginal or by caesarean section delivery)
Stillbirth	Foetal death at either antepartum, intrapartum, or indeterminate (more than 20- & 22-weeks gestational age)
Low birth weight (LBW)<br><br>Sub-classifications explored in Supplementary Analysis (Supplementary material 1):<br><br>- Small for gestational age (SGA)<br>- Low birth weight<br>- Very low birth weight<br>- Extremely low birth weight<br><br>Small for gestational age	<2500 grams birth weight.<br><br>Sub-classification definitions:<br><br>SGA: Birth weight less than the 10th centile by WHO-UK90 growth reference.<br><br>Low birth weight: <2500 g<br><br>Very low birth weight: <1500 g<br><br>Extremely low birth weight: <1000 g<br><br>Small for gestational age: <10th centile, about <2362 grams at 37 weeks
Neonatal mortality	Death of a neonate (newborn) at either during the birth episode or in a subsequent hospital readmission, in the first 30 days of life.

Neonatal admissions	All live born neonatal admissions, or live born infants admitted to the neonatal intensive care unit.
Hypoxic Ischemic Encephalopathy (HIE).	Neonatal brain injury related to hypoxic-ischemic event in the intrapartum.
Prolonged stay in hospital	Infant prolonged stay of more than 48 hours or 3 days in hospital

**Outcomes (Maternal)**	**Definition**
Caesarean Section<br><br>Sub-classifications explored in Supplementary Analysis (Supplementary material 1):<br><br>- planned<br>- emergency	Surgical delivery of a newborn through an incision in the mother's abdomen.<br><br>Sub-classification definitions:<br><br>Planned: planned due to medical or non-medical reasons.<br><br>Emergency: unexpected or unplanned emergency procedure due to maternal or foetal compromise.
Readmission to hospital	Maternal readmission to hospital when discharged within 84 days post-delivery.
Prolonged stay in hospital	Maternal prolonged stay of more than 2 days in hospital post birth.
Hysterectomy	Planned or unplanned surgical removal of the uterus during or after birth.
Obstetric and Anal Sphincter Tears (OASI)	3rd and 4th degree tear, perineal tear, high grade perineal tears in the vaginal wall and sphincter region, which occur during birth.
Sepsis	Infection and systematic manifestations of infection in puerperium.
**Maternal mental health outcomes**	
Maternal depression	Women with clinically relevant depression scores during pregnancy. The following questionnaires and scales with relevant cut off scores were used among studies:<br>- Kessler Pyschological Distress Scale (score >=30)<br>- CESD (Centre for Epidemiological Studies Depression scale) (score > 15)<br>- Edinburgh Postnatal depression score (EPDS) (cut off used for 2nd trimester: score>10, 3rd trimester: score>13. Any trimester score>15)

	- Depression Anxiety Stress Scales (DASS) (severe depression classification score: 21-27)
Post-partum depression	Individuals with clinically relevant depression scores in postpartum. The following questionnaires and cut off scores were used to assess the outcome:<br>- Scales (cut off scores for clinically relevant symptoms):<br>- Specific questions on postpartum emotions (yes to one question = clinically relevant depression symptoms)<br>- EPDS (Edinburgh Postnatal Depression Scale) 10 item (cut off score for depression symptoms used in studies: score >9, >10, >12, >13, >14).<br>- CESD (Centre for Epidemiological Studies Depression scale) (cut off used score > 15)
Maternal anxiety	Individuals with clinically relevant scores in pregnancy, measured with the following questionnaires and scales, and the cut off scores for clinically relevant symptoms are reported alongside in brackets:<br>- ASS (Anxiety Symptoms Scale)<br>- Generalized Anxiety Scale (GAD) 2 item (score > 3) and 20 item (GAD-7) (score >58.7)<br>- STAI (State Trait Anxiety Inventory)<br>- Kessler Psychological distress scale 10 item (score >= 30)<br>- Depression Anxiety Stress Scales (DASS) (severe Anxiety classification score 15-19)
Postpartum anxiety	Individuals with clinically relevant scores postpartum, measured with the following questionnaires and scales, and the cut off scores for clinically relevant symptoms are reported alongside in brackets:<br>- ASS (Anxiety Symptoms Scale)

	- Generalized Anxiety Scale (GAD) 2 item (score > 3) and 20 item (GAD-7) (score >58.7)<br>- STAI (State Trait Anxiety Inventory)<br>- Kessler Psychological distress scale 10 item (score >= 30)<br>- Depression Anxiety Stress Scales (DASS) (severe Anxiety classification score 15-19)

Supplementary Material 2: Search term (PubMed)

(((((((((((((lockdown) OR (lock down)) OR (lock-down)) OR (home confinement)) OR (work-from-home)) OR (home office)) OR (restrictions)) OR (non-essential services closure)) OR (home office)) AND (covid-19))) OR (coronavirus)) OR (SARS-Cov-2)) AND (((((((((((((((((((((((neonatal admissions) OR (premature birth)) OR (preterm birth)) OR (stillbirth)) OR (prolonged maternal stay)) OR (prolonged neonatal stay)) OR (maternal readmission)) OR (Caesarean section)) OR (emergency caesarean section)) OR (Obstetric anal sphincter injuries)) OR (hysterectomy)) OR (sepsis labor)) OR (sepsis delivery)) OR (sepsis puerperium)) OR (prolonged maternal stay hospital)) OR (maternal mental health)) OR (postnatal depression)) OR (maternal anxiety)) OR (maternal depression)) OR (small gestational age)) OR (Hypoxic Ischemic Encephalopathy)) OR (neonatal death))) OR (pregnancy complications) OR (birth complications))

Supplementary Material 3: Deprivation Indicator Variable

Some studies reported data stratified by patient's area deprivation index. An area deprivation index uses a variety of socio-economic factors to estimate the level of socioeconomic deprivation in an area where a person lives, the area is then allocated to one of five deprivation quintiles ranging from low to high. However, some studies did not report data according to a deprivation index, but instead reported data based on individual socioeconomic factors, such as maternal education level, or an area's average income. Some of these variables could be used as a proxy for deprivation.

Studies which presented data stratified according to deprivation index or a relevant proxy variable for deprivation were identified. Individual studies were not assigned to deprivation levels, their stratified data was. For each of these studies presenting relevant data, the stratified data was extracted and allocated to low, medium, and high deprivation sub-groups. The process of harmonising data to deprivation levels is outlined in the table below.

The table presents each study included in the meta-analysis, which presented data stratified according to a deprivation index or a proxy variable. Each row is allocated to a single study which presented data stratified according to deprivation or a relevant proxy variable. In the first columns of the table the core details of the study are identified. In the following columns we identify the variable of deprivation which the study used (either a deprivation index or a proxy variable), the source of the variable, if a deprivation index was used then we outline what socioeconomic factors are included in the index. The subsequent columns outline how the groups of the deprivation variable are defined by the data source, followed by how the individual study presented data (some studies aggregated groups or quintiles to indicate higher / lower deprivation). In the final column, we indicate what level of deprivation (low, medium, high) we allocated to each group of the variable, in each study.

First author last name and year of publication	Variable (as defined in publication)	Data source of variable	Definition of variable	If an index, sociodemographic factors included in index caculation	Group definitions of variable according to the data source	Group as defined in publication, by which data was presented in the paper	Level of deprivation group allocated to in our analysis
**Harvey 2021**	Maternal education	Tennesee birth records	Education level of mother	N/a	less than high school	less than high school	high deprivation
					high school/general education diploma	high school/general education diploma	medium deprivation
					some college/associate degree	some college/associate degree	low deprivation
					college degree	college degree	
**Shah 2021**	Neighbourhood income quintile	Statistics canada postal code conversion file	Neighbourhood area level income	N/a	quintile 1 (poorest)	quintile 1 (poorest)	high deprivation
					quintile 2	quintile 2	
					quintile 3	quintile 3	medium deprivation
					quintile 4	quintile 4	

					quintile 5 (richest)	quintile 5 (richest)	low deprivation
**Aboulatta 2023**	Mean household income	Manitoba Population Research Data Repository	Census data for income quintiles based on ranges of mean household income, and grouped into five categories with each quintile assigned to approximately 20% of the population (quintile 1 (mean income=$C17 910) to quintile 5 (mean income=$46 230)).	N/a	lower income (individuals in the lowest and second lowest median neighbourhood income quintile)	lower income (individuals in the lowest and second lowest median neighbourhood income quintile)	high deprivation

					higher income (individuals residing in the neighbourhoods with the three highest median neighbourhood income quintiles)	higher income (individuals residing in the neighbourhoods with the three highest median neighbourhood income quintiles)	low deprivation
**Been 2020**	Neighbourhood socioeconomic status	Netherlands Institute for Social Research	Neighbourhood area level deprivation	Mean household income, proportion of population with low income, proportion of population with low education level, proportion of population without paid work.	quintile 1 (poorest)	low (<p20)	high deprivation
					quintile 2	medium (p20-80)	medium deprivation
					quintile 3		
					quintile 4	high (>=p80)	low deprivation
					quintile 5 (richest)		
**Fisher 2022**	Neightbourhood deprivation index	United States Census Bureau. American community survey 5-year data (2009-2019). 2020. Available at:	Neighbourhood area level deprivation	Dependency, educational attainment, unemployment, poverty, per capita income, and crowded housing	first	lower three quartiles	low deprivation
					second		
					third		
					fourth	highest quartile	high deprivation

		https://www.census.gov/data/developers/data-sets/acs-5year.html.					
**Gurol-Urganci 2022**	Neighbourhood deprivation index	2019 Index of Multiple Deprivation, Available at: https://www.gov.uk/government/statistics/english-indices-of-deprivation-2019)	2019 Area Index of Multiple Deprivation	Income, education, employment, crime, and living environment in an individual's area of residence	quintile 1 (least deprived)	less deprived	low deprivation
					quintile 2		
					quintile 3		
					quintile 4	more deprived	high deprivation
					quintile 5 (most deprived)		
**Lemon 2021**	Neighbourhood deprivation index	Department of Medicine, School of Medicine and Public Health, University of Wisconsin. Neighborhood Atlas. 2015. Available at: https://www.neighborhoodatlas.medicine.wisc.edu/.	Area deprivation index	Income, education, employment, and housing quality	ADI tertile 1 (1-52)	ADI tertile 1 (1-52)	low deprivation
					ADI tertile 2 (53-75)	ADI tertile 2 (53-75)	medium deprivation
					ADI tertile 3 (75 - 100)	ADI tertile 3 (75 - 100)	high deprivation

Supplementary Material 4. Table of studies included in meta-analysis with key characteristics & risk of bias assessment.

	**Study Design**	**Country**	**Region** (site)	**Outcomes included** (either in overall or as sub-classifications in supplement)	**Stratified data** (by ethnicity group and/or deprivation level)	**Pre-COVID pandemic** (dates)	**Lockdown 1** (dates)	**Lockdown 2** (dates)	**Post-Lockdown** (dates)	**Exposed (lockdown) Sample Size**	**Un-Exposed (pre-lockdown) Sample Size**	**Newcastle Ottawa Overall Bias Assessment** (Poor, Fair, Good)
Aboulatta 2023	cohort study	Canada	Manitoba	Preterm birth	Deprivation	1 October 2016 - 29 February 2020	1 March 2020 - 31 March 2021	..	..	8492	28481	Good
Adeluwoye 2021	cross-sectional study	UK	South London (Lewisham and St.Thomas)	Iatrogenic preterm birth	..	April 2019	April 2020	..	..	567	560	Fair
Afonina 2021	cohort study	Italy	Milan (Sao Paolo Hospital Medical School)	SGA*	..	1 April - 31 December 2019	1 April - 31 December 2020	..	..	858	968	Fair

Allegri 2023	cross-sectional study	Italy	Milan (Niguarda Ca'Granda Hospital)	Screening for depression in pregnancy & screening for anxiety in pregnancy	..	September 2019 - February 2020	..	November 2020 - April 2021	..	68	88	Good
Alshaikh 2021	cohort study	Canada	Calgary	Low birthweight (extreme, very & SGA)	..	16 March - June 15, 2015 - 2019	16 March – 15 June 2020	..	..	4334	24018	Good
Amadori 2021	cohort study	Italy	Novara (University Hospital Maggiore della Carita)	Low birthweight (SGA), OASI*, planned & emergency caesarean section	..	April - May 2017	April - May 2020	..	..	256	293	Good
Ameh 2021	cohort study	United Kingdom	England, Bolton (Royal Bolton Hospital)	Neonatal mortality & neonatal admissions	..	1 November 2019 - 31 January 2020	1 March - 31 May 2020	..	..	1461	1553	Good
Arnaez 2021	prevalence proportion study	Spain	Castilla y Leon	Preterm birth (extreme, very & moderate to late), stillbirth & low	..	15 March - 3 May, 2015 - 2019	15 March - 3 May 2020	..	4 May - 21 June 2020	1499	65201	Good

				birthweight (extreme & very)								
Athar 2022	cohort study	Qatar	AlWakra	Preterm birth, neonatal admissions & SGA	..	February - July 2019	February - July 2020	..	..	4336	3154	Fair
Athiraman 2022	cohort study	UK	North East England (Sunderland Royal Hostpial, James Cook University Hospitals, Royal Victoria Infirmary)	Neonatal admissions, extreme, very & moderate to late preterm birth	..	1 March - 30 June, 2018 - 2019	1 March - 30 June 2020	..	..	4738	9538	Good
Ayyash 2022	cohort study	USA	Michigan	Preterm birth	Ethnicity	March - December 2019	March - December 2020	..	..	73996	82417	Good
Bajaj 2022	cohort study	USA	Michigan	Neonatal admissions	..	March – November, 2017, 2018, and 2019.	March - November 2020	..	..	77983	250896	Good
Bajaj 2022	cohort study	USA	Flo health app	Screening for	..	January 2018 -	March 2020 -May 2020	..	..	118622	159478	Fair

				postpartum depression		February 2020						
Balsa 2022	cohort study	Uruguay	all	Preterm birth (very and moderate to late), stillbirth, low birthweight (very) & caesarean section	..	5 December 2018 - 4 December 2020	14 March – 4 December 2020	..	..	25181	45720	Good
Been 2020	cohort study	Netherla nds	all	Preterm birth	Deprivation	9 October 2010 - 9 March 2020	9 March – 16 July 2020	..	..	26621	505202	Good
Benyamini Raischer 2023	cohort study	Israel	Afula (University hospital)	Low birthweight & caesarean section	..	April - September 2019	April - September 2020	..	..	2442	2323	Good
Berghella 2020	cohort study	USA	Philadelphi a (Thomas Jefferson University)	Iatrogenic, spontaneous & extreme preterm birth	..	1 March - 31 July 2019	1 March – 31 July 31 2020	..	..	1197	911	Good
Berthelot 2020	cohort study	Canada	Quebec	Screening for depression in pregnancy & screening	..	1 April 2018 - 1 March 2020	2 April - 13 April 2020	..	..	1258	496	Fair

				for anxiety in pregnancy								
Boehler-Tatman 2022	cohort study	USA	Providence (Rhode Island Women and Infants Hospital)	Planned caesarean section	..	April 2019	April 2020	..	..	254	235	Good
Boekhorst 2021	cohort study	Netherlands	Brabant	Screening for postpartum depression	..	7 January 2019 - 1 March 2020	1 March 2020 - 14 May 2020	..	..	268	401	Poor
Cate 2022	cohort study	USA	Durham (Duke University Medical Centre)	Preterm birth (extreme)	Ethnicity	March - June 2014 - 2019	March - June 2020	March - June 2021	..	2646	15574	Good
Chen 2022	cohort study	USA	all	Stillbirth	..	April - June 2019	April - June 2020	..	..	77193	88240	Good
Cheung 2021	cohort study	Canada	Alberta and Edmonton	Very & moderate to late preterm birth	..	1 March - 30 April 2019	1 March - 30 April 2020	..	..	5546	6007	Good
Chrzan-Detkos 2021	cohort study	Poland	Northern	Screening for postpartum depression	..	1 October - 10 November 2019	20 February - 30 March 2020	..	..	78	61	Fair
Clifton 2022	cohort study	Australia	Brisbane (Mater	Screening for	..	August 2018 -	March - August 2020	..	September -	156	243	Fair

			Mother's Hospital)	depression in pregnancy, screening for postpartum depression & screening for anxiety in pregnancy		February 2020			December 2020			
Dagelic 2023	cohort study	Croatia	Split (University hospital)	Stillbirth & neonatal mortality	..	1 March 2019 - 29 February 2020	1 March 2020 - 1 March 2021	..	..	4153	4293	Good
David 2023	cohort study	Germany	Berlin	Spontaneous, extreme & very preterm birth	..	January 2017 - December 2019	January - December 2020	..	..	38782	199586	Good
De Chiara 2022	cohort study	Italy	Rome (San Pietro Fatenefratelli Hopsital)	Screening for depression in pregnancy & screening for anxiety in pregnancy	..	July - September 2019	April - July 2020	..	..	72	68	Fair
Debolt 2022	cohort study	USA	New York City (Mount Sinai Hospital)	Neonatal admissions, hysterectomy, prolonged maternal stay &	..	March - May 2019	March - May 2020	..	..	1078	1910	Fair

				maternal readmission								
Deli 2021	cohort study	Hungary	Debrecen	Cesarean section	..	January 2020 - February 2020	March - May 2020	November 2020 - January 2021	..	748	517	Good
Delius 2023	cohort study	Germany	Bavaria (Ludwig Maximillian University)	Cesarean section	..	March 22 - May 5, 2018 - 2019	22 March - 5 May 2020	..	..	333	704	Good
Dol 2021	cohort study	Canada	Eastern Maritime Provinces	Screening for postpartum depression & screening for postpartum anxiety	..	1 October 2019 - 1 January 2020	1 August - 31 October 2020	..	..	331	561	Good
Einarsdottir 2021	cohort study	Iceland	all	Preterm birth (iatrogenic, spontaneous & moderate to late), stillbirth & cesarean section (planned & emergency)	..	2016 - 2020	13 March - 25 May 2020	5 October - 31 December 2020	..	1057	3954	Good

Esposito 2023	cohort study	Italy	Lombardy	Low birthweight & cesarean section (planned & emergency)	..	16 March 2018 - 5 April 2019	16 March - 2 June 2020	3 November 2020 - 5 April 2021	..	13476	14952	Good
Facco 2021	cohort study	USA	Pennsylvania (Magee womens hospital, University of pittsburg medical school)	Maternal readmission	..	1 April - 1 July 2019	1 April - 1 July 2020	..	..	2417	2368	Good
Feldman 2021	cohort study	USA	New York City (Mount Sinai West)	Extreme preterm birth	..	March - May 2019	March - May 2020	..	..	1135	1306	Good
Fischer 2022	cohort study	USA	Chicago (Northwestern Memorial Hospitals Prentice Womens Hospital)	Preterm birth (iatrogenic & spontaneous), stillbirth	Deprivation	1 April - 30 November 2019	1 April - 30 November 2020	..	..	8298	8246	Good
Fresson 2022	cohort study	France	all	Preterm birth (extreme &	..	1 January 2016 -	17 March – 10 May 2020	..	..	96880	3108192	Good

				very), stillbirth & cesarean section		March 17th 2020						
Gallo 2022	cohort study	Australia	Queenslan d (Mater Mother's health care records)	Stillbirth	..	16 March - 1 May, 2013 - 2019	16 March – 17 April 2020	30 March 1 May 2020	26 April - 9 May 2020	1237	8796	Good
Garabedian 2021	cohort study	France	Lille, Nantes, Toulouse, Strasbourg , Poissy, Necker	Preterm birth & Low birthweight (very)	..	22 January 2020 - 17 March 2020	17 March - 11 May 2020	..	11 May - 4 July 2020	3809	4071	Good
Gemmill 2021	cohort study	USA	national center of health statistics	Preterm birth, low birthweight (very) & cesarean section	..	January 2015 - December 2019	March - July 2020	October December 2020	..	1516206	19906408	Good
Green 2022	cohort study	USA	Southern Carolina (Medical university of south Carolina, Greenville)	Screening for postpartum depression	..	1 April - 31 December 2019	1 April - 31 December 2020	..	..	677	420	Fair
Gulerson 2022	cohort study	USA	New York (7 (all non-	Maternal readmission	..	1 April - 15 June 2019	1 April - 15 June 2020	..	..	4038	4168	Good

			Mount Sinai))									
Gurol-Urganci 2022	cohort study	UK	England	Preterm birth, stillbirth, SGA, planned cesarean section, emergency cesarean section, prolonged maternal stay & maternal readmission	Ethnicity * & Deprivation	23 March 2019 - 22 February 2020	23 March - June 2021	22 september 22 February 20202021	..	126093	496293	Good
Gustafsson 2021	cohort study	USA	Oregon (Oregon health & Science university Portland)	Screening for depression in pregnancy & screening for postpartum depression	..	December 2018 - 23 March 2020	April, 2020 - 2021	..	..	146	75	Fair
Handley 2022	cohort study	USA	Epics systems cosmos research platform	SGA	..	March 2017 - February 2020	March 2020 - February 2021	..	..	252155	742113	Fair

Handley 2022	cohort study	USA	Philadelphia (GeoBirth, 2 penn medicine hospitals)	Preterm birth (iatrogenic, spontaneous, extreme & very)	Ethnicity	10 March - 31 December, 2018 - 2019	10 March - 31 December 2020	..	..	3432	7123	Good
Harvey 2021	cohort study	USA	Tennessee	Preterm birth	Ethnicity & Deprivation	22 March 2015 - 30 April 2019	22 March - 30 April 2020	..	..	7553	41577	Good
Herzberger 2021	cohort study	Israel	Saba (Meir Medical centre)	Neonatal admissions, cesarean section & OASI	..	1 March - 23 July 2019	1 March - 23 July 2020	..	..	2668	2701	Fair
Hiiragi 2021	cohort study	Japan	Yokohama (Yokohama City University Medical Centre)	Preterm birth, neonatal admissions & screening for postpartum depression	..	March - June 2019	March - June 2020	..	..	279	339	Fair
Hirsch 2021	cohort study	Israel	Jerusalem (Shaare Zedek Medical centre)	Maternal readmission, planned & emergency cesarean section	..	10 March - 12 May 2019	10 March - 12 May 2020	..	..	2343	2665	Fair
Hui 2021	cohort study	Hong Kong	Hong Kong (Queen	Screening for	..	1 January 2019 - 4	5 January 2020 - 30 April 2020	..	..	802	2970	Good

			Mary Hospital)	postpartum depression		January 2020						
Hui 2022	cohort study	Australia	Melbourne	Neonatal admissions & preterm birth (iatrogenic, spontaneous , extreme & very)	..	6 November 2017 - 3 June 2019	23 March 2020 - 14 March 2021		..	24732	49892	Fair
Huseynova 2021	cross-section al study	Saudi Arabia	Riyadh (King Saud Medical City)	Preterm birth (extreme, very & moderate to late)	..	1 March - 30 June, 2017 - 2019	1 March - 30 June 2020		..	1763	5463	Fair
Hwang 2022	cohort study	USA	Colorado	Preterm birth	Ethnicity	April – December, 2015 - 2019	April - December 2020		..	45766	242636	Good
Hwang 2022	cohort study	South Korea	all	Preterm birth & low birthweight	..	January 2011 - December 2019	January - Demember 2020		..	255024	3481423	Good
Ibrahim 2025	cross-section al study	England	all	Preterm birth	Ethnicity	2018-2020	March – June 2020		..	426226	888543	Good
Janevic 2021	cohort study	USA	New York City (Mount	Preterm birth (very &	Ethnicity	28 March - 31 July 2019	28 March - 31 July 2020		..	3159	3327	Good

			Sinai Hospital)	moderate to late)								
Jasper 2022	cohort study	Australia	Queensland	Preterm birth (iatrogenic, spontaneous, extreme, very, & moderate to late), planned & emergency cesarean section	..	1 April - 31 May & 1 June - 31 July, 2018 - 2019	1 April - 31 May 2020	..	1 June - 31 July 2020	10701	22034	Good
Jeyamurugan 2023	cohort study	USA	New York (Brookdale Hospital Medical Center)	Neonatal admissions & extreme low birthweight	..	June - December 2019	June - December 2020	..	..	360	427	Good
Jones 2021	cross-sectional study	UK	Wales	Preterm birth (extreme, very, & moderate to late), stillbirth, low birthweight (extreme & very), neonatal mortality & cesarean	..	2016 - 2019	2020	..	..	29031	130326	Good

				section (planned & emergency)								
Justman 2020	cohort study	Israel	Haifa (Rambam health care campus)	Neonatal admissions & cesarean section (emergency)	..	March - April 2019	March - April 2020		..	610	742	Fair
Khalil 2020	cohort study	UK	England London (St Georges)	Neonatal admissions	..	1 October 2019 - 31 January 2020	1 February - 14 June 2020	..	..	1702	1677	Good
Kim 2021	cohort study	South Korea	Seoul (single hospital in city)	Preterm birth (extreme & very), stillbirth, very & extreme low birthweight, neonatal admissions & cesarean section	..	22 March 2011 - 31 October 2019	22 March - 31 October 2020	..	..	246	2765	Good
Kirchengast 2021	cohort study	Austria	Vienna (Danube hospital)	Low birthweight (extreme & very)	..	January - February 2020, 2005, & 2019.	March - July 2020	..	..	669	29084	Good

Klumper 2021	cross-sectional study	Netherlands	all	Preterm birth (extreme & very)	..	15 March - 15 May, 2015 -2018	15 March - 15 May 2020	..	..	26924	107326	Good
Kuipers 2022	cohort study	Belgium	Dutch speaking region	Screening for depression in pregnancy, Screening for postpartum depression, screening for anxiety in pregnancy & screening for postpartum anxiety	..	8 August 2019 - 3 February 2020	13 March 2020 - 17 February 2021	..	..	148	456	Fair
Lantigua-Martinez 2022	cohort study	USA	New York city (NYU Lagone & NYU Grossman school of medicine)	Screening for depression in pregnancy & screening for postpartum depression	..	February - July 2019	February - July 2020	..	..	310	938	Fair
Lau 2023	cohort study	Germany	all	Preterm birth, stillbirth & emergency	..	1 January 2017 - 21 March 2020	22 March - 31 December 2020	..	..	594728	1815118	Good

				cesarean section								
Leibovitch 2021	cohort study	Israel	all	Preterm birth (very & moderate to late)	..	1 January - 10 March 2020	11 March - 5 May 2020	..	6 May - 30 June 2020	25639	77903	Good
Lemon 2021	cohort study	USA	Pennsylvania (UPMC Magee Women's Hospital)	Preterm birth (iatrogenic, spontaneous & extreme)	Ethnicity & Deprivation	1 January 2018 - 31 January 2020	1 April - 27 October 2020	..	..	4957	16606	Good
Liu 2021	cohort study	Canada	excluding Quebec	Preterm birth & stillbirth	..	March – August, 2015 - 2019	March - August 2020	..	..	136445	717905	Good
Llorca 2021	cohort study	Spain	Santander (HUMV)	Low birthweight & cesarean section	..	1 January - 31 August 2018	23 March - 25 May 2020	..	..	620	969	Good
Lo 2022	cohort study	UK	England (Cambridge Hospital)	Screening for postpartum depression	..	23 - 29 November 2019	..	23 - 29 November 2020	..	99	92	Good
Loehr 2022	cohort study	USA	Virginia (University of Virginia)	Screening for postpartum depression	..	March - May 2019	March - May 2020	..	..	319	260	Good
Main 2021	cohort study	USA	California	Preterm birth (extreme,	Ethnicity	April – July, 2016 - 2019	April - July 2020	..	..	132853	580714	Good

				very, & moderate to late)								
Mak 2023	cohort study	Hong Kong	Hong Kong (Queen Mary Hospital)	Preterm birth (spontaneous, extreme, very, & moderate to late), stillbirth, low birthweight (extreme & very) & cesarean section	..	April 2018 - September 2019	April 2020 - September 2021	..	..	3738	5076	Good
Maki 2023	cross-sectional study	Japan	Miyazaki (Miyazaki Prefectural Nobeoka Hospital)	Preterm birth (extreme, very, & moderate to late), low birthweight (extremely & very), caesarean section & SGA	..	January 2017 - December 2019	1 March 2020 - 28 February 2021	..	..	1650	5762	Good
Marino-Narvaez 2021	cohort study	Spain	Granada (University	Screening for	..	1 September 2019 - 1	1 April - 1 July 2020	..	..	75	82	Fair

			of Granada)	postpartum depression		March 2020						
Maslin 2022	cohort study	UK	England South-West	Preterm birth (very & moderate to late) & neonatal admissions	..	2018 - 2019	2020	..	..	42926	91121	Fair
McDonnell 2020	cohort study	Ireland	Dublin (Coombe Women & infants university hospital)	Preterm birth	..	1 January - 31 July, 2018 - 2019	1 January - 31 July 2020	..	..	4397	9373	Fair
McKee 2023	cohort study	USA	Multiple states	Screening for depression in pregnancy	..	1 January 2016 - 11 March 2020	11 March 2020 - 31 May 2021	..	..	785	1570	Good
Meyer 2020	cohort study	Israel	Sheba (Sheba Medical Centre)	Caesarean section		February - March 2019	February - March 2020	..	..	1666	1654	Fair
Meyer 2021	cohort study	Israel	Sheba (Sheba Medical Centre)	Stillbirth, neonatal mortality, neonatal admissions & HIE, caesarean section	..	2011 - 2019	March - June 2020	..	..	2594	28686	Good

Mikus 2021	cross-sectional study	Croatia	Zagreb (University Hospital Centre Zagreb)	Preterm birth (extreme & very), stillbirth, & caesarean section	..	25 February - 31 December 2019	25 February -31 December 2020	..	..	2732	3277	Good
Molholm Hansen 2022	cohort study	Denmark	all	Preterm birth (extreme, very, & moderate to late), stillbirth & SGA	..	1 March 2016 - 28 February 2020	1 March 2020 - 28 February 2021	..	..	60323	244363	Good
Molina-Merino 2021	cohort study	Spain	Valencia	Preterm birth (extreme, very, & moderate to late)	..	14 March - 21 June, 2015 - 2019	14 March - 21 June 2020	..	..	9478	51484	Good
Mor 2021	cohort study	Israel	Zerifin (Shamir Medical Centre)	Stillbirth, neonatal admissions, caesarean section (planned & emergency)	..	21 February - 30 April, 2017 & 2019	21 February - 30 April 2020	..	..	1556	4564	Good
Muin 2021	cohort study	Austria	all	Preterm birth (extreme, very, &	..	March 2015 - December 2019	March - April 2020	March - December 2020	..	12517	66334	Good

				moderate to late) & stillbirth								
Niehaus 2023	prevalence proportion study	USA	Rhode Island (Women & Infants Hospital in Providence)	Neonatal admissions	..	February 2020	..	February 21	..	566	663	Good
Oakley 2022	cohort study	Norway	all	Preterm birth (iatrogenic, spontaneous & very)	..	Before 12 March, 2014 - 2019	From 12 March	..	..	31839	202678	Good
Ohashi 2023	cross-sectional study	Japan	Japan Medical Data Center database	Preterm birth & low birthweight	..	October – December, 2018 & 2019	..	October – December 2020	..	6021	11936	Good
Okawa 2022	cohort study	Japan	all (JACSIS study)	Preterm birth, low birthweight, neonatal admissions, planned & emergency cesarean section	..	October 2019 - March 2020	April - May 2020	..	June - October 2020	187	185	Fair

Panzer 2022	cohort study	USA (New york)	New York (New York Presbyterian Columbia University Irving Medical Centre/Morgan Stanley Children's Hospital and New York Presbyterian Allen Hospital)	Maternal readmission	..	22 March - 30 April 2019	22 March - 30 April 2020	..	..	643	715	Good
Pariente 2020	cohort study	Israel	Negev (Soroka University Medical Centre)	Screening for postpartum depression	..	Before 18 March 2020	18 March - 29 April 2020	..	..	223	123	Fair
Pereira 2022	cohort study	Portugal	Coimbra (Bissaya Barreto Maternity Hospital)	Screening for postpartum depression & screening for postpartum anxiety	..	2018 – 2019	..	January - March 2021	..	207	212	Fair

Philip 2020	cohort study	Ireland	Limerick (single largest hospital)	Low birthweight (extreme & very)	..	January - April, 2016 - 2020 & March - June, 2016 - 2019	March - June 2020	..	..	1381	5663	Fair
Quibel 2022	cohort study	France	Lille, Nantes, Toulouse, Strasbourg, Poissy, Necker	Neonatal mortality, neonatal admissions & OASI	..	22 January - 16 March 2020	17 March - 10 May 2020	..	11 May - 4 July 2020	3885	4147	Good
Quistorff 2021	cohort study	USA	Washington DC (Children's National Hospital, MedStar Washington)	Screening for anxiety in pregnancy	..	July 2014 - Feb 2020	May - September 2020	..	..	39	161	Fair
Racine 2022	cohort study	USA	Wisconsin (University of Wisconsin School of Medicine)	Neonatal admissions, emergency caesarean section, prolonged neonatal stay*, OASI, hysterectomy, prolonged maternal stay* &	..	15 December 2019 - 14 March 2020	15 March - 15 June 2020	..	..	1210	1058	Good

				maternal readmission								
Rao 2022	cohort study	USA	New York City (Mount Sinai Hospital)	Neonatal mortality, iatrogenic preterm birth, spontaneous preterm birth & planned cesarean section	..	27 March - 31 May 2019	27 March - 31 May 2020	..	..	1058	1384	Good
Rasmussen 2021	cohort study	Spain Italy Greece Germany UK Ireland Switzerla nd Belgium	Various single sites	Extreme preterm birth	..	Varied depending on country	Varied depending on country	..	..	3378	3536	Fair
Richter 2022	cohort study	USA	New York City (Mount	Neonatal admissions	..	December 16 – June,	16 March - 8 June 2020	..	From 8 June 2020	2558	18849	Fair

			Sinai Hospital)			2012 – 2019						
Riley 2021	cohort study	USA	Pacific Northwest (Washington, Oregon, Idaho)	Neonatal admissions & maternal readmission	..	January 1 2019 - Feb 29 2020	1 March - 31 December 2020	..	..	20704	78718	Good
Roberts 2022	cohort study	Canada	Ontario (BORN records)	Neonatal admissions, cesarean section, iatrogenic & spontaneous preterm birth	..	1 March 2015 - 29 February 2020	1 March - 31 October 2020	..	..	93711	702419	Good
Rolnik 2021	cohort study	Australia	Melbourne (Monash Health)	Low birthweight (very & SGA), neonatal mortality, prolonged neonatal stay & caesarean section (planned & emergency)	..	Conceived between 1 November 2018 - 28 February 2019	Conceived between 1 November 2019 - 29 February 2020	..	..	3187	3229	Good
Rusconi 2023	cohort study	Italy	all	Preterm birth (extreme,	..	January 2017 -	1 March 2020 - 31 March 2021	..	..	363089	1120176	Good

				very, & moderate to late) & stillbirth		February 2020						
Sangtani 2021	cohort study	USA	Michigan (Ann Arbor University of Michigan Medicine)	Screening for postpartum depression	..	April 2019	April 2020	..	..	387	390	Fair
Shah 2021	cohort study	Canada	Ontario (CIHIDAD)	Preterm birth (extreme, very & moderate to late)	Deprivation	July 2002 - December 2019	January - December 2020	..	..	129502	2316712	Good
Shukla 2022	cohort study	USA	Alabama	Hysterectomy	..	January – December, 2016 – 2019 & January – Februry 2020	March - December 2020 & January - June 2021	..	..	74076	236481	Good
Shukla 2023	cohort study	USA	Alabama	Stillbirth, neonatal mortality & neonatal admissions	..	January – December, 2016 - 2019	March 2020 - June 2021	July - September 2021	..	74076	236481	Good
Silverman 2020	cross-sectional study	USA	New York City (Mount	Screening for	..	2 January - 12 March 2020	March 13 - June 30 2020	..	..	252	264	Fair

			Sinai Hospital)	postpartum depression								
Simoene 2022	cohort study	USA	all Premier Healthcare database	Stillbirth & maternal readmission	..	1 April - 31 December 2019	1 April - 31 December 2020	..	..	614093	663620	Good
Simpson 2021	cohort study	Canada	Ontario (ICES records)	Neonatal mortality & SGA	..	15 March - 30 September , 2015 - 2019	15 March - 30 Septmeber 2020	..	..	67400	346834	Good
Sinnott 2021	cross-sectional study	USA	Boston, Massachusetts (Brigham and Women's Hospital)	Planned & emergency cesarean section	..	April - July 2019	April July 2020	..	..	1019	890	Fair
Smorti 2022	cohort study	Italy	Tuscany (University Hospital in Tuscany)	Screening for postpartum depression	..	October - December 2019	..	24 October - 3 December 2020	..	78	77	Fair
Snelgrove 2022	cross-sectional study	Canada	Ontario (ICES records)	Sepsis	..	15 March - 30 September , 2015-2019	15 March 2020 - 30 September 2021	..	..	157779	563859	Good
Speyer 2022	cohort study	Scotland	all	Stillbirth & cesarean section	..	March - May 2018	March - May 2020	..	..	7342	8323	Good

				(planned & emergency)								
Stumpfe 2022	cohort study	Germany	Bavaria	Preterm birth (extreme & very) & low birthweight (extreme & very)	..	2010 - 2019	16 March - 6 May 2020	2 November - 31 December 2020	..	16015	145018	Good
Sun 2021	cross-sectional study	USA	OptumLabs Data Warehouse, Medicare Advantage enrollees	Stillbirth	..	1 March - 31 December 2019	1 March - 31 December 2020	..	..	152903	172095	Fair
Suzuki 2020	cohort study	Japan	Katsushika (Japanese Red Cross Katsushika Maternity hospital)	Screening for postpartum depression & screening for anxiety in pregnancy	..	9 March - 11 April 2019	11 March - 13 April 2020	..	..	138	148	Fair
Suzuki 2020	cohort study	Japan	Katsushika (Japanese Red Cross Katsushika Maternity hospital)	Cesarean section	..	9 March - 11 April 2019	11 March - 13 April 2020	..	..	138	148	Fair

Tate 2023	cohort study	USA	Tenessee	Stillbirth	..	undefined	March 2020 - March 2021	..	..	2590	2921	Good
Vacaru 2021	cohort study	Netherlands	all	Screening for depression in pregnancy & screening for anxiety in pregnancy	..	March 2017 - September 2018	4 April - 10 May 2020	..	..	307	1337	Poor
Vitale 2021	cross-sectional study	Italy	Bari (Mental Health Centre of Modugno)	Screening for postpartum depression & screening for anxiety in pregnancy	..	Before November 2019	From December 2019	..	..	156	110	Fair
Wagner 2021	cohort study	Austria	all Austrian Perinatal registry	Neonatal admissions, cesarean section, OASI, hysterectomy & sepsis	..	1 January 2015 - 30 June 2019	1 January - 30 June 2020	..	..	33198	188225	Good
Waschmann 2021	cohort study	USA	Oregon (Oregon Health and Science University)	Screening for postpartum depression	..	1 January - 1 June 2019	1 January - 1 June 2020	..	..	504	557	Fair

Wikarek 2022	cross-sectional study	Poland	Katowice (University Clinical Centre of the Medical University of Silesia)	Screening for postpartum depression & screening for anxiety in pregnancy	..	January 2019 - March 2020	..	April - November 2021	..	275	190	Fair
Wood 2021	cohort study	USA	Massachusetts (Brigham Boston General)	Iatrogenic, spontaneous, extreme & very preterm birth	..	April - July 2019	April - July 2020	..	..	4712	4644	Good
Zanardo 2020	case control study	Italy	Padua (Policlinico Abano Terme)	Caesarean section (planned & emergency) & screening for postpartum depression	..	8 March - 3 May 2019	8 March - 3 May 2020	..	..	91	101	Fair
Zhang 2022	cohort study	Canada	Toronto (Mount Sinai Hospital)	Screening for depression in pregnancy,	..	1 March 2019 - 29 February 2020	From March 2020	..	..	110	263	Fair

				screening for postpartum depression, screening for anxiety in pregnancy & screening for postpartum anxiety								
Zilver 2021	cohort study	Netherlands	Amsterdam (ILVG hospital and online)	Screening for depression in pregnancy & screening for anxiety in pregnancy	..	February 2019 - January 2020	21 May - 22 June 2020		..	1102	364	Fair

*Outcomes reported as part of overall and which had subclassification outcomes included have subclassification outcomes in brackets, Gurol-Urganci et al. ethnicity data excluded as more detailed ethnicity data from Ibrahim unpublished study. OASI (obstetric anal sphincter tears), SGA (small for gestational age), HIE (Hypoxic Ischemic Encephalopathy), prolonged maternal or neonatal stay in hospital

Supplementary Material 5: Tables of studies included in time-adjusted meta-analysis with key characteristics & risk of bias assessment.

	**Country**	**Region**	**Site**	**Outcomes included**	**Pre-lockdown pandemic** (dates)	**Lockdown 1** (dates)	**Sample Size**	**Newcastle Ottawa Overall Bias Assessment** (Poor, Fair, Good)
Been 2020	Netherlands	all	all	Preterm birth	9 October 2010 - 9 March 2020	9 March - 16 July 2020	796531	Good
Fresson 2022	France	all	all	Preterm birth & stillbirth	1 January 2016 - 17 March 2020	17 March - 10 May 2020	3179532	Good
Maeda 2020	Japan	all	all	Preterm birth	2 March - 17 April 2019	2 March - 17 April 2020	28474	Good
Morgan 2022	England	Yorkshire & Humber	all	Preterm birth & stillbirth	29 December 2014 - 22 March 2020	23 March 2020 - 14 June 2020	1161*	Good

Stansfield 2022	Australia	Melbourne	Monash Health Victoria	Preterm birth	1 January 2018 - 15 March 2020	16 March 2020 - 21 October 2021	35660	Good
Stumpfe 2022	Germany	Bavaria	all	Preterm birth	2010 - 2019	16 March - 6 May 2020	1263959	Good
Stumpfe 2022	Germany	Bavaria	all	Stillbirth	2010 - 2019	16 March - 6 May 2020	..	Good
Rusconi 2023	Italy	all	0	Preterm birth	January 2017 - February 2020	1 March 2020 - 31 March 2021	1479301	Good
Hwang 2022	South Korea	all	all	Preterm birth	January 2011 - December 2019	January - Demember 2020	3736447	Good
Balsa 2022	Uruguay	all	all	Stillbirth *	December 5 2018 - December 4 2020	March 14 - December 4 2020	..	Good
Calvert 2023	Australia	New South Wales	all	Preterm birth & stillbirth	2015-2020	23.Mar.20	518281	Good

Denmark	central region	all	Preterm birth	2016 - 2020	13.Mar.20	66481	Good
Belgium	all	all	Preterm birth & stillbirth	2015-2020	14.Mar.20	665086	Good
Finland	all	all	Preterm birth & stillbirth	2015-2020	16.Mar.20	278376	Good
Hungary	all	all	Preterm birth & stillbirth	2015-2020	12.Mar.20	501860	Good
Iceland	all	all	Preterm birth & stillbirth	2015-2020	16.Mar.20	23463	Good
Norway	all	all	Preterm birth & stillbirth	2015-2020	15.Mar.20	316067	Good
Scotland	all	all	Preterm birth & stillbirth	2015-2020	22.Mar.20	288118	Good
Switzerland	all	all	Preterm birth & stillbirth	2015-2020	17.Mar.20	486357	Good
Wales	all	all	Preterm birth & stillbirth	2015-2020	22.Mar.20	176964	Good
Canada	all (exlcuding Quebec)	all	Preterm birth	2015-2020	18.Mar.20	1610511	Good
USA	all	all	Preterm birth	2015-2020	16.Mar.20	20979669	Good
Chile	all	all	Preterm birth	2015-2020	18.Mar.20	1244121	Good

	Uruguay	all	all	Preterm birth	2015-2020	15.Mar.20	232514	Good
	Australia	Queensland	one hospital	Preterm birth	2015-2020	23.Mar.20	58204	Good
	Hong Kong	Hong Kong	all public facilities (pooled)	Preterm birth	2015-2020	08.Feb.20	199134	Good
	Poland	poland	Facility 1	Preterm birth & stillbirth	2015-2020	15.Mar.20	8287	Good
	Poland	poland	Facility 2	Preterm birth & stillbirth	2015-2020	15.Mar.20	42243	Good

*Morgan, 2022. only reported mean total deliveries (authors emailed no response). Balsa, 2022 preterm birth data excluded due to cohort overlap with Calvert, 2023.

Supplementary Material 6: PRISMA Flowchart of study selection

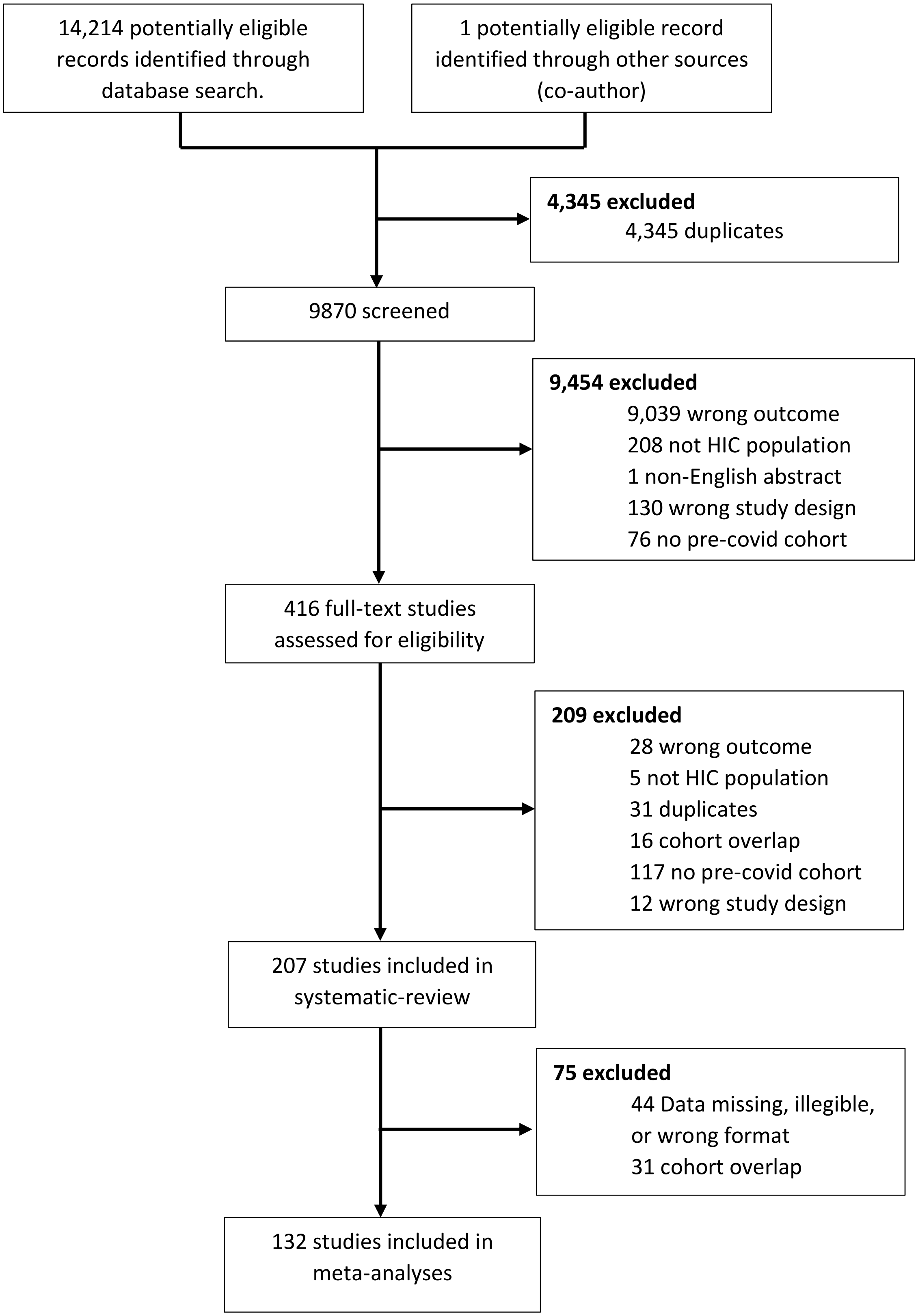

Supplementary Material 7: meta-analyses of outcomes by sub-groups and subclassifications

Perinatal/Neonatal Outcomes

Preterm Birth:

Sub-group analysis of preterm birth (PTB): by second lockdown and post-lockdown time periods

Below is the subgroup analysis of preterm birth (PTB) by distinct second lockdown and post-lockdown time periods. Only PTB had presented data on second and post-lockdown periods in more than three studies, warranting a sub-group meta-analysis. There was no evidence to support an association between PTB and the time periods defined as second lockdowns (RR0·95[95%CI0·90-1·00]), heterogeneity was considerable at 89.8% (Supplementary Figure 7: Panel A). The was no credible evidence to support an association between post-lockdown period and preterm birth (RR0·98[95%CI0·93-1·04]) (Supplementary Figure 7: Panel B).

Sub-group analysis of PTB: by sub-classifications of PTB iatrogenic & spontaneous

Within PTB there are two classifications, iatrogenic (medically induced) or spontaneous. Spontaneous PTB was associated with a decrease over lockdown (RR0·95[95%CI0·90- 0·99]) (Supplementary Figure 8: Panel A), whereas iatrogenic PTB indicated no evidence of an association with the lockdown period (RR0·95[95%CI0·88-1·03]) (Supplementary Figure 8: Panel B).

Sub-group analysis of PTB: by sub-classifications of extreme, very, and moderate-late PTB

Meta-analysis of extreme PTB included 28 studies, which indicated no evidence of an association with lockdown (RR0·97[95%CI0·91-1.03]) (Supplementary Figure 9: Panel A). Very PTB, reported by 28 studies, also suggested no evidence of an association the lockdown period (RR0·93[95%CI0·83-1·04]) (Supplementary Figure 9: Panel B). Moderate to late PTB was reported by 21 studies, and had credible evidence of a decrease (6%) associated with the lockdown period (RR0·94[95%CI0·92-0.97]) (Supplementary Figure 9: Panel C).

Low birthweight:

Subgroup analysis of low birthweight: by sub-classification

Small for gestational age (SGA), reported by ten studies indicated no evidence of an association with the lockdown period (RR0·96[95%CI0·92-1·01]) (Supplementary Figure 10: Panel A). Very low birthweight (VLBW) was reported by 13 studies also indicated no evidence of an association with lockdown (RR0·91[95%CI0·81-1·03]) (Supplementary Figure 10: Panel B). Extreme low birthweight (ELBW) was reported by ten studies, and again showed insufficient evidence to support an association with lockdown (RR0·80[95%CI0·59-1·08]) (Supplementary Figure 10: Panel C).

Subgroup analysis of low birthweight combined outcome: by region

Due to the considerable heterogeneity (I^2=81.9%) in the overall low birthweight outcome meta-analysis, regional subgroup analysis by continent was run, this indicated no sub-group effect by region (p=0.292) (Supplementary Figure 10: Panel D).

Neonatal admissions:

Subgroup analysis of neonatal admission: by region

Due to the considerable heterogeneity (I^2=88.2%) in the neonatal admissions meta-analysis, regional subgroup analysis by continent was run. The test for subgroup differences supported that the effect differed by region (p=0.018). This analysis supported that lockdown was associated with a decrease in neonatal admissions in the Middle East (RR0·84[95%CI0·72-0·97]) and associated with an increase in Asia over lockdown (RR1·38[95%CI1·05-1·83]) (Supplementary Figure 11).

Maternal Health Outcomes

Caesarean Section:

Subgroup analysis of caesarean section: planned and emergency.

Planned caesarean section was reported by 15 studies and meta-analysis found no credible evidence of an association with the lockdown period (RR1·03[95%CI0·99-1.07]) (Supplementary Figure 12: Panel A). Emergency caesarean section was reported by 16 studies and also showed no credible evidence of an association with lockdown (RR0·99[95%CI0·97-1·02]) (Supplementary Figure 12: Panel B).

Subgroup analysis of caesarean section overall: by region

Due to high heterogeneity between studies in the overall caesarean section meta-analysis (I^2=82.8%), sub-group analysis by region was run. The test for subgroup differences suggested no evidence of a subgroup effect by region (p=0.189) (Supplementary Figure 12: Panel C).

Maternal Readmission

Subgroup analysis of maternal readmission: by region

Maternal readmission had considerable heterogeneity (I^2=97%) in the overall meta-analysis, therefore regional subgroup analysis was run. Regional subgroup analysis found associations between maternal readmission and lockdown differed by continental region (p<0.001). There was a decrease in maternal readmission in Europe only, specifically the UK, associated with lockdown compared to pre-lockdown (RR0.69[95%CI0.66-0.71]), no credible evidence supported an association with lockdown in North America (RR1.02[95%CI0.99-1.05]) or the Middle East (RR1.40[95%CI0.67-2.90])(Supplementary Figure 13).

Maternal Mental Health Outcomes

Sub-group analysis of maternal mental health outcomes: by region

Due to considerable heterogeneity between studies on screening for depression antenatally (I^2=75.9%), sub-group analysis by region was run (Supplementary Figure 14: Panel A). We also ran regional subgroup analysis due to considerable heterogeneity in meta-analysis of screening results for maternal anxiety antenatally (I^2=89.4%) (Supplementary Figure 14: Panel B). There was insufficient evidence to support that associations differed by continental region for either outcome (depression antenatally [p=0.662]) & anxiety antenatally [p=0.505]).

Supplementary Material 8: funnel plots and Egger's test results

Supplementary material 2 presents the results of investigation into publication bias and small study effects in supplementary meta-analyses which included over 10 studies. There was weak to no evidence of publication bias in any outcomes included in supplementary analyses (Supplementary Figure 15: Panel A-H).

Supplementary Material 9: Power calculations and minimum detectable effects.

The following outcomes and subgroups were sufficiently powered (>80%) to investigate the pooled effect estimate obtained by meta-analyses. Each outcome or subgroup is reported with the estimated power of the sample to investigate the pooled estimate and minimum detectable risk ratio, both in parentheses:

- PTB and second lockdown (100%, 0.9917)
- Spontaneous PTB (96%, 0.9620)
- Iatrogenic PTB (95%, 0.9614)
- Very PTB (100%, 0.9686)
- LBW subgroup analysis by region:
    - North America (100%, 0.9921)
    - Europe (88%, 0.9372)
    - Asia (97%, 1.0292)
- SGA (100%, 0.9818)
- Very LBW (100%, 0.9808)
- Extreme LBW (92%, 0.8311)
- Neonatal admissions subgroup analysis by region:
    - North America (100%, 1.0169)
- Cesarean section subgroup analysis by region:
    - North America (100%, 1.0033)
    - Asia (81%, 1.0791)
- Planned (elective) cesarean section (97%, 1.0209)
- Positive screening for maternal depression antenatally subgroup analysis by region:
    - North America (99%, 1.2432)
- Positive screening for maternal anxiety antenatally subgroup analysis by region:
    - North America (95%, 1.1991)

The following outcomes and subgroups were not sufficiently powered (<80%) to investigate the pooled effect estimate obtained by meta-analyses:

- PTB and post-lockdown (16%, 0.9414)
- Extreme PTB (43%, 0.9533)
- LBW subgroup analysis by region:
    - Middle East (44%, 0.7000)
    - Australia (37%, 0.7998)
    - South America (28%, 0.9200)
- Neonatal admissions subgroup analysis by region:
    - Europe (49%, 1.0433)
    - Asia (5%, 1.1498)
- Cesarean section subgroup analysis by region:
    - Europe (55%, 0.9867)
    - Middle East (8%, 1.0561)
    - Australia (26%, 1.1066)

    - South America (65%, 1.0238)
- Emergency cesarean section (43%, 0.9842)
- Maternal readmission by region:
    - North America (27%, 1.0418)
    - Middle East (15%, 1.4614)
- Positive screening for maternal depression antenatally subgroup analysis by region:
    - Europe (65%, 1.2896)
    - Australia (8%, 1.7674)
- Positive screening for maternal anxiety antenatally subgroup analysis by region:
    - Europe (68%, 0.8514)
    - Asia (9%, 1.7339)
    - Australia (5%, 1.2654)

Supplementary Material 10: studies excluded from meta-analysis with brief reasons.

Studies excluded from meta-analysis with brief reasons

Studies excluded due to cohort overlap (data duplication risk)

Study's first author's last name, year of publication, "title"	Reason for exclusion
Puertas-Gonzalez, 2021, "Giving birth during a pandemic: From elation to psychopathology"	- Data duplication risk (used data from same data source as Marino-Narvaez publication: Gestastress cohort)<br>- Dropped in favour of Marino-Narvaez study as the later study data which better suited the study criteria (number of individuals with clinically relevant postpartum depression score)
Vani, 2022, "Characteristics of stillbirths during the Coronavirus 2019 (COVID-19) pandemic"	- Data duplication risk with studies providing national data on stillbirths in the USA
Graff, 2022, "COVID-19 and lockdown: Impact on pregnancy complications"	- Data duplication risk with Fresson, 2022
Kern-Goldberger, 2022, "Patterns of Prenatal Care Delivery and Obstetric Outcomes Before and During the COVID-19 Pandemic"	- Data duplication risk with Gemmill, 2021
Bunnell, 2021, "Third trimester stillbirth during the first wave of the SARS-CoV-2 pandemic: Similar rates with increase in placental vasculopathic pathology"	- Data duplication risk with Chen, 2022<br>- No total values for stratified data<br>- Emailed with no response
Simon, 2021, "Impact of the COVID-19 pandemic on preterm birth and stillbirth: a nationwide, population-based retrospective cohort study"	- Data duplication risk with Fresson, 2022
Hugh, 2021, "Effect of COVID-19 lockdown on fetal growth surveillance, prematurity and stillbirth rates"	- Data duplication risk with Gurol-Urganci, 2022
Stowe, 2021, "Stillbirths During the COVID-19 Pandemic in England, April-June 2020"	- Data duplication risk with Gurol-Urganci, 2022
Datta, 2021, "Covid-19 pandemic is not associated with an increased risk of perinatal mortality"	- Data duplication risk with Gurol-Urganci, 2022
Ucyigit, 2021, "Maternity outcomes during the Covid-19 pandemic: A single-site controlled retrospective observational study"	- Data duplication risk with Gurol-Urganci, 2022<br>- No total aggregated data provided
Hedermann, 2021, "Danish premature birth rates during the COVID-19 lockdown"	- Data duplication risk with Molholm-Hansen, 2022
Rolnik, 2021, "11 Impact of the coronavirus pandemic lockdown on obstetric outcomes"	- Data duplication risk with Rolnik, 2021
Matheson, 2021, "Prematurity Rates During the Coronavirus Disease 2019 (COVID-19) Pandemic Lockdown in Melbourne, Australia"	- Data duplication risk with Rolnik, 2021

Stansfield, 2022, "Impact of the COVID-19 pandemic and multiple community lockdowns on total live birth rates and preterm births in Melbourne, Australia"	- Data duplication risk with Rolnik, 2021<br>- Data on total live births and number of outcomes missing
Mulcahy, 2021, "Preterm Infant Outcomes Following COVID-19 Lockdowns in Melbourne, Australia"	- Data duplication risk with Rolnik, 2021
Barton, 2022, "Induction of labour during the first wave of COVID-19 and its impact"	- Data duplication risk with UK Gurol-Urganci, 2022.
Briozzo, 2023, "COVID-19 mitigation measures increase preterm birth and low birth weight in the public healthcare system in Uruguay"	- Data duplication risk with Balsa, 2022.
Elfaday, 2022, "ROP severity and treatment rate during COVID-19 in UK"	- Data duplication risk with UK Gurol-Urganci, 2022.
Grobman, 2023, "The Temporal Relationship Between the Coronavirus Disease 2019 (COVID-19) Pandemic and Preterm Birth"	- Data duplication risk with Gemmill, 2021.
Gulersen, 2022, "Impact of COVID-19 pandemic on maternal and neonatal morbidities in the United States"	- Data duplication risk with Gemmill, 2021.
Johnson, 2023, "Impact of the COVID-19 Pandemic on Obstetric Interventions at a Public Hospital"	- Data duplication risk with Gemmill, 2021.
Le Ray, 2022, "Results of the 2021 French National Perinatal Survey and trends in perinatal health in metropolitan France since 1995"	- Data duplication risk with Fresson, 2022.<br>- Excluded in favor of Fresson et al, as reported on fewer outcomes of interest and did not focus on lockdown period as intervention.
Margerison, 2023, "Exposure to the early COVID-19 pandemic and early, moderate and overall preterm births in the United States: A conception cohort approach"	- Data duplication risk with Gemmill, 2021.<br>- No aggregated data readily available so excluded in favor of Gemmill, et al. study.
Lorenzi, 2023, "An evaluation of the association between lockdown during the SARS-CoV-2 pandemic and prematurity at the Nice University Hospital"	- Data duplication risk with Fresson, 2022.
Molina, 2022, "Comparison of Pregnancy and Birth Outcomes Before vs During the COVID-19 Pandemic"	- Data duplication risk with Gemmill, 2021.
Rodriguez, 2023, "Are preterm birth and very low birth weight rates altered in the early COVID (2020) SARS-CoV-2 era?"	- Data duplication risk with Gemmill, 2021.
Sadeghi, 2022, "Comparing postnatal readmissions during COVID-19 and pre-pandemic in a tertiary care hospital"	- Data duplication risk with UK Gurol-Urganci, 2022.
Kniffka, 2021, "Stillbirths in Germany: On the rise, but no additional increases during the first COVID-19 lockdown"	- Data duplication risk with Lau, 2023.
Stumpfe, 2022, "Lack of evidence for effects of lockdowns on stillbirth rates during the SARS-CoV-2 pandemic in Bavaria: analysis of the Bavarian perinatal survey from 2010 to 2020"	- Data duplication risk with Lau, 2023.
Puertas-Gonzalez, 2021, "The psychological impact of the COVID-19 pandemic on pregnant women"	- Data duplication risk with Marino-Narvaez, 2021.<br>- Only mean scores for depression reported not aggregated totals of those screened positive
Amadori, 2021, "Obstetrics and gynecology emergency department activity during lockdown: What did we learn?"	- Data duplication risk with Rusconi, 2023.

Studies excluded as data missing, illegible, or wrongly formatted

**Study's first author's last name, year of publication, "title"**	- **Reason for exclusion**
Stansfield, 2022, "Impact of the COVID-19 pandemic and multiple community lockdowns on total live birth rates and preterm births in Melbourne, Australia"	- Data duplication risk with Rolnik, 2021<br>- Data on total live births and number of outcomes missing
Liu, 2022, "Characteristics of Preterm Births in the Setting of Reduced Preterm Birth Rates During COVID-19 Lockdown"	- Data missing on key outcomes (only data on premature rupture of membranes)
Ucyigit, 2021, "Maternity outcomes during the Covid-19 pandemic: A single-site controlled retrospective observational study"	- Data duplication risk with Gurol-Urganci, 2022<br>- No total aggregated data provided
Marques-Fernandez, 2021, "Impact of Covid-19 on attendances for a 1st episode of reduced foetal movements: A retrospective observational study"	- Population total only included mothers with reduce foetal movement (no total deliveries)
Brown, 2021, "P.70 Stay home: the effect of COVID-19 on post-operative length of stay in obstetric patients"	- Population total only included mothers who had undergone a cesarean section (no total deliveries)
Licheni, 2022, "Impact of COVID-19 public health restrictions on hospital admissions for young infants in Victoria, Australia"	- Not outcome of interest (infants less than three months admitted to general medicine)
Vatcheva, 2021, "Impact of COVID-19 pandemic on postpartum depression among mothers of extreme and early preterm infants"	- Population total only included mothers who gave birth preterm (no total live births)
Hidalgo-Lopezosa, 2021, "Vaginal birth after caesarean section before and during COVID-19 pandemic. Factors associated with successful vaginal birth"	- Population total only included women with previous cesarean section (no total live births)<br>-
Niela-Vilen, 2021, "Pregnant women's daily patterns of well-being before and during the COVID-19 pandemic in Finland: Longitudinal monitoring through smartwatch technology"	- Data reported in the wrong format and not combinable with other outcomes
Price, 2022, "Neonatal morbidity after the start of the pandemic: Disparate outcomes across two sites"	- Data reported in the wrong format and not combinable with other outcomes
Maeda, 2021, "Trends in intensive neonatal care during the COVID-19 outbreak in Japan"	- Missing information on total births per time of interest<br>- Emailed with no response
El-Helou, 2022, "Impact of the COVID-19 pandemic on outpatient postpartum care utilization"	- Data on total number of live births and outcomes per times of interest not presented, only reported adjusted odds ratios<br>- Emailed with no response
Morgan, 2022, "Effects of the SARS-CoV-2 pandemic on perinatal activity in Yorkshire and the Humber region during 2020: an interrupted time series analysis"	- Data reported in the wrong format for meta-analysis (means)<br>- Emailed no response

Oakes, 2021, "Changes in the Antenatal Utilization of High-Risk Obstetric Services and Stillbirth Rate during the COVID-19 Pandemic"	- Data illegible in format available, full text not available online<br>- Insufficient information to calculate the total stillbirths per time of interest<br>- Emailed with no response
Tramontano, 2021, "First wave of COVID-19 pandemic and caesarean delivery rate in Italy"	- No total aggregated data provided, just odds ratios<br>- Emailed with no response
Hedley, 2022, "Preterm birth, stillbirth and early neonatal mortality during the Danish COVID-19 lockdown"	- Data reported in the wrong format for meta-analysis (means)<br>- Emailed no response
Venkata, 2022, "Outcomes of infants with hypoxic-ischemic encephalopathy during COVID-19 pandemic lockdown in Canada: a cohort study"	- Data on total live births missing.<br>- Emailed for total births data but no response
Melov, 2022, "INVESTIGATING PERINATAL OUTCOMES DURING THE LOW PREVALENCE FIRST YEAR OF COVID-19 IN A MULTIETHNIC AUSTRALIAN POPULATION"	- Data missing on number of preterm births in each time of interest.<br>- Insufficient information to calculate and no email response
Obata, 2022, "Changes in fetal growth restriction and retinopathy of prematurity during the coronavirus disease 2019 pandemic: A crosssectional study"	- Data missing on total live births, only neonatal admission totals reported.
Aboulatta, 2023, "Stillbirth Rates before and during the COVID-19 Pandemic in Manitoba (Canada) and Japan"	- No aggregated data on outcomes or livebirths reported.<br>- Authors emailed with no response.
Brow, 2022, "The Burden of Maternal Mental Health During COVID-19: Impact Analysis Through Perinatal Mood and Anxiety Disorders Screening"	- Aggregated and total values of outcomes not reported.<br>- Author emailed with no response.
Camoni, 2022, "The Impact of the COVID-19 Pandemic on Women's Perinatal Mental Health: Preliminary Data on the Risk of Perinatal Depression/Anxiety from a National Survey in Italy"	- Aggregated number of total outcomes per pregnant and postpartum individuals not reported.<br>- Authors emailed with no response.
Chieffo, 2022, "Pregnancy-related psychopathology: A comparison between preCOVID-19 and COVID-19–related social restriction period"	- Data reported as medians and confidence intervals, not total outcomes.
Do, 2023, "EPH174 Trends in Postpartum Depression before and during the COVID-19 Pandemic and Correlates of Treatment Choices: A Population-Based Retrospective Cohort Study"	- No aggregated data reported on outcomes.<br>- No corresponding email shared or identified.
Greenberg, 2023, "COVID-19 related changes in perinatal health care delivery and outcomes among pregnant individuals and newborns"	- No aggregated data reported on outcomes.<br>- Authors emailed with no response.
McCracken, 2022, "COVID-19 and Its Effect on the Prevalence of Postpartum Anxiety"	- No aggregated data reported on outcomes.

	- No corresponding email shared or identified.
Metz, 2023, “Association Between Giving Birth During the Early Coronavirus Disease 2019 (COVID-19) Pandemic and Serious Maternal Morbidity”	- No aggregated data reported on outcomes.<br>- Authors emailed with no response.
Pinto, 2022, “Anxiety and depressive symptoms, and positive and negative couple interactions among postpartum mothers and fathers before and during the COVID-19 pandemic”	- Data reported as mean scores, no aggregated totals of outcomes provided.
Twichell, 2022, “The Effect of the COVID-19 Pandemic on EPDS Scores and Maternal Quality of Life”	- No data on total number of pregnant and postpartum women per period reported.<br>- No corresponding email shared or identified.
Tsukuru, 2023, “Single-center retrospective study for change of perinatal situation in COVID-19 pandemic”	- No aggregated data reported on outcomes.<br>- No corresponding email shared or identified.
Zagkos, 2022, “Prematurity, Neonatal Intensive Care Unit Admissions and Birth Rates In Four Major Greek Hospitals During the Strictest Quarantine Periods of the COVID-19 Pandemic”	- No aggregated data reported on outcomes.<br>- No corresponding email shared or identified.
Takubo, 2021, “Psychological impacts of the COVID-19 pandemic on one-month postpartum mothers in a metropolitan area of Japan”	- No aggregated data reported on outcomes, depression reported as mean scores only.
Silverman, 2020, “Early pregnancy mood before and during COVID-19 community restrictions among women of low socioeconomic status in New York City: a preliminary study”	- No aggregated data reported on outcomes, depression reported as mean scores only.
Silvagni, 2021, “Neonatal and pediatric emergency room visits in a tertiary center during the COVID-19 pandemic in Italy”	- Data missing on total livebirths, only admissions to neonatal and pediatric emergency room included
Rigamonti, 2021, “COVID 19: Aspetti materni, fetali, neonatali e organizzativi Perinatal Depression Screening in ASST Brianza: Longitudinal analysis pre and during Covid 19 pandemia”	- Data missing on separate number of pregnant and postpartum individuals, only aggregated variable of perinatal depression available.
Perez, 2021, “Experience of early motherhood during the first wave of the COVID-19 pandemic in Northern Germany: a single-centre before and after comparison”	- No aggregated data reported on outcomes, data reported as mean scores only.
Overbeck, 2021, “Depression and anxiety symptoms in pregnant women in Denmark during COVID-19”	- No aggregated data reported on outcomes, data reported as mean scores only.
Morris, 2021, “Mental Health and Prenatal Bonding in Pregnant Women During the COVID-19 Pandemic: Evidence for Heightened Risk Compared With a Prepandemic Sample”	- No aggregated data reported on outcomes, data reported as mean scores only.
McFarland, 2021, “Postpartum Depressive Symptoms during the Beginning of the COVID-19 Pandemic: An Examination of Population Birth Data from Central New Jersey”	- No aggregated data reported on outcomes, data reported as mean scores only.
Ladekarl, 2021, “Early Postpartum Stress, Anxiety, Depression, and Resilience Development among Danish First-Time Mothers before and during First-Wave COVID-19 Pandemic”	- No aggregated data reported on outcomes, data reported as mean scores only.

Greenbury, 2021, "Changes in neonatal admissions, care processes and outcomes in England and Wales during the COVID-19 pandemic: a whole population cohort study"	- Data missing on total livebirths, only admissions to neonatal unit included. Excluded in favor of Gurol-Urganci, 2022.
Almeida, 2022, "COVID-19 pandemic lockdown effect on neonatal hospital admissions from the community"	- Data missing on total livebirths, only admissions to neonatal care unit from emergency department included.
Calvert, 2023, "Changes in preterm birth and stillbirth during COVID-19 lockdowns in 26 countries"	- No aggregated data reported on outcomes, only pooled adjusted estimates
Kakarapathi, 2022, "Anxiety, depression, worry and stress-related perceptions among antenatal women during the COVID-19 pandemic: Single group repeated measures design"	- No aggregated data reported on outcomes, data reported as mean scores only.

Studies excluded from time adjusted analysis with brief reasons

**Study's first author's last name, year of publication, "title"**	**Reason for exclusion**
Aboulatta, 2022, "COVID-19 pandemic impact on preterm birth and stillbirth rates associated with socioeconomic disparities: A quasi-experimental study"	- Time-adjusted ratio estimates not presented in publication; authors emailed with no response.<br>- Cohort overlap (data duplication risk) with Calvert, 2023.
Greenbury, 2021, "Changes in neonatal admissions, care processes and outcomes in England and Wales during the COVID-19 pandemic: a whole population cohort study"	- Absolute magnitude of change reported not time-adjusted ratio estimates.<br>- Cohort overlap (data duplication risk) with Calvert, 2023.
Kniffka, 2021, "Stillbirths in Germany: On the rise, but no additional increases during the first COVID-19 lockdown"	- Time-adjusted ratio estimates not presented in publication.<br>- 2020 data based on predicted estimates, not observed values.<br>- Cohort overlap (data duplication risk) with Calvert, 2023.
Gemmill, 2021, "Changes in preterm birth and caesarean deliveries in the United States during the SARS-CoV-2 pandemic"	- Time-adjusted ratio estimates not presented in publication; authors emailed with no response.<br>- Cohort overlap (data duplication risk) with Calvert, 2023.

Oakley, 2022, “Preterm birth after the introduction of COVID-19 mitigation measures in Norway, Sweden, and Denmark: a registry-based difference-in-differences study”	- Absolute magnitude of change reported not time-adjusted ratio estimates.<br>- Cohort overlap (data duplication risk) with Calvert, 2023.
Philip, 2020, “Unprecedented reduction in births of very low birthweight (VLBW) and extremely low birthweight (ELBW) infants during the COVID-19 lockdown in Ireland: a 'natural experiment' allowing analysis of data from the prior two decades”	- Time-adjusted ratio estimates not presented in publication; authors emailed with no response.
Richter, 2022, “Neonatal outcomes during the COVID-19 pandemic in New York City”	- Cohort overlap (data duplication risk) with Calvert, 2023.
Riley, 2021, “Impact of the COVID-19 pandemic on perinatal care and outcomes in the United States: An interrupted time series analysis”	- Cohort overlap (data duplication risk) with Calvert, 2023.
Roberts, 2021, “Maternal-Newborn Health System Changes and Outcomes in Ontario, Canada, During Wave 1 of the COVID-19 Pandemic—A Retrospective Study”	- Cohort overlap (data duplication risk) with Calvert, 2023.
Shukla, 2023, “Trends in fetal and neonatal outcomes during the COVID-19 pandemic in Alabama”	- Cohort overlap (data duplication risk) with Calvert, 2023.
Margerison, 2022, “Exposure to the early COVID-19 pandemic and early, moderate and overall preterm births in the United States: A conception cohort approach”	- Cohort overlap (data duplication risk) with Calvert, 2023.
Stumpfe, 2022, “Lack of evidence for effects of lockdowns on stillbirth rates during the SARS-CoV-2 pandemic in Bavaria: analysis of the Bavarian perinatal survey from 2010 to 2020”	- Cohort overlap (data duplication risk) with Calvert, 2023.
Stumpfe, 2022, “Limited Effects of SARS-CoV-2 Pandemic-related Lockdowns and Reduced Population Mobility on Preterm Birth Rates: A Secondary Analysis of Bavarian Obstetric Quality Parameters from 2010 to 2020”	- Cohort overlap (data duplication risk) with Calvert, 2023.

Supplementary Material 11: list of studies excluded at full text screening with brief reasons.

Studies excluded at full text screening (due to cohort overlap)

The following studies were excluded due to overlap in data source, dates, or site.

Marine Lorenzi and Mathilde Mayerus and Sergio Eleni Dit Trolli and Amandine Hue-Bigé and Kévin Legueult and Isabelle Guellec-Renne and Bérengère F-G. An evaluation of the association between lockdown during the SARS-CoV-2 pandemic and prematurity at the Nice University Hospital. *Frontiers in Pediatrics* 2023; **11**.

Sadeghi N, Kashif A, Weems C, Bhatia M. Comparing postnatal readmissions during COVID-19 and pre-pandemic in a tertiary care hospital. *BJOG: An International Journal of Obstetrics and Gynaecology* 2022; **129**: 150.

Barton K, Singh T, Johnstone J. Induction of labour during the first wave of COVID-19 and its impact. *BJOG: An International Journal of Obstetrics and Gynaecology* 2022; **129**: 83.

Elfadaly DD, Reddy MA, Patel H. ROP severity and treatment rate during COVID-19 in UK. *Journal of AAPOS* 2022; **26**(4): e19-e20.

Molina RL, Tsai TC, Dai D, et al. Comparison of Pregnancy and Birth Outcomes Before vs During the COVID-19 Pandemic. *JAMA Network Open* 2022; **5**(8): E2226531.

Gulersen M, Lenchner E, Grunebaum A, Chervenak FA, Bornstein E. Impact of COVID-19 pandemic on maternal and neonatal morbidities in the United States. *American Journal of Obstetrics and Gynecology MFM* 2022; **4**(5).

Wei J, Benedetto-Anzai MT, Sawai M, Cheon T, Anzai Y. Maternal race as a risk factor for maternal ICU admission, cesarean delivery, and maternal transfusion. *American Journal of Obstetrics and Gynecology* 2023; **228**(1): S331.

Briozzo L, Tomasso G, Trujillo J, et al. COVID-19 mitigation measures increase preterm birth and low birth weight in the public healthcare system in Uruguay. *International Journal of Gynecology and Obstetrics* 2023.

Johnson TA, Jamieson DJ, Geary FH, Stanhope KK, Boulet SL. Impact of the COVID-19 Pandemic on Obstetric Interventions at a Public Hospital. *Women's Health Issues* 2023; **33**(1): 10-6.

Margerison CE, Bruckner TA, MacCallum-Bridges C, Catalano R, Casey JA, Gemmill A. Exposure to the early COVID-19 pandemic and early, moderate and overall preterm births in the United States: A conception cohort approach. *Paediatric and Perinatal Epidemiology* 2023; **37**(2): 104-12.

Victoria EdK, Paula LH, Gitte H, et al. THE IMPACT OF THE COVID-19 LOCKDOWN ON BIRTH WEIGHT AMONG SINGLETON TERM BIRTHS IN DENMARK. *medRxiv* 2022: 2022.10.07.22280781.

Rodriguez K, Nudelman MJ, Jegatheesan P, et al. Are preterm birth and very low birth weight rates altered in the early COVID (2020) SARS-CoV-2 era? *Frontiers in Pediatrics* 2023; **10**.

Le Ray C, Lelong N, Cinelli H, Blondel B. Results of the 2021 French National Perinatal Survey and trends in perinatal health in metropolitan France since 1995. *J Gynecol Obstet Hum Reprod* 2022; **51**(10): 102509.

Grobman WA, Sandoval GJ, Metz TD, et al. The Temporal Relationship Between the Coronavirus Disease 2019 (COVID-19) Pandemic and Preterm Birth. *Obstet Gynecol* 2023.

Briozzo L, Tomasso G, Viroga S, et al. Effect of the unfavorable maternal-fetal environment cause by mitigation measures of the covid-19 pandemic in the public maternity of reference of Uruguay. *J Matern Fetal Neonatal Med* 2022; **35**(25): 7312-5.

Obata S, Matsumoto R, Kakinoki M, et al. Changes in fetal growth restriction and retinopathy of prematurity during the coronavirus disease 2019 pandemic: A crosssectional study. *PLoS ONE* 2022; **17**(3 March).

Studies excluded at full text screening (due to duplicate already included)

The following studies were excluded due to as they were duplicates of studies already included. Duplicates shared same authors, content, and population, but were published in different journals or under different titles.

Leibovitch L, Reichman B, Mimouni F, et al. Preterm Singleton Birth Rate during the COVID-19 Lockdown: A Population-Based Study. *Am J Perinatol* 2022; **39**(9): 1020-6.

Jasper B, Stillerova T, Anstey C, Weaver E. Reduction in preterm birth rates during and after the COVID-19 lockdown in Queensland Australia. *Aust N Z J Obstet Gynaecol* 2022; **62**(6): 851-8.

Mullin AM, Handley SC, Lundsberg L, et al. Changes in preterm birth during the COVID-19 pandemic by duration of exposure and race and ethnicity. *J Perinatol* 2022; **42**(10): 1346-52.

Gemmill A, Casey JA, Catalano R, Karasek D, Margerison CE, Bruckner T. Changes in preterm birth and caesarean deliveries in the United States during the SARS-CoV-2 pandemic. *Paediatr Perinat Epidemiol* 2022; **36**(4): 485-9.

McDonnell S, Lindow SW, Sloan J, McNamee E, O'Connell MP. Maternal and Neonatal Outcomes During the First Year of the Covid-19 Pandemic. *Ir Med J* 2022; **115**(7): 639.

Kern-Goldberger AR, Sheils NE, Ventura MEM, et al. Patterns of Prenatal Care Delivery and Obstetric Outcomes before and during the COVID-19 Pandemic. *Am J Perinatol* 2023; **40**(6): 582-8.

Aboulatta L, Kowalec K, Leong C, et al. Preterm birth and stillbirth rates associated with socioeconomic disparities during COVID-19 pandemic: a population-based cross-sectional study. *BMJ Paediatr Open* 2023; **7**(1).

DeBolt CA, Roig J, Spiera E, et al. The Impact of the COVID-19 Pandemic on Postpartum Readmission Rates at a Single Tertiary Care Center in New York City. *Am J Perinatol* 2022; **39**(11): 1145-50.

Hui L, Marzan MB, Potenza S, et al. Increase in preterm stillbirths in association with reduction in iatrogenic preterm births during COVID-19 lockdown in Australia: a multicenter cohort study. *Am J Obstet Gynecol* 2022; **227**(3): 491.e1-.e17.

Stansfield S, Rattan A, Mol BW, Rolnik DL, Malhotra A. Impact of the COVID-19 pandemic and multiple community lockdowns on total live birth rates and preterm births in Melbourne, Australia. *Aust N Z J Obstet Gynaecol* 2022; **62**(5): 786-9.

Rao MG, Toner LE, Stone J, et al. Pregnancy during a Pandemic: A Cohort Study Comparing Adverse Outcomes during and before the COVID-19 Pandemic. *Am J Perinatol* 2023; **40**(4): 445-52.

Vani K, Estrada Trejo F, Plagianos M, et al. Incidence and characteristics of stillbirths before and during the Coronavirus 2019 pandemic. *J Matern Fetal Neonatal Med* 2022; **35**(26): 10324-9.

Kołomańska-Bogucka D, Pławiak N, Mazur-Bialy AI. The Impact of the COVID-19 Pandemic on the Level of Physical Activity, Emotional State, and Health Habits of Women in Late Pregnancy and Early Puerperium. *Int J Environ Res Public Health* 2023; **20**(3).

Panzer A, Reed-Weston A, Friedman A, Goffman D, Wen T. Expedited postpartum discharge during the COVID-19 pandemic and acute postpartum care utilization. *J Matern Fetal Neonatal Med* 2022; **35**(25): 9585-92.

Haikin Herzberger E, Efros O, Herzberger S, Biron-Shental T, Shechter-Maor G. Differences in obstetric healthcare utilization and delivery complications before and after the COVID-19 pandemic - a retrospective study. *J Matern Fetal Neonatal Med* 2022; **35**(25): 7194-9.

Amadori R, Aquino CI, Colagiorgio S, Osella E, Surico D, Remorgida V. What may happen if you are pregnant during COVID-19 lockdown? A retrospective study about peripartum outcomes. *Minerva Obstet Gynecol* 2022; **74**(4): 319-24.

Fresson J, Bruckner TA, Le Ray C, et al. Decreases in preterm birth during the first COVID-19 lockdown in France by gestational age sub-groups and regional COVID-19 incidence. *Annals of Epidemiology* 2022; **72**: 74-81.

Dol J, Hughes B, Aston M, McMillan D, Murphy GT, Campbell-Yeo M. Impact of COVID-19 restrictions on the postpartum experience of women living in Eastern Canada during the early pandemic period: A cross-sectional study. *Journal of Nursing Scholarship* 2023; **55**(1): 178-86.

Clifton VL, Kumar S, Borg D, et al. Associations between COVID-19 lockdown and post-lockdown on the mental health of pregnant women, postpartum women and their partners from the Queensland family cohort prospective study. *BMC Pregnancy and Childbirth* 2022; **22**(1).

Magnus MC, Oakley LL, Hansen AV, et al. Fetal death after the introduction of COVID-19 mitigation measures in Sweden, Denmark and Norway: a registry-based study. *Scientific reports* 2022; **12**(1): 20625.

Mølholm Hansen B, Cueto H, Padkaer Petersen J, et al. Preterm birth rates were slightly lower in Denmark during the first year of the COVID-19 pandemic compared with the previous 4 years. *Acta Paediatrica, International Journal of Paediatrics* 2022; **111**(9): 1695-700.

Snelgrove JW, Simpson AN, Sutradhar R, Everett K, Liu N, Baxter NN. Preeclampsia and Severe Maternal Morbidity During the COVID-19 Pandemic: A Population-Based Cohort Study in Ontario, Canada. *Journal of Obstetrics and Gynaecology Canada* 2022; **44**(7): 777-84.

Salerno C, Donno V, Melis B, et al. Stillbirth occurrence during COVID-19 pandemic: A population-based prospective study. *Journal of Perinatal Medicine* 2022; **50**(6): 653-9.

Waschmann M, Rosen K, Gievers L, Hildebrand A, Laird A, Khaki S. Evaluating the Impact of the COVID-19 Pandemic on Postpartum Depression. *Journal of Women's Health* 2022; **31**(6): 772-8.

Morgan AS, Bradford C, Farrow H, Draper ES, Harrison C. Effects of the SARS-CoV-2 pandemic on perinatal activity in Yorkshire and the Humber region during 2020: an interrupted time series analysis. *Archives of Disease in Childhood: Fetal and Neonatal Edition* 2022; **107**(6): F624-F9.

Jones H, Seaborne M, Cowley L, et al. Population birth outcomes in 2020 and experiences of expectant mothers during the COVID-19 pandemic: A ‘born in Wales’ mixed methods study using routine data. *PLoS ONE* 2022; **17**(5 May).

Simon E, Cottenet J, Mariet A, et al. Impact of the COVID-19 pandemic on preterm birth and stillbirth: A nationwide, population-based retrospective cohort study. *Gynecologie Obstetrique Fertilite et Senologie* 2022; **50**(5): 442-3.

Melov SJ, Elhindi J, McGee T, et al. INVESTIGATING PERINATAL OUTCOMES DURING THE LOW PREVALENCE FIRST YEAR OF COVID-19 IN A MULTIETHNIC AUSTRALIAN POPULATION. *Journal of Paediatrics and Child Health* 2022; **58**(SUPPL 2): 15-6.

Quibel T, Winer N, Bussières L, et al. Impact of COVID-19-Related Lockdown on Delivery and Perinatal Outcomes: A Retrospective Cohort Study. *Journal of Clinical Medicine* 2022; **11**(3).

Almeida AC, Granado MC, Sousa P, Vieira MJ. COVID-19 pandemic lockdown effect on neonatal hospital admissions from the community. *Journal of Neonatal-Perinatal Medicine* 2022; **15**(3): 583-8.

Handley MSaKGaJYLaELaHHBaKDaJGaJFCaSC. Coronavirus Disease 2019 (COVID-19) Pandemic and Pregnancy Outcomes in a U.S. Population. *Obstetrics and Gynecology* 2021; **138**: 542-51.

Studies excluded at full text screening (no pre-covid pandemic comparison group)

The following studies were excluded at the full text screening as they presented no data from a pre-covid lockdowns and pandemic comparison group.

de Arriba-García M, Diaz-Martinez A, Monfort-Ortiz R, et al. GESTACOVID project: psychological and perinatal effects in Spanish pregnant women subjected to confinement due to the COVID-19 pandemic. *J Matern Fetal Neonatal Med* 2022; **35**(25): 5665-71.

Dib S, Rougeaux E, Vázquez A, Wells J, Fewtrell M. The impact of the COVID-19 lockdown on maternal mental health and coping in the UK: Data from the COVID-19 New Mum Study. *medRxiv* 2020: 2020.08.04.20168039.

Burhamah W, AlKhayyat A, Oroszlányová M, et al. The psychological burden of the COVID-19 pandemic and associated lockdown measures: Experience from 4000 participants. *Journal of Affective Disorders* 2020; **277**: 977-85.

Bradfield Z, Wynter K, Hauck Y, et al. Experiences of receiving and providing maternity care during the COVID-19 Pandemic in Australia: a five-cohort cross-sectional comparison. *medRxiv* 2020: 2020.09.22.20199331.

Bertholdt C, Epstein J, Alleyrat C, et al. Comparative evaluation of the impact of the COVID-19 lockdown on perinatal experience: A prospective multicentre study. *Bjog-an International Journal of Obstetrics and Gynaecology*.

Aydin E, Glasgow KA, Weiss SM, et al. Giving birth in a Pandemic: Women's Birth Experiences in England during COVID-19. *medRxiv* 2021: 2021.07.05.21260022.

Vitale E. How the Italian women perceived distress from their puerperal conditions during the COVID-19 outbreak. *Journal of Psychopathology* 2021; **27**(3): 135-9.

Masters GA, Asipenko E, Bergman AL, et al. Impact of the COVID-19 pandemic on mental health, access to care, and health disparities in the perinatal period. *Journal of Psychiatric Research* 2021; **137**: 126-30.

Liu J, Hung P, Alberg AJ, et al. Mental health among pregnant women with COVID-19–related stressors and worries in the United States. *Birth* 2021; **48**(4): 470-9.

Liu CH, Koire A, Erdei C, Mittal L. Unexpected changes in birth experiences during the COVID-19 pandemic: Implications for maternal mental health. *Archives of Gynecology and Obstetrics* 2021.

Ceulemans M, Foulon V, Ngo E, et al. Mental health status of pregnant and breastfeeding women during the COVID-19 pandemic—A multinational cross-sectional study. *Acta Obstetricia et Gynecologica Scandinavica* 2021; **100**(7): 1219-29.

Bell AJ, Scott KA, Jelliffe-Pawlowski L. Examination of Stress and Anxiety in Pregnancy during COVID-19 Using an Anti-racist Praxis: Insights from the HOPE COVID-19 Study. *American Journal of Physical Anthropology* 2021; **174**(SUPPL 71): 8-9.

Matsushima M, Horiguchi H. The COVID-19 Pandemic and Mental Well-Being of Pregnant Women in Japan: Need for Economic and Social Policy Interventions. *Disaster Med Public Health Prep* 2020: 1-6.

Malarkiewicz P, Maksymowicz S, Libura M. Mother's Loneliness: Involuntary Separation of Pregnant Women in Maternity Care Settings and Its Effects on The Experience of Mothers during the COVID-19 Pandemic. *International Journal of Environmental Research and Public Health* 2022; **19**(9).

Liu CH, Hyun S, Mittal L, Erdei C. Psychological risks to mother–infant bonding during the COVID-19 pandemic. *Pediatric Research* 2022; **91**(4): 853-61.

Lequertier B, McLean MA, Kildea S, et al. Perinatal Depression in Australian Women during the COVID-19 Pandemic: The Birth in the Time of COVID-19 (BITTOC) Study. *International Journal of Environmental Research and Public Health* 2022; **19**(9).

Jurgiel J, Graniak A, Jozwik K, Pomorski M. Pregnancy and childbirth during the coronavirus pandemic. The cross-sectional study of 1321 participants in Poland. *Ginekologia polska* 2022.

Janik K, Cwalina U, Iwanowicz-Palus G, Cybulski M. An Assessment of the Level of COVID-19 Anxiety among Pregnant Women in Poland: A Cross-Sectional Study. *J Clin Med* 2021; **10**(24).

Farewell CV, Jewell J, Walls J, Leiferman JA. A Mixed-Methods Pilot Study of Perinatal Risk and Resilience During COVID-19. *J Prim Care Community Health* 2020; **11**: 2150132720944074.

Chaves C, Marchena C, Palacios B, Salgado A, Duque A. Effects of the COVID-19 pandemic on perinatal mental health in Spain: Positive and negative outcomes. *Women and Birth* 2022; **35**(3): 254-61.

Bérard A, Gorgui J, Tchuente V, et al. The COVID-19 Pandemic Impacted Maternal Mental Health Differently Depending on Pregnancy Status and Trimester of Gestation. *International Journal of Environmental Research and Public Health* 2022; **19**(5).

Asai K, Wakashima K, Toda S, Koiwa K. Fear of novel coronavirus disease (COVID-19) among pregnant and infertile women in Japan. *J Affect Disord Rep* 2021; **4**: 100104.

Molgora S, Accordini M. Motherhood in the Time of Coronavirus: The Impact of the Pandemic Emergency on Expectant and Postpartum Women's Psychological Well-Being. *Front Psychol* 2020; **11**: 567155.

Mikolajkow A, Małyszczak K. Stress level and general mental state in Polish pregnant women during COVID-19 pandemic. *J Reprod Infant Psychol* 2021: 1-18.

Ionio C, Gallese M, Fenaroli V, et al. COVID-19: what about pregnant women during first lockdown in Italy? *J Reprod Infant Psychol* 2021: 1-13.

Guo J, De Carli P, Lodder P, Bakermans-Kranenburg MJ, Riem MME. Maternal mental health during the COVID-19 lockdown in China, Italy, and the Netherlands: a cross-validation study. *Psychol Med* 2021: 1-11.

Groulx T, Bagshawe M, Giesbrecht G, Tomfohr-Madsen L, Hetherington E, Lebel CA. Prenatal Care Disruptions and Associations With Maternal Mental Health During the COVID-19 Pandemic. *Front Glob Womens Health* 2021; **2**: 648428.

Dagklis T, Tsakiridis I, Mamopoulos A, Athanasiadis A, Pearson R, Papazisis G. Impact of the COVID-19 lockdown on antenatal mental health in Greece. *Psychiatry Clin Neurosci* 2020; **74**(11): 616-7.

Colli C, Penengo C, Garzitto M, et al. Prenatal Stress and Psychiatric Symptoms During Early Phases of the COVID-19 Pandemic in Italy. *Int J Womens Health* 2021; **13**: 653-62.

Christie HE, Beetham K, Stratton E, Francois ME. "Worn-out but happy": Postpartum Women's Mental Health and Well-Being During COVID-19 Restrictions in Australia. *Front Glob Womens Health* 2021; **2**: 793602.

Buonsenso D, Malorni W, Turriziani Colonna A, et al. Psychological Impact of the COVID-19 Pandemic on Pregnant Women. *Front Pediatr* 2022; **10**: 790518.

Basu A, Kim HH, Basaldua R, et al. A cross-national study of factors associated with women's perinatal mental health and wellbeing during the COVID-19 pandemic. *PLoS One* 2021; **16**(4): e0249780.

Baran J, Leszczak J, Baran R, et al. Prenatal and Postnatal Anxiety and Depression in Mothers during the COVID-19 Pandemic. *J Clin Med* 2021; **10**(14).

Yirmiya K, Yakirevich-Amir N, Preis H, Lotan A, Atzil S, Reuveni I. Women's Depressive Symptoms during the COVID-19 Pandemic: The Role of Pregnancy. *Int J Environ Res Public Health* 2021; **18**(8).

Mollard E, Kupzyk K, Moore T. Postpartum stress and protective factors in women who gave birth in the United States during the COVID-19 pandemic. *Womens Health (Lond)* 2021; **17**: 17455065211042190.

Meraya AM, Syed MH, Yasmeen A, et al. COVID-19 related psychological distress and fears among mothers and pregnant women in Saudi Arabia. *PLoS One* 2021; **16**(8): e0256597.

Liu CH, Erdei C, Mittal L. Risk factors for depression, anxiety, and PTSD symptoms in perinatal women during the COVID-19 Pandemic. *Psychiatry Res* 2021; **295**: 113552.

Lega I, Bramante A, Lauria L, et al. The Psychological Impact of COVID-19 among Women Accessing Family Care Centers during Pregnancy and the Postnatal Period in Italy. *Int J Environ Res Public Health* 2022; **19**(4).

Iwanowicz-Palus G, Mróz M, Korda A, Marcewicz A, Palus A. Perinatal Anxiety among Women during the COVID-19 Pandemic-A Cross-Sectional Study. *Int J Environ Res Public Health* 2022; **19**(5).

Alaya F, Worrall AP, O'Toole F, Doyle J, Duffy RM, Geary MP. Health-related quality of life and quality of care in pregnant and postnatal women during the coronavirus disease 2019 pandemic: A cohort study. *Int J Gynaecol Obstet* 2021; **154**(1): 100-5.

Mollard E, Kupzyk K. Birth Satisfaction During the Early Months of the COVID-19 Pandemic in the United States. *MCN Am J Matern Child Nurs* 2022; **47**(1): 6-12.

Lebel C, MacKinnon A, Bagshawe M, Tomfohr-Madsen L, Giesbrecht G. Elevated depression and anxiety symptoms among pregnant individuals during the COVID-19 pandemic. *J Affect Disord* 2020; **277**: 5-13.

Gur RE, White LK, Waller R, et al. The Disproportionate Burden of the COVID-19 Pandemic Among Pregnant Black Women. *Psychiatry Res* 2020; **293**: 113475.

Fernandes J, Tavares I, Bem-Haja P, Barros T, Carrito ML. A Longitudinal Study on Maternal Depressive Symptoms During the COVID-19 Pandemic: The Role of Strict Lockdown Measures and Social Support. *Int J Public Health* 2022; **67**: 1604608.

Bin-Nun A, Palmor-Haspal S, Mimouni FB, Kasirer Y, Hammerman C, Tuval-Moshiach R. Infant delivery and maternal stress during the COVID-19 pandemic: a comparison of the well-baby versus neonatal intensive care environments. *J Perinatol* 2021; **41**(11): 2614-20.

Barbosa-Leiker C, Smith CL, Crespi EJ, et al. Stressors, coping, and resources needed during the COVID-19 pandemic in a sample of perinatal women. *BMC Pregnancy Childbirth* 2021; **21**(1): 171.

Werchan DM, Hendrix CL, Ablow JC, et al. Behavioral coping phenotypes and associated psychosocial outcomes of pregnant and postpartum women during the COVID-19 pandemic. *Sci Rep* 2022; **12**(1): 1209.

Anderson MR, Salisbury AL, Uebelacker LA, Abrantes AM, Battle CL. Stress, coping and silver linings: How depressed perinatal women experienced the COVID-19 pandemic. *J Affect Disord* 2022; **298**(Pt A): 329-36.

Hviid A, Laksafoss A, Hedley P, et al. Assessment of Seasonality and Extremely Preterm Birth in Denmark. *JAMA Netw Open* 2022; **5**(2): e2145800.

Goyal D, Beck CT, Webb R, Ayers S. Postpartum Depressive Symptoms and Experiences During COVID-19. *MCN Am J Matern Child Nurs* 2022; **47**(2): 77-84.

Gluska H, Shiffman N, Mayer Y, et al. Maternal fear of COVID-19 and prevalence of postnatal depression symptoms, risk and protective factors. *J Psychiatr Res* 2022; **148**: 214-9.

Filippetti ML, Clarke ADF, Rigato S. The mental health crisis of expectant women in the UK: effects of the COVID-19 pandemic on prenatal mental health, antenatal attachment and social support. *BMC Pregnancy Childbirth* 2022; **22**(1): 68.

Fallon V, Davies SM, Silverio SA, Jackson L, De Pascalis L, Harrold JA. Psychosocial experiences of postnatal women during the COVID-19 pandemic. A UK-wide study of prevalence rates and risk factors for clinically relevant depression and anxiety. *J Psychiatr Res* 2021; **136**: 157-66.

Eberhard-Gran M, Engelsen LY, Al-Zirqi I, Vangen S. Depressive symptoms and experiences of birthing mothers during COVID-19 pandemic. *Tidsskr Nor Laegeforen* 2022; **142**(3).

Brik M, Sandonis MA, Fernández S, et al. Psychological impact and social support in pregnant women during lockdown due to SARS-CoV2 pandemic: A cohort study. *Acta Obstet Gynecol Scand* 2021; **100**(6): 1026-33.

Bayrampour H, Tamana SK, Boutin A. Pregnant people's responses to the COVID-19 pandemic: a mixed-methods, descriptive study. *CMAJ Open* 2022; **10**(1): E146-e54.

Morniroli D, Consales A, Colombo L, et al. Exploring the Impact of Restricted Partners' Visiting Policies on Non-Infected Mothers' Mental Health and Breastfeeding Rates during the COVID-19 Pandemic. *Int J Environ Res Public Health* 2021; **18**(12).

Kinser PA, Jallo N, Amstadter AB, et al. Depression, Anxiety, Resilience, and Coping: The Experience of Pregnant and New Mothers During the First Few Months of the COVID-19 Pandemic. *J Womens Health (Larchmt)* 2021; **30**(5): 654-64.

Breman RB, Neerland C, Bradley D, Burgess A, Barr E, Burcher P. Giving birth during the COVID-19 pandemic, perspectives from a sample of the United States birthing persons during the first wave: March-June 2020. *Birth* 2021; **48**(4): 524-33.

Esteban-Gonzalo S, Caballero-Galilea M, González-Pascual JL, Álvaro-Navidad M, Esteban-Gonzalo L. Anxiety and Worries among Pregnant Women during the COVID-19 Pandemic: A Multilevel Analysis. *Int J Environ Res Public Health* 2021; **18**(13).

Adhikari EH, Moreno W, Zofkie AC, et al. Pregnancy Outcomes Among Women With and Without Severe Acute Respiratory Syndrome Coronavirus 2 Infection. *JAMA Netw Open* 2020; **3**(11): e2029256.

Mappa I, Distefano FA, Rizzo G. Effects of coronavirus 19 pandemic on maternal anxiety during pregnancy: a prospectic observational study. *J Perinat Med* 2020; **48**(6): 545-50.

Farrell T, Reagu S, Mohan S, et al. The impact of the COVID-19 pandemic on the perinatal mental health of women. *J Perinat Med* 2020; **48**(9): 971-6.

Cameron EE, Joyce KM, Delaquis CP, Reynolds K, Protudjer JLP, Roos LE. Maternal psychological distress & mental health service use during the COVID-19 pandemic. *J Affect Disord* 2020; **276**: 765-74.

Strahm AM, Christian LM, Hilmert CJ. Gestational length during COVID-19. *Psychosomatic Medicine* 2021; **83**(7): A39.

Ahlers-Schmidt CR, Hervey AM, Neil T, Kuhlmann S, Kuhlmann Z. Concerns of women regarding pregnancy and childbirth during the COVID-19 pandemic. *Patient Educ Couns* 2020; **103**(12): 2578-82.

Aytha SP, Regan A, Grinshteyn E, Nosek M, Gu NY. COVID-19 Concerns Experienced by Pregnant and Postpartum Women and Their Influence on Health-Related Quality-of-Life. *Value in Health* 2022; **25**(1): S13-S4.

Wyszynski DF, Hernandez-Diaz S, Gordon-Dseagu V, Ramiro N, Koenen KC. Stress levels among an international sample of pregnant and postpartum women during the COVID-19 pandemic. *Journal of Maternal-Fetal and Neonatal Medicine* 2021.

Gonzalez-Garcia V, Exertier M, Denis A. Anxiety, post-traumatic stress symptoms, and emotion regulation: A longitudinal study of pregnant women having given birth during the COVID-19 pandemic. *European Journal of Trauma & Dissociation* 2021; **5**(2).

Meaney S, Leitao S, Olander EK, Pope J, Matvienko-Sikar K. The impact of COVID-19 on pregnant womens' experiences and perceptions of antenatal maternity care, social support, and stress-reduction strategies. *Women and Birth* 2021.

Lubián López DM, Butrón Hinojo CA, Arjona Bernal JE, et al. Resilience and psychological distress in pregnant women during quarantine due to the COVID-19 outbreak in Spain: a multicentre cross-sectional online survey. *J Psychosom Obstet Gynaecol* 2021; **42**(2): 115-22.

Lambelet V, Ceulemans M, Nordeng H, et al. Impact of the COVID-19 pandemic on Swiss pregnant and breastfeeding women - a cross-sectional study covering the first pandemic wave. *Swiss Med Wkly* 2021; **151**: w30009.

Kelley EL, Zhao L. ASSOCIATIONS BETWEEN COVID-19 PANDEMIC CONDITIONS AND MATERNAL MENTAL HEALTH AND MATERNAL-INFANT BONDING. *Fertility and Sterility* 2021; **116**(3): e363-e4.

Janevic T, Lieb W, Ibroci E, et al. The influence of structural racism, pandemic stress, and SARS-CoV-2 infection during pregnancy with adverse birth outcomes. *Am J Obstet Gynecol MFM* 2022: 100649.

Gluska H, Shiffman N, Mayer Y, et al. Longitudinal study of Postpartum Depression in COVID-19 Era: Risk and Protective Factors. *American Journal of Obstetrics and Gynecology* 2022; **226**(1): S178.

de Arriba-García M, Diaz-Martinez A, Monfort-Ortiz R, et al. GESTACOVID project: psychological and perinatal effects in Spanish pregnant women subjected to confinement due to the COVID-19 pandemic. *J Matern Fetal Neonatal Med* 2021: 1-7.

Buthmann J, Coury S, Gotlib I. P270. Coping During the COVID-19 Pandemic: Maternal Mental Health and Infant Temperament. *Biological Psychiatry* 2022; **91**(9): S196-S7.

Ben-Yaakov O, Taubman-Ben-Ari O. COVID-19-related anxieties in first-time mothers during the first wave of the COVID-19 pandemic and after 6 months: A descriptive study. *Journal of pediatric nursing* 2022.

Sturrock S, Turner K, Lee-Wo C, et al. The COVID19 pandemic has changed women's experiences of pregnancy in the UK. *medRxiv* 2021: 2021.11.05.21265698.

Fransson E, Karalexi M, Kimmel M, et al. Mental health among pregnant women during the pandemic in Sweden– a mixed methods approach using data from the Mom2B mobile application for research. *medRxiv* 2020: 2020.12.18.20248466.

Rhodes A, Kheireddine S, Smith AD. Experiences, Attitudes, and Needs of Users of a Pregnancy and Parenting App (Baby Buddy) During the COVID-19 Pandemic: Mixed Methods Study. *Jmir Mhealth and Uhealth* 2020; **8**(12).

Morris AR, Herzig SE, Orozco M, et al. Delivering Alone in a Pandemic: Anticipated Changes to Partner Presence at Birth Are Associated With Prenatal Distress. *Families Systems & Health* 2022; **40**(1): 126-31.

McMillan IF, Armstrong LM, Langhinrichsen-Rohling J. Transitioning to Parenthood During the Pandemic: COVID-19 Related Stressors and First-Time Expectant Mothers' Mental Health. *Couple and Family Psychology-Research and Practice* 2021; **10**(3): 179-89.

Taubman–Ben-Ari O, Chasson M, Abu Sharkia S, Weiss E. Distress and anxiety associated with COVID-19 among Jewish and Arab pregnant women in Israel. *Journal of Reproductive and Infant Psychology* 2020; **38**(3): 340-8.

Mazza C, Di Giandomenico S, Ricci E. Psychological distress among Italian parents during COVID-19 lockdown: Risk and protective factors. *Journal of Psychopathology* 2021; **27**(SUPPL 1): 12.

Wah Hui P, Seto MTY, Wang Cheung K. Postnatal depression scores and maternal characteristics during COVID-19 pandemic. *BJOG: An International Journal of Obstetrics and Gynaecology* 2021; **128**(SUPPL 2): 203-4.

Samantha Sze Minn Goh M, Chun Wei Lim C, Chua K, et al. Knowledge, attitudes and expectations of perinatal care during the COVID-19 pandemic. *BJOG: An International Journal of Obstetrics and Gynaecology* 2021; **128**(SUPPL 2): 200-1.

Regan A, Aytha Swathi P, Nosek M, Gu NY. Impact of COVID-19 on the Health-Related Quality-of-Life of Pregnant and Postpartum Persons. *Value in Health* 2022; **25**(1): S6.

Pope J, Olander EK, Leitao S, Meaney S, Matvienko-Sikar K. Prenatal stress, health, and health behaviours during the COVID-19 pandemic: An international survey. *Women and Birth* 2022; **35**(3): 272-9.

Phipps J, Whipps M, D'Souza I, Lee K, Simmons LA. Pregnant in a pandemic: Mental well-being and associated health behaviors among California pregnant people during COVID-19. *Paediatric and Perinatal Epidemiology* 2021; **35**(SUPPL 2): 27.

Okezie C, Adeniji B, Monteitg C, Ahmed S. An insight into postnatal maternal health at the peak of Covid pandemic. *BJOG: An International Journal of Obstetrics and Gynaecology* 2021; **128**(SUPPL 2): 202.

Njoroge WFM, White LK, Waller R, et al. Association of COVID-19 and Endemic Systemic Racism with Postpartum Anxiety and Depression among Black Birthing Individuals. *JAMA Psychiatry* 2022.

Morris JR, Jaswa EG, Kaing A, et al. ANXIETY DURING THE COVID-19 PANDEMIC: PRELIMINARY FINDINGS FROM THE UCSF ASPIRE STUDY. *Fertility and Sterility* 2021; **116**(3): e7-e8.

Kolker S, Biringer A, Carroll J, Bytautas J, Blumenfeld H. The psychosocial impact of COVID-19 on pregnant and postpartum individuals: a mixed methods exploration. *Journal of Obstetrics and Gynaecology Canada* 2021; **43**(5): 659-60.

Ali H, Choudhary R, Deodhar P. Trends in elective gynecological surgeries at a teaching institute during Covid-19 era. *BJOG: An International Journal of Obstetrics and Gynaecology* 2021; **128**(SUPPL 2): 196.

Tsakiridis I, Dagklis T, Mamopoulos A, Athanasiadis A, Pearson R, Papazisis G. Antenatal depression and anxiety during the COVID-19 pandemic: a cross-sectional study in pregnant women from routine health care contact in Greece. *J Perinat Med* 2021; **49**(6): 656-63.

Tavares IM, Fernandes J, Moura CV, Nobre PJ, Carrito ML. Adapting to Uncertainty: A Mixed-Method Study on the Effects of the COVID-19 Pandemic on Expectant and Postpartum Women and Men. *Front Psychol* 2021; **12**: 688340.

Spinola O, Liotti M, Speranza AM, Tambelli R. Effects of COVID-19 Epidemic Lockdown on Postpartum Depressive Symptoms in a Sample of Italian Mothers. *Front Psychiatry* 2020; **11**: 589916.

Smith CL, Waters SF, Spellacy D, et al. Substance use and mental health in pregnant women during the COVID-19 pandemic. *J Reprod Infant Psychol* 2021: 1-14.

Preis H, Mahaffey B, Heiselman C, Lobel M. Vulnerability and resilience to pandemic-related stress among U.S. women pregnant at the start of the COVID-19 pandemic. *Soc Sci Med* 2020; **266**: 113348.

Polloni L, Cavallin F, Lolli E, et al. Psychological Wellbeing of Parents with Infants Admitted to the Neonatal Intensive Care Unit during SARS-CoV-2 Pandemic. *Children (Basel)* 2021; **8**(9).

Ng QJ, Koh KM, Tagore S, Mathur M. Perception and Feelings of Antenatal Women during COVID-19 Pandemic: A Cross-Sectional Survey. *Ann Acad Med Singap* 2020; **49**(8): 543-52.

Motrico E, Domínguez-Salas S, Rodríguez-Domínguez C, Gómez-Gómez I, Rodríguez-Muñoz MF, Gómez-Baya D. The Impact of the COVID-19 Pandemic on Perinatal Depression and Anxiety: A Large Cross-sectional Study in Spain. *Psicothema* 2022; **34**(2): 200-8.

Morris JR, Jaswa E, Kaing A, et al. Early pregnancy anxiety during the COVID-19 pandemic: preliminary findings from the UCSF ASPIRE study. *BMC Pregnancy Childbirth* 2022; **22**(1): 272.

He D, Ren J, Luo B, et al. Women's Psychological Health, Family Function, and Social Support During Their Third Trimester of Pregnancy Within the COVID-19 Epidemic: A Cross-sectional Survey. *Disaster Med Public Health Prep* 2021: 1-5.

Terada S, Kinjo K, Fukuda Y. The relationship between postpartum depression and social support during the COVID-19 pandemic: A cross-sectional study. *J Obstet Gynaecol Res* 2021; **47**(10): 3524-31.

Stampini V, Monzani A, Caristia S, et al. The perception of Italian pregnant women and new mothers about their psychological wellbeing, lifestyle, delivery, and neonatal management experience during the COVID-19 pandemic lockdown: a web-based survey. *BMC Pregnancy Childbirth* 2021; **21**(1): 473.

Sanders J, Blaylock R. "Anxious and traumatised": Users' experiences of maternity care in the UK during the COVID-19 pandemic. *Midwifery* 2021; **102**: 103069.

Saleh L, Canclini S, Greer K, et al. Mothers' Experiences of Pregnancy, Labor and Birth, and Postpartum During COVID-19 in the United States: Preliminary Results of a Mixed-Methods Study. *J Perinat Neonatal Nurs* 2022; **36**(1): 55-67.

Preis H, Mahaffey B, Pati S, Heiselman C, Lobel M. Adverse Perinatal Outcomes Predicted by Prenatal Maternal Stress Among U.S. Women at the COVID-19 Pandemic Onset. *Ann Behav Med* 2021; **55**(3): 179-91.

Preis H, Mahaffey B, Lobel M. The role of pandemic-related pregnancy stress in preference for community birth during the beginning of the COVID-19 pandemic in the United States. *Birth* 2021; **48**(2): 242-50.

Overbeck G, Rasmussen IS, Siersma V, Kragstrup J, Ertmann RK, Wilson P. Mental well-being during stages of COVID-19 lockdown among pregnant women and new mothers. *BMC Pregnancy Childbirth* 2022; **22**(1): 92.

Ostacoli L, Cosma S, Bevilacqua F, et al. Psychosocial factors associated with postpartum psychological distress during the Covid-19 pandemic: a cross-sectional study. *BMC Pregnancy Childbirth* 2020; **20**(1): 703.

Obata S, Miyagi E, Haruyama Y, et al. Psychological stress among pregnant and puerperal women in Japan during the coronavirus disease 2019 pandemic. *J Obstet Gynaecol Res* 2021; **47**(9): 2990-3000.

Moyer CA, Compton SD, Kaselitz E, Muzik M. Pregnancy-related anxiety during COVID-19: a nationwide survey of 2740 pregnant women. *Arch Womens Ment Health* 2020; **23**(6): 757-65.

Kornfield SL, White LK, Waller R, et al. Risk And Resilience Factors Influencing Postpartum Depression And Mother-Infant Bonding During COVID-19. *Health Aff (Millwood)* 2021; **40**(10): 1566-74.

Janjua NB, Mohamed AF, Birmani SA, Donnelly O, Syed AH, Essajee M. COVID-19 Pandemic and Maternal Perspectives. *Ir Med J* 2021; **114**(7): 411.

Studies excluded at full text screening (outcome wrong, not aligned with outcome definitions)

The following studies were excluded at the full text screening as the outcomes presented were not aligned with those being investigated in the study or did not all under the studies outcomes definitions.

Summers LP, K. Firth, C. The unexpected effects of the COVID-19 visiting restrictions on the postnatal journey: an indirect pressure on the neonatal unit resource. *Archives of Disease in Childhood* 2021; **106**(SUPPL 1): A63.

Sade SS, E. Wainstock, T. Hermon, N. Salem, S. Y. Kosef, T. Battat, T. L. Oron, S. Pariente, G. Risk for depressive symptoms among hospitalized women in high-risk pregnancy units during the covid-19 pandemic. *Journal of Clinical Medicine* 2020; **9**(8): 1-11.

Rotshenker-Olshinka KV-P, A. Steiner, N. Rubenfeld, E. Dahan, M. H. COVID-19 pandemic effect on early pregnancy: are miscarriage rates altered, in asymptomatic women? *Arch Gynecol Obstet* 2021; **303**(3): 839-45.

Rigamonti CB, P. Amatulli, A. Nettuno, A. Tamiazzo, S. Perego, S. Dattolo, C. Trussardi, E. Locatelli, A. COVID 19: Aspetti materni, fetali, neonatali e organizzativi Perinatal Depression Screening in ASST Brianza: Longitudinal analysis pre and during Covid 19 pandemia. *Journal of Maternal-Fetal and Neonatal Medicine* 2021; **34**(SUPPL 1): 92-3.

Racine NH, E. McArthur, B. A. McDonald, S. Edwards, S. Tough, S. Madigan, S. Maternal depressive and anxiety symptoms before and during the COVID-19 pandemic in Canada: a longitudinal analysis. *Lancet Psychiatry* 2021; **8**(5): 405-15.

McLaren RA, Jr. Trejo, F. E. Blitz, M. J. Bianco, A. Limaye, M. Brustman, L. Bernstein, P. S. Roman, A. S. Stone, J. Minkoff, H. COVID-related "lockdowns" and birth rates in New York. *Am J Obstet Gynecol MFM* 2021; **3**(6): 100476.

McClurg ABS, R. G. Moore, K. J. Louie, M. Patient Characteristics Associated with Delays to Benign Gynecologic Surgery: Impact of the COVID-19 Pandemic. *Journal of Minimally Invasive Gynecology* 2021; **28**(11): S129.

Khalil AvD, P. Kalafat, E. Sebghati, M. Ladhani, S. Ugwumadu, A. Draycott, T. O'Brien, P. Magee, L. Change in obstetric attendance and activities during the COVID-19 pandemic. *Lancet Infect Dis* 2021; **21**(5): e115.

Janevic TM, S. Nowlin, S. McCarthy, K. Bergink, V. Stone, J. Dias, J. Wu, S. Howell, E. A. Pandemic Birthing: Childbirth Satisfaction, Perceived Health Care Bias, and Postpartum Health During the COVID-19 Pandemic. *Maternal and child health journal* 2021; **25**(6): 860-9.

Inversetti AF, S. Nespoli, A. Antolini, L. Mussi, S. Ferrari, D. Vicini, D. Volontè, S. Locatelli, A. Birth satisfaction during COVID-19 pandemia compared to pre-covid era. *Journal of Maternal-Fetal and Neonatal Medicine* 2021; **34**(SUPPL 1): 10-1.

Indrio FS, A. Amato, O. Bartoli, F. Capasso, L. Corvaglia, L. Maffei, G. Mosca, F. Pettoello Mantovani, M. Raimondi, F. Rinaldi, M. Roggero, P. Aceti, A. COVID-19 pandemic in the neonatal intensive care unit: any effect on late-onset sepsis and necrotizing enterocolitis? *Eur J Pediatr* 2022; **181**(2): 853-7.

Combs ALW, L. VanDillen, M. Mussarat, N. Tate, D. 759Fetal demise in the time of a pandemic: is there a correlation? *American Journal of Obstetrics and Gynecology* 2021; **224**(2): S475.

Brown NM, A. R. Kearns, R. Litchfield, K. O.9 Going viral: enhanced recovery after planned caesarean delivery during a global pandemic. *International Journal of Obstetric Anesthesia* 2021; **46**.

Breinig SM, G. Brossier, D. Amadieu, R. Claudet, I. Javouhey, E. Angoulvant, F. Arnaud, C. Impact of the French National Lockdown on Admissions to 14 Pediatric Intensive Care Units During the 2020 COVID-19 Pandemic–A Retrospective Multicenter Study. *Frontiers in Pediatrics* 2021; **9**.

Bishop CM, Y. Letchworth, P. Pregnancy in the time of COVID-19: A retrospective review comparing cases of in-utero death and placental abruption in 2020 to those in 2019. *BJOG: An International Journal of Obstetrics and Gynaecology* 2021; **128**(SUPPL 1): 133-4.

Baker MVP, L. Adam, R. A. Same day discharge after vaginal hysterectomy in the covid-19 pandemic. *American Journal of Obstetrics and Gynecology* 2022; **226**(3): S1319.

Asali AF-G, S. Hasky, N. Elbaz, M. Fishman, A. Ravid, D. Wiser, A. Biron-Shental, T. Berkovitz, A. Miller, N. Physiological and psychological stress responses to labor and delivery during COVID-19 pandemic: a cohort study. *Journal of Psychosomatic Obstetrics & Gynecology*.

Anderson TSS, J. P. Pinheiro, A. Li, S. Herzig, S. J. Hospitalizations for Emergent Medical, Surgical, and Obstetric Conditions in Boston During the COVID-19 Pandemic. *J Gen Intern Med* 2020; **35**(10): 3129-32.

Adams C. Pregnancy and birth in the United States during the COVID-19 pandemic: The views of doulas. *Birth* 2022; **49**(1): 116-22.

Abel MKA, M. X. Tierney, C. Weintraub, M. R. Avins, A. Zaritsky, E. Coronavirus Disease 2019 (COVID-19) and the Incidence of Obstetric and Gynecologic Emergency Department Visits in an Integrated Health Care System. *Obstet Gynecol* 2021; **137**(4): 581-3.

Abdoli A. The COVID-19 stress may influence on the sex ratio at birth. *Journal of Maternal-Fetal & Neonatal Medicine*.

Broberg L, Rom AL, de Wolff MG, et al. Psychological well-being and worries among pregnant women in the first trimester during the early phase of the COVID-19 pandemic in Denmark compared with a historical group: A hospital-based cross-sectional study. *Acta Obstet Gynecol Scand* 2022; **101**(2): 232-40.

Twanow JDE, McCabe C, Ream MA. The COVID-19 Pandemic and Pregnancy: Impact on Mothers and Newborns. *Seminars in Pediatric Neurology* 2022; **42**.

Karim F, Sheehan H, Ganapathy R. The impact of Covid-19 on gynaecological emergencies. *Journal of Obstetrics and Gynaecology* 2022; **42**(6): 2529-30.

Wilson CA, Gomez-Gomez I, Parsons J, et al. The Mental Health of Women with Gestational Diabetes During the COVID-19 Pandemic: An International Cross-Sectional Survey. *Journal of Womens Health* 2022; **31**(9): 1232-40.

Pilu EMaMFaVTaICaAPaSDaGSaG. When the going gets tough, the tough get larger: how is pandemic affecting fetal weight? *Minerva Obstetrics and Gynecology* 2023; **75**: 201-2.

Prasannan L, Gerber R, Gulersen M, Shan WW, Blitz MJ, Rochelson B. Obstetrical Outcomes After Implementation of Laborist Model During the COVID-19 Pandemic. *Journal of Patient Safety* 2022; **18**(8): E1243-E6.

Kołomańska-Bogucka D, Micek A, Mazur-Bialy AI. The COVID-19 Pandemic and Levels of Physical Activity in the Last Trimester, Life Satisfaction and Perceived Stress in Late Pregnancy and in the Early Puerperium. *Int J Environ Res Public Health* 2022; **19**(5).

Studies excluded at full text screening (population not high-income country)

The following studies were excluded at the full text screening as the population was not based in a high-income country as defined by the World Bank classifications.

Yakupova V, Suarez A, Kharchenko A. Birth experience, postpartum ptsd and depression before and during the pandemic of covid-19 in Russia. *International Journal of Environmental Research and Public Health* 2022; **19**(1).

Ho-Fung C, Andersson E, Hsuan-Ying H, Acharya G, Schwank S. Self-reported mental health status of pregnant women in Sweden during the COVID-19 pandemic: a cross-sectional survey. *BMC Pregnancy Childbirth* 2022; **22**(1): 260.

Cuestas E, Gómez-Flores ME, Charras MD, et al. Association between COVID-19 mandatory lockdown and decreased incidence of preterm births and neonatal mortality. *J Perinatol* 2021; **41**(10): 2566-9.

Xue R-HaLJaCLaLZ-ZaHZaHH-FaLX-H. Alternations of cesarean section rates in a non-infected population after the outbreak of COVID-19: a cross-sectional study. *Psychology Health \& Medicine*.

Shchegolev AI, Tumanova UN, Chausov AA, Shuvalova MP. STILLBIRTHS IN THE RUSSIAN FEDERATION IN 2020 (COVID-19 PANDEMIC YEAR). *Akusherstvo i Ginekologiya (Russian Federation)* 2022; **2022**(11): 131-40.

Studies excluded at full text screening (study type wrong)

The following studies were excluded at the full text screening stage as the study type was not eligible or the design meet the criteria outlined in the inclusion criteria. Many were reviews, qualitative studies, or meta-analysis.

Blebu BE, Kuppermann M, Coleman-Phox K, Karasek D, Lessard L, Chambers BD. A qualitative exploration of experiences accessing community and social services among pregnant low-income people of color during the COVID-19 pandemic. *Women's Health* 2023; **19**.

Lemmon ME, Chapman I, Malcolm W, et al. Beyond the First Wave: Consequences of COVID-19 on High-Risk Infants and Families. *American Journal of Perinatology* 2020; **37**(12): 1283-8.

Isaacs D. Reduced rates of premature births in Denmark during COVID-19 lockdown. *Journal of Paediatrics and Child Health* 2021; **57**(9): 1544-5.

Aydin E, Weiss SM, Glasgow KA, et al. The COVID in the Context of Pregnancy, Infancy and Parenting (CoCoPIP) Study: protocol for a longitudinal study of parental mental health, social interactions, physical growth, and cognitive development of infants during the pandemic. *medRxiv* 2021: 2021.05.22.21257649.

Green J, Petty J, Whiting L, Fowler C. Exploring modifiable risk-factors for premature birth in the context of COVID-19 mitigation measures: A discussion paper. *J Neonatal Nurs* 2021; **27**(3): 172-9.

Bagci S, Sabir H, Müller A, Reiter RJ. Effects of altered photoperiod due to COVID-19 lockdown on pregnant women and their fetuses. *Chronobiol Int* 2020; **37**(7): 961-73.

Kajdy A, Feduniw S, Ajdacka U, et al. Risk factors for anxiety and depression among pregnant women during the COVID-19 pandemic: A web-based cross-sectional survey. *Medicine (Baltimore)* 2020; **99**(30): e21279.

Dove-Medows E, Davis J, McCracken L, et al. A Mixed-Methods Study of Experiences during Pregnancy among Black Women during the COVID-19 Pandemic. *Journal of Perinatal and Neonatal Nursing* 2022; **36**(2): 161-72.

Ghazanfarpour M, Bahrami F, Rashidi Fakari F, et al. Prevalence of anxiety and depression among pregnant women during the COVID-19 pandemic: a meta-analysis. *Journal of Psychosomatic Obstetrics and Gynecology* 2022; **43**(3): 315-26.

Fritsch E. Impacts of the COVID-19 Pandemic on Pregnant and Postpartum Women: Where Is the Village? *Psychoanalytic Psychology* 2022.

M. Oberndorfer, P. M. Henery, R. Dundas, A. H. Leyland, S. Paranjothy, S. J. Stock, et al. Study protocol: examining the impacts of COVID-19 mitigation measures on pregnancy and birth outcomes in Scotland—a linked administrative data study. *BMJ Open* 2023; **13**.

Calvert C, Brockway MM, Zoega H, et al. Changes in preterm birth and stillbirth during COVID-19 lockdowns in 26 countries. *Nat Hum Behav* 2023; **7**(4): 529-44.

Supplementary Material 12: PRISMA 2020 Checklists

Section and Topic	Item #	Checklist item	Location where item is reported
**TITLE**			
Title	1	Identify the report as a systematic review.	Yes (title)
**ABSTRACT**			
Abstract	2	See the PRISMA 2020 for Abstracts checklist.	Completed (Supplementary Material 6 & below)
**INTRODUCTION**			
Rationale	3	Describe the rationale for the review in the context of existing knowledge.	Yes (Introduction)
Objectives	4	Provide an explicit statement of the objective(s) or question(s) the review addresses.	Yes (Introduction)
**METHODS**			
Eligibility criteria	5	Specify the inclusion and exclusion criteria for the review and how studies were grouped for the syntheses.	Yes (Methods)
Information sources	6	Specify all databases, registers, websites, organisations, reference lists and other sources searched or consulted to identify studies. Specify the date when each source was last searched or consulted.	Yes (Methods)
Search strategy	7	Present the full search strategies for all databases, registers and websites, including any filters and limits used.	Yes (Appendix B)
Selection process	8	Specify the methods used to decide whether a study met the inclusion criteria of the review, including how many reviewers screened each record and each report retrieved, whether they worked independently, and if applicable, details of automation tools used in the process.	Yes (Methods)
Data collection process	9	Specify the methods used to collect data from reports, including how many reviewers collected data from each report, whether they worked independently, any processes for obtaining or confirming data from study investigators, and if applicable, details of automation tools used in the process.	Yes (Methods)
Data items	10a	List and define all outcomes for which data were sought. Specify whether all results that were compatible with each outcome domain in each study were sought (e.g. for all measures, time points, analyses), and if not, the methods used to decide which results to collect.	Yes (Methods & Appendix A)
	10b	List and define all other variables for which data were sought (e.g. participant and intervention characteristics, funding sources). Describe any assumptions made about any missing or unclear information.	Yes (Methods)
Study risk of bias assessment	11	Specify the methods used to assess risk of bias in the included studies, including details of the tool(s) used, how many reviewers assessed each study and whether they worked	Yes (Methods)

Section and Topic	Item #	Checklist item	Location where item is reported
		independently, and if applicable, details of automation tools used in the process.	
Effect measures	12	Specify for each outcome the effect measure(s) (e.g. risk ratio, mean difference) used in the synthesis or presentation of results.	Yes (Methods)
Synthesis methods	13a	Describe the processes used to decide which studies were eligible for each synthesis (e.g. tabulating the study intervention characteristics and comparing against the planned groups for each synthesis (item #5)).	Yes (Methods)
	13b	Describe any methods required to prepare the data for presentation or synthesis, such as handling of missing summary statistics, or data conversions.	Yes (Methods)
	13c	Describe any methods used to tabulate or visually display results of individual studies and syntheses.	Yes (Methods)
	13d	Describe any methods used to synthesize results and provide a rationale for the choice(s). If meta-analysis was performed, describe the model(s), method(s) to identify the presence and extent of statistical heterogeneity, and software package(s) used.	Yes (Methods & Appendix C)
	13e	Describe any methods used to explore possible causes of heterogeneity among study results (e.g. subgroup analysis, meta-regression).	Yes (Methods)
	13f	Describe any sensitivity analyses conducted to assess robustness of the synthesized results.	Yes (Methods & Appendix C)
Reporting bias assessment	14	Describe any methods used to assess risk of bias due to missing results in a synthesis (arising from reporting biases).	Yes (Methods)
Certainty assessment	15	Describe any methods used to assess certainty (or confidence) in the body of evidence for an outcome.	Yes (Methods)
**RESULTS**			
Study selection	16a	Describe the results of the search and selection process, from the number of records identified in the search to the number of studies included in the review, ideally using a flow diagram.	Yes (Results & Extended data 1)
	16b	Cite studies that might appear to meet the inclusion criteria, but which were excluded, and explain why they were excluded.	Yes (Supplementary Material 3 & 4)
Study characteristics	17	Cite each included study and present its characteristics.	Yes (Appendix D)
Risk of bias in studies	18	Present assessments of risk of bias for each included study.	Yes (Appendix D)
Results of individual studies	19	For all outcomes, present, for each study: (a) summary statistics for each group (where appropriate) and (b) an effect estimate and its precision (e.g. confidence/credible interval), ideally using structured tables or plots.	Yes (Results & Extended Data 2 - 7)
Results of syntheses	20a	For each synthesis, briefly summarise the characteristics and risk of bias among contributing studies.	Yes (Appendix D)

Section and Topic	Item #	Checklist item	Location where item is reported
	20b	Present results of all statistical syntheses conducted. If meta-analysis was done, present for each the summary estimate and its precision (e.g. confidence/credible interval) and measures of statistical heterogeneity. If comparing groups, describe the direction of the effect.	Yes (Results & Extended Data 2 - 7)
	20c	Present results of all investigations of possible causes of heterogeneity among study results.	Yes (Results & Supplementary Material 1)
	20d	Present results of all sensitivity analyses conducted to assess the robustness of the synthesized results.	Yes (Results, Extended data 8, & Supplementary Material 2)
Reporting biases	21	Present assessments of risk of bias due to missing results (arising from reporting biases) for each synthesis assessed.	Yes (Results, Extended data 8, & Supplementary Material 2)
Certainty of evidence	22	Present assessments of certainty (or confidence) in the body of evidence for each outcome assessed.	Yes (Results, Extended data 8, & Supplementary Material 2 - 3)
**DISCUSSION**			
Discussion	23a	Provide a general interpretation of the results in the context of other evidence.	Yes (Discussion)
	23b	Discuss any limitations of the evidence included in the review.	Yes (Discussion)
	23c	Discuss any limitations of the review processes used.	Yes (Discussion)
	23d	Discuss implications of the results for practice, policy, and future research.	Yes (Discussion & Conclusion)
**OTHER INFORMATION**			
Registration and protocol	24a	Provide registration information for the review, including register name and registration number, or state that the review was not registered.	Yes (abstract)
	24b	Indicate where the review protocol can be accessed, or state that a protocol was not prepared.	Yes (abstract)
	24c	Describe and explain any amendments to information provided at registration or in the protocol.	Yes (response checklist)
Support	25	Describe sources of financial or non-financial support for the review, and the role of the funders or sponsors in the review.	Yes (methods)
Competing interests	26	Declare any competing interests of review authors.	Yes (competing interest statements)
Availability of data, code and other materials	27	Report which of the following are publicly available and where they can be found: template data collection forms; data extracted from included studies; data used for all analyses; analytic code; any other materials used in the review.	Template (Appendix D)<br>Data extracted (Appendix D, Results, Extended data 8, & Supplementary Material 2 - 3)<br>Analytic code (upon request)

PRISMA 2020 Abstract checklist

Section and Topic	Item #	Checklist item	Reported (Yes/No)
**TITLE**			
Title	1	Identify the report as a systematic review.	Yes
**BACKGROUND**			
Objectives	2	Provide an explicit statement of the main objective(s) or question(s) the review addresses.	Yes
**METHODS**			
Eligibility criteria	3	Specify the inclusion and exclusion criteria for the review.	Yes
Information sources	4	Specify the information sources (e.g. databases, registers) used to identify studies and the date when each was last searched.	Yes
Risk of bias	5	Specify the methods used to assess risk of bias in the included studies.	Yes
Synthesis of results	6	Specify the methods used to present and synthesise results.	Yes
**RESULTS**			
Included studies	7	Give the total number of included studies and participants and summarise relevant characteristics of studies.	Yes
Synthesis of results	8	Present results for main outcomes, preferably indicating the number of included studies and participants for each. If meta-analysis was done, report the summary estimate and confidence/credible interval. If comparing groups, indicate the direction of the effect (i.e. which group is favoured).	Yes
**DISCUSSION**			
Limitations of evidence	9	Provide a brief summary of the limitations of the evidence included in the review (e.g. study risk of bias, inconsistency and imprecision).	Yes
Interpretation	10	Provide a general interpretation of the results and important implications.	Yes
**OTHER**			
Funding	11	Specify the primary source of funding for the review.	Yes
Registration	12	Provide the register name and registration number.	Yes

Supplementary Material 13. List of Supplementary Figures & Captions

Supplementary Figure 1. *Forest plot of risk ratios of preterm birth over lockdown compared to pre-lockdown. Subgroup analyses stratified individual studies or studies' available data by continental region (Panel B), ethnicity group (Panel C), deprivation level (Panel D), and estimates adjusted for previous temporal trends in PTB (Panel E).*

Caption: Individual study risk ratios (RRs) are represented by black diamond symbols (♦) and grey boxes, exact values are defined in the column to the right of the forest plot. RRs were calculated by comparing the risk of PTB in the lockdown group with the risk of PTB in the pre-lockdown group. The number of PTBs and livebirths, listed in the second to fourth columns from the left, were used to calculate the risk of PTB in each period. The black horizontal lines running through each black diamond indicate the 95% confidence intervals of the individual study RR, the exact values of the upper and lower bounds of which are defined in the brackets alongside the exact values of the RR (column right of the forest plot), arrows at the end of these lines indicate that the upper or lower limits of the confidence interval are higher or lower than the values defined on the x-axis. The grey box surrounding each black diamond symbolizes the percentage weighting of each study in the meta-analysis, the column furthest to the right defines the exact values of the weight. The solid, vertical, black line in the centre of the plot indicates the line of no effect. A dark blue, unfilled, diamond indicates the pooled RR and 95% confidence interval of all included studies and the pooled RRs of subgroups, estimated by two sided random-effects meta-analysis. Adjustments were not made for multiple comparisons. The exact values of the pooled estimate and 95% confidence intervals are defined in the corresponding row in the column to the right of the forest plot. The red, dashed, vertical line indicates the pooled RR, enabling comparison to individual studies RRs. The total number of PTBs and livebirths per period and the total in each subgroup is defined in the final row of the second to fourth columns from the left, and where "total" is defined for each subgroup. The magnitude and significance of heterogeneity is listed in the bottom left corner for all included studies and below each subgroup. Note: $p<0.000$ should be interpreted as $p<0.001$.

(Supplementary Figure 1. Panel A.)

(Supplementary Figure 1. Panel B.)

(Supplementary Figure 1. Panel C.)

(Supplementary Figure 1. Panel D.)

(Supplementary Figure 1. Panel E.)

Supplementary Figure 2. *Forest plot of risk ratios of stillbirth over lockdown compared to pre-lockdown, subgroup analysis includes estimates adjusted for underlying temporal trends in stillbirth (Panel B).*

Caption: Individual study risk ratios (RRs) are represented by black diamond symbols (♦) and grey boxes, exact values are defined in the column to the right of the forest plot. RRs were calculated by comparing the risk of stillbirth in the lockdown group with the risk of stillbirth in the pre-lockdown group. The number of stillbirths and total births, listed in the second to fourth columns from the left, were used to calculate the risk of stillbirth in each period. The black horizontal lines running through each black diamond or grey box indicate the 95% confidence intervals of the individual study RR, the exact values of the upper and lower bounds of which are defined in the brackets alongside the exact values of the RR (column right of the forest plot), arrows at the end of these lines indicate that the upper or lower limits of the confidence interval are higher or lower than the values defined on the x-axis. The grey box surrounding each black diamond symbolizes the percentage weighting of each study in the meta-analysis, the column furthest to the right defines the exact values of the weight. The solid, vertical, black line in the centre of the plot indicates the line of no effect. A dark blue, unfilled, diamond indicates the pooled RR and 95% confidence interval, estimated by two sided random-effects meta-analysis. Adjustments were not made for multiple comparisons. The exact values of the pooled estimate and 95% confidence intervals are defined in the corresponding row in the column to the right of the forest plot. The red, dashed, vertical line indicates the pooled RR, enabling comparison to individual studies RRs. The total number of stillbirths and total births per period is defined in the final row of the second to fourth columns from the left. The magnitude and significance of heterogeneity is listed in the bottom left corner for all included studies.

(Supplementary Figure 2. Panel A.)

(Supplementary Figure 2. Panel B.)

Supplementary Figure 3. *Forest plot of risk ratios of adverse neonatal outcomes over lockdown compared to pre-lockdown, outcomes include low birthweight (Panel A), neonatal mortality (Panel B), and neonatal admissions (Panel C).*

Caption: Individual study risk ratios (RRs) are represented by black diamond symbols (♦), exact values are defined in the column to the right of the forest plot. RRs were calculated by comparing the risk of the adverse neonatal outcome in the lockdown group with the risk in the pre-lockdown group. The number of adverse neonatal outcome events and livebirths, listed in the second to fourth columns from the left, were used to calculate the risk of adverse neonatal outcomes in each period. The black horizontal lines running through each black diamond indicate the 95% confidence intervals of the individual study RR, the exact values of the upper and lower bounds of which are defined in the brackets alongside the exact values of the RR (column right of the forest plot). The grey box surrounding each black diamond symbolizes the percentage weighting of each study in the meta-analysis, the column furthest to the right defines the exact values of the weight. The solid, vertical, black line in the centre of the plot indicates the line of no effect.

A dark blue, unfilled, diamond indicates the pooled RR and 95% confidence interval, estimated by two sided random-effects meta-analysis. Adjustments were not made for multiple comparisons. The exact values of the pooled estimate and 95% confidence intervals are defined in the corresponding row in the column to the right of the forest plot. The red, dashed, vertical line indicates the pooled RR, enabling comparison to individual studies RRs. The total number of adverse neonatal outcome events and total births per period is defined in the final row of the second to fourth columns from the left. The magnitude and significance of heterogeneity is listed in the bottom left corner for all included studies. Note: p=0.000 should be interpreted as p<0.001.

(Supplementary Figure 3. Panel A.)

(Supplementary Figure 3. Panel B.)

(Supplementary Figure 3. Panel C.)

Supplementary Figure 4. *Forest plot of risk ratios of adverse maternal outcomes over lockdown compared to pre-lockdown, outcomes include caesarean section (Panel A), OASI (Panel B), peripartum hysterectomy (Panel C), maternal readmission (Panel D), being screened positive for depression antenatally (Panel E), being screened positive for depression postpartum (Panel F) and being screened positive for anxiety antenatally (Panel G).*

Caption: Individual study risk ratios (RRs) are represented by black diamond symbols (♦), exact values are defined in the column to the right of the forest plot. RRs were calculated by comparing the risk of the adverse maternal outcome in the lockdown group with the risk in the pre-lockdown group. The number of adverse maternal outcome events and total births or women, listed in the second to fourth columns from the left, were used to calculate the risk of adverse maternal outcomes in each period. The black horizontal lines running through each black diamond indicate the 95% confidence intervals of the individual study RR, the exact values of the upper and lower bounds of which are defined in the brackets alongside the exact values of the RR (column right of the forest plot), arrows at the end of these lines indicate that the upper or lower limits of the confidence interval are higher or lower than the values defined on the x-axis. The grey box surrounding each black diamond symbolizes the percentage weighting of each study in the meta-analysis, the column furthest to the right defines the exact values of the weight. The solid, vertical, black line in the centre of the plot indicates the line of no effect. A dark blue, unfilled, diamond indicates the pooled RR and 95% confidence interval, estimated by two sided random-effects meta-analysis. Adjustments were not made for multiple comparisons. The exact values of the pooled estimate and 95% confidence intervals are defined in the corresponding row in the column to the right of the forest plot. The red, dashed, vertical line indicates the pooled RR, enabling comparison to individual studies RRs. The total number of adverse maternal outcome events and total births or women per period is defined in the final row of the second to fourth columns from the left. The magnitude and significance of heterogeneity is listed in the bottom left corner for all included studies. Note: p<0.000 should be interpreted as p<0.001.

(Supplementary Figure 4. Panel A.)

(Supplementary Figure 4. Panel B.)

(Supplementary Figure 4. Panel C.)

(Supplementary Figure 4. Panel D.)

(Supplementary Figure 4. Panel E.)

(Supplementary Figure 4. Panel F.)

(Supplementary Figure 4. Panel G.)

Supplementary Figure 5. *Forest plot of risk ratios of preterm birth (PTB) over lockdown compared to pre-lockdown, stratified by deprivation level (only area deprivation level composite indices included).*

Caption: Individual study risk ratios (RRs) are represented by black diamond symbols (♦) and grey boxes, exact values are defined in the column to the right of the forest plot. RRs were calculated by comparing the risk of PTB in the lockdown group with the risk of PTB in the pre-lockdown group. The number of PTBs and livebirths, listed in the second to fourth columns from the left, were used to calculate the risk of PTB in each period. The black horizontal lines running through each black diamond indicate the 95% confidence intervals of the individual study RR, the exact values of the upper and lower bounds of which are defined in the brackets alongside the exact values of the RR (column right of the forest plot. The grey box surrounding each black diamond symbolizes the percentage weighting of each study in the meta-analysis, the column furthest to the right defines the exact values of the weight. The solid, vertical, black line in the centre of the plot indicates the line of no effect. A dark blue, unfilled, diamond indicates the pooled RR and 95% confidence interval of all included studies and the pooled RRs of subgroups, estimated by two sided random-effects meta-analysis. Adjustments were not made for multiple comparisons. The exact values of the pooled estimate and 95% confidence intervals are defined in the corresponding row in the column to the right of the forest plot. The red, dashed, vertical line indicates the pooled RR, enabling comparison to individual studies RRs. The total number of PTBs and livebirths per period and the total in each subgroup is defined in the final row of the second to fourth columns from the left, and where "total" is defined for each subgroup. The magnitude and significance of heterogeneity is listed in the bottom left corner for all included studies and below each subgroup.

Supplementary Figure 6.

Title: Funnel plot of individual studies included in meta-analyses of more than 10 studies. ABPOs with more than 10 studies included in meta-analysis are as follows: PTB (Panel A), stillbirth (Panel B), LBW (Panel C), neonatal admissions (Panel D), caesarean section (Panel E), and positive screening for postpartum depression (Panel F).

Caption: Each included study is represented by a dark blue dot and plotted according to their estimated risk ratio (transformed to logarithms to present as a +/- change relative to 0) (x-axis), and the individual studies' precision (based on the logarithm of the standard error) (y axis). The solid black line indicates the pooled estimate obtained by two-sided random effects meta-analysis, and the dotted lines indicate the 95% confidence interval. The triangular area outlined by the dashed black lines indicates where studies should lie if bias is not present or minimal. A one-sided Egger's test is used to assess if there is significant asymmetry in the plot, if so, publication bias is present in the meta-analysis. The egger's test p value for the meta-analyses of each outcome is defined in brackets in each panel's label.

(Supplementary Figure 6. Panel A. Label: Funnel plot of studies reporting PTB [Egger's test p-value=0.233])

(Supplementary Figure 6. Panel B. Label: Funnel plot of studies reporting stillbirth [Egger's test p-value=0.128])

(Supplementary Figure 6. Panel C. Label: Funnel plot of studies reporting LBW [Egger's test p-value=0.262])

(Supplementary Figure 6. Panel D. Label: Funnel plot of studies reporting neonatal admissions [Egger's test p-value=0.788])

(Supplementary Figure 6. Panel E. Label: Funnel plot of studies reporting caesarean section [Egger's test p-value=0.363])

(Supplementary Figure 6. Panel F. Label: Funnel plot of studies reporting positive screening for postpartum depression [Egger's test p-value=0.849])

Supplementary Figure 7.

Title: Forest plot of risk ratios of preterm birth over second lockdowns (Panel A) and post-lockdown (Panel B) compared to pre-lockdown.

Caption:

Individual study risk ratios (RRs) are represented by black diamond symbols (♦), exact values are defined in the column to the right of the forest plot. RRs were calculated by comparing the risk of PTB in the lockdown group with the risk of PTB in the pre-lockdown group. The number of PTBs and livebirths, listed in the second to fourth columns from the left, were used to calculate the risk of PTB in each period. The black horizontal lines running through each black diamond indicate the 95% confidence intervals of the individual study RR, the exact values of the upper and lower bounds of which are defined in the brackets alongside the exact values of the RR (column right of the forest plot), arrows at the end of these lines indicate that the upper or lower limits of the confidence interval are higher or lower than the values defined on the x-axis. The grey box surrounding each black diamond symbolizes the percentage weighting of each study in the meta-analysis, the column furthest to the right defines the exact values of the weight. The solid, vertical, black line in the centre of the plot indicates the line of no effect. A dark blue, unfilled, diamond indicates the pooled RR and 95% confidence interval of all included studies and the pooled RRs of subgroups, estimated by two sided random-effects meta-analysis. Adjustments were not made for multiple comparisons. The exact values of the pooled estimate and 95% confidence intervals are defined in the corresponding row in the column to the right of the forest plot. The red, dashed, vertical line indicates the pooled RR, enabling comparison to individual studies RRs. The total number of PTBs and livebirths per period and the total in each subgroup is defined in the final row of the second to fourth columns from the left, and where "total" is defined for each subgroup. The magnitude and significance of heterogeneity is listed in the bottom left corner for all included studies and below each subgroup. Note: p<0.000 should be interpreted as p<0.001.

(Supplementary Figure 7: Panel A.)

(Supplementary Figure 7: Panel B.)

Supplementary Figure 8.

Title: Forest plot of risk ratios of spontaneous PTB (Panel A) and iatrogenic PTB over lockdown compared to pre-lockdown.

Caption:

Individual study risk ratios (RRs) are represented by black diamond symbols (♦), exact values are defined in the column to the right of the forest plot. RRs were calculated by comparing the risk of spontaneous or iatrogenic PTB in the lockdown group with the risk in the pre-lockdown group. The number of spontaneous or iatrogenic PTBs and livebirths, listed in the second to fourth columns from the left, were

used to calculate the risk of spontaneous or iatrogenic PTB in each period. The black horizontal lines running through each black diamond indicate the 95% confidence intervals of the individual study RR, the exact values of the upper and lower bounds of which are defined in the brackets alongside the exact values of the RR (column right of the forest plot), arrows at the end of these lines indicate that the upper or lower limits of the confidence interval are higher or lower than the values defined on the x-axis. The grey box surrounding each black diamond symbolizes the percentage weighting of each study in the meta-analysis, the column furthest to the right defines the exact values of the weight. The solid, vertical, black line in the centre of the plot indicates the line of no effect. A dark blue, unfilled, diamond indicates the pooled RR and 95% confidence interval of all included studies, estimated by two sided random-effects meta-analysis. Adjustments were not made for multiple comparisons. The exact values of the pooled estimate and 95% confidence intervals are defined in the corresponding row in the column to the right of the forest plot. The red, dashed, vertical line indicates the pooled RR, enabling comparison to individual studies RRs. The total number of PTBs and livebirths per period is defined in the final row of the second to fourth columns from the left. The magnitude and significance of heterogeneity is listed in the bottom left corner for all included studies and below each subgroup. Note: $p<0.000$ should be interpreted as $p<0.001$.

(Supplementary Figure 8. Panel A.)

(Supplementary Figure 8. Panel B.)

Supplementary Figure 9.

Title: Forest plot of risk ratios of extreme PTB (Panel A), very PTB (Panel B), and moderate to late PTB (Panel C) over lockdown compared to pre-lockdown.

Caption:

Individual study risk ratios (RRs) are represented by black diamond symbols (♦), exact values are defined in the column to the right of the forest plot. RRs were calculated by comparing the risk of extreme, very or moderate to late PTB in the lockdown group with the risk in the pre-lockdown group. The number of extreme, very or moderate to late PTBs and livebirths, listed in the second to fourth columns from the left, were used to calculate the risk of extreme, very or moderate to late PTB in each period. The black horizontal lines running through each black diamond indicate the 95% confidence intervals of the individual study RR, the exact values of the upper and lower bounds of which are defined in the brackets alongside the exact values of the RR (column right of the forest plot), arrows at the end of these lines indicate that the upper or lower limits of the confidence interval are higher or lower than the values defined on the x-axis. The grey box surrounding each black diamond symbolizes the percentage weighting of each study in the meta-analysis, the column furthest to the right defines the exact values of the weight. The solid, vertical, black line in the centre of the plot indicates the line of no effect. A dark blue, unfilled, diamond indicates the pooled RR and 95% confidence interval of all included studies, estimated by two sided random-effects meta-analysis. Adjustments were not made for multiple comparisons. The exact values of the pooled estimate and 95% confidence intervals are defined in the

corresponding row in the column to the right of the forest plot. The red, dashed, vertical line indicates the pooled RR, enabling comparison to individual studies RRs. The total number of PTBs and livebirths per period is defined in the final row of the second to fourth columns from the left. The magnitude and significance of heterogeneity is listed in the bottom left corner for all included studies and below each subgroup. Note: p<0.000 should be interpreted as p<0.001.

(Supplementary Figure 9. Panel A.)

(Supplementary Figure 9. Panel B.)

(Supplementary Figure 9. Panel C.)

Supplementary Figure 10.

Title: Forest plot of risk ratios of adverse neonatal outcomes over lockdown compared to pre-lockdown, outcomes include SGA (Panel A), VLBW (Panel B), ELBW (Panel C), and LBW by regional sub-group (Panel D).

Caption: Individual study risk ratios (RRs) are represented by black diamond symbols (♦), exact values are defined in the column to the right of the forest plot. RRs were calculated by comparing the risk of the adverse neonatal outcome in the lockdown group with the risk in the pre-lockdown group. The number of adverse neonatal outcome events and livebirths, listed in the second to fourth columns from the left, were used to calculate the risk of adverse neonatal outcomes in each period. The black horizontal lines running through each black diamond indicate the 95% confidence intervals of the individual study RR, the exact values of the upper and lower bounds of which are defined in the brackets alongside the exact values of the RR (column right of the forest plot). The grey box surrounding each black diamond symbolizes the percentage weighting of each study in the meta-analysis, the column furthest to the right defines the exact values of the weight. The solid, vertical, black line in the centre of the plot indicates the line of no effect. A dark blue, unfilled, diamond indicates the pooled RR and 95% confidence interval, and the pooled RRs of subgroups, estimated by two sided random-effects meta-analysis. Adjustments were not made for multiple comparisons. The exact values of the pooled estimate and 95% confidence intervals are defined in the corresponding row in the column to the right of the forest plot. The red, dashed, vertical line indicates the pooled RR, enabling comparison to individual studies RRs. The total number of adverse neonatal outcome events and total births per period and subgroup is defined in the final row of the second to fourth columns from the left. The magnitude and significance of heterogeneity is listed in the bottom left corner for all included studies. Note: p=0.000 should be interpreted as p<0.001.

(Supplementary Figure 10. Panel A.)

(Supplementary Figure 10. Panel B.)

(Supplementary Figure 10. Panel C.)

(Supplementary Figure 10. Panel D.)

Supplementary Figure 11.

Title: Forest plot of risk ratios of neonatal admissions over lockdown compared to pre-lockdown, with studies stratified by regional sub-group.

Caption: Individual study risk ratios (RRs) are represented by black diamond symbols (♦), exact values are defined in the column to the right of the forest plot. RRs were calculated by comparing the risk of the neonatal admissions in the lockdown group with the risk in the pre-lockdown group. The number of neonatal admissions and livebirths, listed in the second to fourth columns from the left, were used to calculate the risk of neonatal admission in each period and subgroup. The black horizontal lines running through each black diamond indicate the 95% confidence intervals of the individual study RR, the exact values of the upper and lower bounds of which are defined in the brackets alongside the exact values of the RR (column right of the forest plot). The grey box surrounding each black diamond symbolizes the percentage weighting of each study in the meta-analysis, the column furthest to the right defines the exact values of the weight. The solid, vertical, black line in the centre of the plot indicates the line of no effect. A dark blue, unfilled, diamond indicates the pooled RR and 95% confidence interval, and the pooled RRs of subgroups, estimated by two sided random-effects meta-analysis. Adjustments were not made for multiple comparisons. The exact values of the pooled estimate and 95% confidence intervals are defined in the corresponding row in the column to the right of the forest plot. The red, dashed, vertical line indicates the pooled RR, enabling comparison to individual studies RRs. The total number of neonatal admissions and total births per period and subgroup is defined in the final row of the second to fourth columns from the left. The magnitude and significance of heterogeneity is listed in the bottom left corner for all included studies. Note: p=0.000 should be interpreted as p<0.001.

Supplementary Figure 12.

Title: Forest plot of risk ratios of caesarean section subclassifications over lockdown compared to pre-lockdown, outcomes include planned caesarean section (Panel A), emergency caesarean section (Panel B), and overall caesarean section with studies stratified by regional subgroup (Panel C).

Caption: Individual study risk ratios (RRs) are represented by black diamond symbols (♦), exact values are defined in the column to the right of the forest plot. RRs were calculated by comparing the risk of caesarean section outcomes in the lockdown group with the risk in the pre-lockdown group. The number of caesarean section outcome events and total women, listed in the second to fourth columns from the left, were used to calculate the risk of caesarean section outcomes in each period. The black horizontal lines running through each black diamond indicate the 95% confidence intervals of the individual study RR, the exact values of the upper and lower bounds of which are defined in the brackets alongside the exact values of the RR (column right of the forest plot), arrows at the end of these lines indicate that the upper

or lower limits of the confidence interval are higher or lower than the values defined on the x-axis. The grey box surrounding each black diamond symbolizes the percentage weighting of each study in the meta-analysis, the column furthest to the right defines the exact values of the weight. The solid, vertical, black line in the centre of the plot indicates the line of no effect. A dark blue, unfilled, diamond indicates the pooled RR and 95% confidence interval, and the pooled RRs of subgroups, estimated by two sided random-effects meta-analysis. Adjustments were not made for multiple comparisons. The exact values of the pooled estimate and 95% confidence intervals, and subgroups, are defined in the corresponding row in the column to the right of the forest plot. The red, dashed, vertical line indicates the pooled RR, enabling comparison to individual studies RRs. The total number of caesarean section outcomes and total women per period and subgroup is defined in the final row of the second to fourth columns from the left. The magnitude and significance of heterogeneity is listed in the bottom left corner for all included studies. Note: p<0.000 should be interpreted as p<0.001.

(Supplementary Figure 12. Panel A.)

(Supplementary Figure 12. Panel B.)

(Supplementary Figure 12. Panel C.)

Supplementary Figure 13.

Title: Forest plot of risk ratios of maternal readmissions over lockdown compared to pre-lockdowns, with studies stratified by regional subgroups.

Caption: Individual study risk ratios (RRs) are represented by black diamond symbols (♦), exact values are defined in the column to the right of the forest plot. RRs were calculated by comparing the risk of maternal readmission in the lockdown group with the risk in the pre-lockdown group. The number of maternal readmissions and total women, listed in the second to fourth columns from the left, were used to calculate the risk of maternal readmission in each period. The black horizontal lines running through each black diamond indicate the 95% confidence intervals of the individual study RR, the exact values of the upper and lower bounds of which are defined in the brackets alongside the exact values of the RR (column right of the forest plot), arrows at the end of these lines indicate that the upper or lower limits of the confidence interval are higher or lower than the values defined on the x-axis. The grey box surrounding each black diamond symbolizes the percentage weighting of each study in the meta-analysis, the column furthest to the right defines the exact values of the weight. The solid, vertical, black line in the centre of the plot indicates the line of no effect. A dark blue, unfilled, diamond indicates the pooled RR and 95% confidence interval, and the pooled RRs of subgroups, estimated by two sided random-effects meta-analysis. Adjustments were not made for multiple comparisons. The exact values of the pooled estimate and 95% confidence intervals, and subgroups, are defined in the corresponding row in the column to the right of the forest plot. The red, dashed, vertical line indicates the pooled RR, enabling comparison to individual studies RRs. The total number of maternal readmissions and total women per period and subgroup is defined in the final row of the second to fourth columns from the left. The

magnitude and significance of heterogeneity is listed in the bottom left corner for all included studies. Note: p<0.000 should be interpreted as p<0.001.

Supplementary Figure 14.

Title: Forest plot of risk ratios of adverse maternal outcomes over lockdown compared to pre-lockdowns, with studies stratified by regional subgroup. Outcomes include being screened positive for depression antenatally (Panel A), and being screened positive for anxiety antenatally (Panel B).

Caption: Individual study risk ratios (RRs) are represented by black diamond symbols (♦), exact values are defined in the column to the right of the forest plot. RRs were calculated by comparing the risk of adverse maternal outcomes in the lockdown group with the risk in the pre-lockdown group. The number of adverse maternal outcomes and total pregnant women, listed in the second to fourth columns from the left, were used to calculate the risk of adverse maternal outcomes in each period. The black horizontal lines running through each black diamond indicate the 95% confidence intervals of the individual study RR, the exact values of the upper and lower bounds of which are defined in the brackets alongside the exact values of the RR (column right of the forest plot), arrows at the end of these lines indicate that the upper or lower limits of the confidence interval are higher or lower than the values defined on the x-axis. The grey box surrounding each black diamond symbolizes the percentage weighting of each study in the meta-analysis, the column furthest to the right defines the exact values of the weight. The solid, vertical, black line in the centre of the plot indicates the line of no effect. A dark blue, unfilled, diamond indicates the pooled RR and 95% confidence interval, and the pooled RRs of subgroups, estimated by two sided random-effects meta-analysis. Adjustments were not made for multiple comparisons. The exact values of the pooled estimate and 95% confidence intervals, and subgroups, are defined in the corresponding row in the column to the right of the forest plot. The red, dashed, vertical line indicates the pooled RR, enabling comparison to individual studies RRs. The total number of adverse maternal outcome events and total pregnant women per period and subgroup is defined in the final row of the second to fourth columns from the left. The magnitude and significance of heterogeneity is listed in the bottom left corner for all included studies. Note: p<0.000 should be interpreted as p<0.001.

(Supplementary Figure 14. Panel A.)

(Supplementary Figure 14. Panel B.)

Supplementary Figure 15.

Title: Funnel plots of individual studies included in meta-analyses of more than 10 studies. ABPOs with more than 10 studies included in meta-analysis are as follows: spontaneous PTB (Panel A), iatrogenic PTB (Panel B), extreme PTB (Panel C), very PTB (Panel D), moderate to late PTB (Panel E), VLBW (Panel F), planned caesarean section (Panel G) and emergency caesarean section (Panel H).

Caption: Each included study is represented by a dark blue dot and plotted according to their estimated risk ratio (transformed to logarithms to present as a +/- change relative to 0) (x-axis), and the individual studies' precision (based on the logarithm of the standard error) (y axis). The solid black line indicates the pooled estimate obtained by two-sided random effects meta-analysis, and the dotted lines indicate the 95% confidence interval. The triangular area outlined by the dashed black lines indicates where studies should lie if bias is not present or minimal. A one-sided Egger's test is used to assess if there is significant asymmetry in the plot, if so, publication bias is present in the meta-analysis. The egger's test p value for the meta-analyses of each outcome is defined in the label on the bottom left of each figure.

(Supplementary Figure 15. Panel A)

(Supplementary Figure 15. Panel B)

(Supplementary Figure 15. Panel C)

(Supplementary Figure 15. Panel D)

(Supplementary Figure 15. Panel E)

(Supplementary Figure 15. Panel F)

(Supplementary Figure 15. Panel G)

(Supplementary Figure 15. Panel H)

Supplementary Figure 1: Panel A

First Author Last Name & Year	Lockdown Events	Lockdown Livebirths	Pre−Lockdown Events	Pre−Lockdown Livebirths	Risk Ratio (95% CI)	% Weight
Arnaez 2021	93	1499	4333	65201	0.94 (0.77, 1.14)	1.73
Athar 2022	247	4336	166	3154	1.08 (0.89, 1.31)	1.83
Balsa 2022	2480	25181	4457	45720	1.01 (0.96, 1.06)	5.18
Been 2020	1273	26621	26618	505202	0.91 (0.86, 0.96)	4.96
Einarsdottir 2021	43	1057	221	3954	0.74 (0.54, 1.02)	0.82
Fresson 2022	4799	96076	167234	3083456	0.92 (0.90, 0.95)	5.60
Gemmill 2021	152005	1516206	1978639	19906408	1.01 (1.00, 1.01)	5.85
Gurol−Urganci 2022	7403	124907	29983	492076	0.97 (0.95, 1.00)	5.65
Hiiragi 2021	30	279	31	339	1.16 (0.72, 1.87)	0.40
Hui 2022	1406	24732	3029	49892	0.94 (0.88, 1.00)	4.78
Huseynova 2021	266	1763	1054	5463	0.81 (0.72, 0.92)	3.03
Hwang 2022	13995	255024	168361	3481423	1.13 (1.11, 1.15)	5.76
Jasper 2022	900	10701	1971	22034	0.94 (0.88, 1.02)	4.36
Lau 2023	30706	592392	103429	1808414	0.91 (0.90, 0.92)	5.81
Leibovitch 2021	1169	25639	3886	77903	0.92 (0.86, 0.98)	4.71
Liu 2021	9140	135777	47226	714407	1.02 (1.00, 1.04)	5.70
Mak 2023	231	3723	313	5064	1.00 (0.85, 1.18)	2.22
Maki 2023	55	1648	232	5753	0.83 (0.62, 1.11)	0.97
Mcdonnell 2020	325	4397	801	9373	0.87 (0.77, 0.99)	3.04
Mikus 2021	308	2714	334	3268	1.10 (0.95, 1.27)	2.55
Molholm Hansen 2022	3505	60143	14850	243592	0.96 (0.92, 0.99)	5.44
Molina−Merino 2021	665	9478	4255	51484	0.86 (0.79, 0.93)	4.26
Muin 2021	716	12473	3985	66176	0.96 (0.88, 1.03)	4.31
Oakley 2022	1850	31839	11647	202678	1.01 (0.96, 1.06)	5.16
Okawa 2022	5	187	11	185	0.46 (0.16, 1.31)	0.09
Rusconi 2023	25550	362129	85947	1117172	0.92 (0.91, 0.94)	5.80
Overall, DL (I^2 = 96.1%, p = 0.000)	259165	3330921	2663013	31969791	0.96 (0.93, 0.99)	100.00

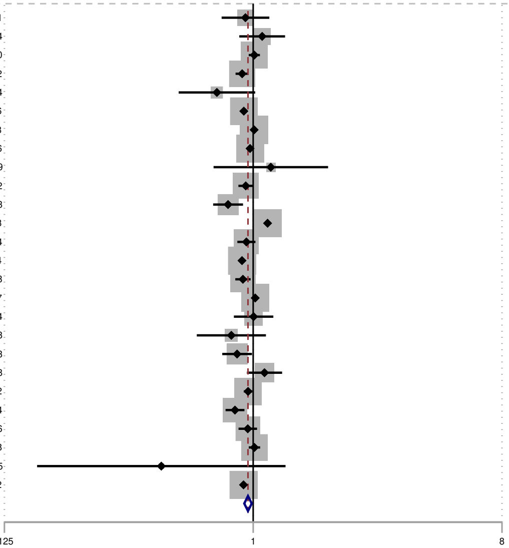

Supplementary Figure 1: Panel B

Subgroup and First Author Last Name & Year	Lockdown Events	Lockdown Livebirths	Pre−Lockdown Events	Pre−Lockdown Livebirths	Risk Ratio (95% CI)	% Weight
North America						
Gemmill 2021	152005	1516206	1978639	19906408	1.01 (1.00, 1.01)	5.85
Liu 2021	9140	135777	47226	714407	1.02 (1.00, 1.04)	5.70
Subgroup, DL (I^2 = 0.0%, p = 0.417)					1.01 (1.00, 1.01)	11.55
North America Total:	161145	1651983	2025865	20620815		
Europe						
Arnaez 2021	93	1499	4333	65201	0.94 (0.77, 1.14)	1.73
Been 2020	1273	26621	26618	505202	0.91 (0.86, 0.96)	4.96
Einarsdottir 2021	43	1057	221	3954	0.74 (0.54, 1.02)	0.82
Fresson 2022	4799	96076	167234	3083456	0.92 (0.90, 0.95)	5.60
Gurol−Urganci 2022	7403	124907	29983	492076	0.97 (0.95, 1.00)	5.65
Lau 2023	30706	592392	103429	1808414	0.91 (0.90, 0.92)	5.81
Mcdonnell 2020	325	4397	801	9373	0.87 (0.77, 0.99)	3.04
Mikus 2021	308	2714	334	3268	1.10 (0.95, 1.27)	2.55
Molholm Hansen 2022	3505	60143	14850	243592	0.96 (0.92, 0.99)	5.44
Molina−Merino 2021	665	9478	4255	51484	0.86 (0.79, 0.93)	4.26
Muin 2021	716	12473	3985	66176	0.96 (0.88, 1.03)	4.31
Oakley 2022	1850	31839	11647	202678	1.01 (0.96, 1.06)	5.16
Rusconi 2023	25550	362129	85947	1117172	0.92 (0.91, 0.94)	5.80
Subgroup, DL (I^2 = 77.0%, p = 0.000)					0.94 (0.92, 0.96)	55.13
Europe Total:	77236	1325725	453637	7652046		
Middle East						
Athar 2022	247	4336	166	3154	1.08 (0.89, 1.31)	1.83
Huseynova 2021	266	1763	1054	5463	0.81 (0.72, 0.92)	3.03
Leibovitch 2021	1169	25639	3886	77903	0.92 (0.86, 0.98)	4.71
Subgroup, DL (I^2 = 68.5%, p = 0.042)					0.91 (0.81, 1.03)	9.56
Middle East Total:	1682	31738	5106	86520		
Asia						
Hiiragi 2021	30	279	31	339	1.16 (0.72, 1.87)	0.40
Hwang 2022	13995	255024	168361	3481423	1.13 (1.11, 1.15)	5.76
Mak 2023	231	3723	313	5064	1.00 (0.85, 1.18)	2.22
Maki 2023	55	1648	232	5753	0.83 (0.62, 1.11)	0.97
Okawa 2022	5	187	11	185	0.46 (0.16, 1.31)	0.09
Subgroup, DL (I^2 = 55.0%, p = 0.064)					1.02 (0.89, 1.18)	9.45
Asia Total:	14316	260861	168948	3492764		
Australia						
Hui 2022	1406	24732	3029	49892	0.94 (0.88, 1.00)	4.78
Jasper 2022	900	10701	1971	22034	0.94 (0.88, 1.02)	4.36
Subgroup, DL (I^2 = 0.0%, p = 0.914)					0.94 (0.90, 0.99)	9.14
Australia Total:	2309	35433	5000	71926		
South America						
Balsa 2022	2480	25181	4457	45720	1.01 (0.96, 1.06)	5.18
Subgroup, DL (I^2 = 0.0%, p = .)					1.01 (0.96, 1.06)	5.18
Heterogeneity between groups: p = 0.000						
Overall, DL (I^2 = 96.1%, p = 0.000)					0.96 (0.93, 0.99)	100.00

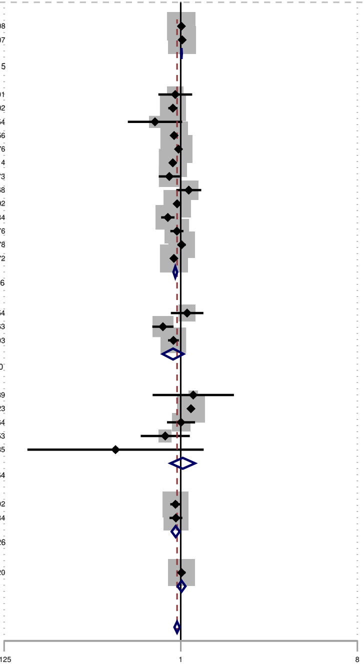

Supplementary Figure 1: Panel C

Ethnicity Group and First Author Last Name & Year	Lockdown Events	Lockdown Livebirths	Pre–Lockdown Events	Pre–Lockdown Livebirths	Risk Ratio (95% CI)	% Weight
White						
Ayyash 2022	4974	57836	5888	65420	0.96 (0.92, 0.99)	5.68
Cate 2022	141	1212	801	6123	0.90 (0.76, 1.07)	2.25
Handley 2022	76	1258	180	2526	0.86 (0.66, 1.11)	1.21
Harvey 2021	438	4881	2876	27742	0.88 (0.80, 0.97)	3.94
Hwang 2022	2172	26329	12324	143708	0.96 (0.92, 1.01)	5.49
Ibrahim 2025	17718	330952	42624	700429	0.89 (0.87, 0.90)	6.02
Janevic 2021	140	1994	156	2188	0.99 (0.79, 1.23)	1.56
Lemon 2021	378	3845	1323	12247	0.92 (0.82, 1.02)	3.58
Main 2021	2324	38284	9839	163547	1.01 (0.97, 1.05)	5.49
Subgroup, DL (I^2 = 82.7%, p < 0.000)					0.94 (0.89, 0.98)	35.23
White Total:	28361	466591	76011	1123930		
Black						
Ayyash 2022	2441	16160	2771	16997	0.94 (0.89, 0.98)	5.31
Cate 2022	175	799	808	4932	1.28 (1.10, 1.48)	2.64
Handley 2022	175	1481	383	3140	0.97 (0.82, 1.15)	2.26
Harvey 2021	184	1364	1256	8147	0.89 (0.77, 1.03)	2.71
Hwang 2022	290	2301	1253	11111	1.10 (0.98, 1.25)	3.27
Ibrahim 2025	1440	22679	3366	46473	0.88 (0.83, 0.94)	5.04
Janevic 2021	65	491	65	451	0.93 (0.67, 1.28)	0.85
Lemon 2021	130	1015	487	3641	0.96 (0.80, 1.15)	2.05
Main 2021	801	7404	3189	31195	1.05 (0.98, 1.13)	4.62
Subgroup, DL (I^2 = 76.9%, p < 0.000)					0.99 (0.92, 1.07)	28.75
Black Total:	5701	53694	13578	126087		
Other						
Cate 2022	28	217	176	1220	0.91 (0.62, 1.32)	0.65
Handely 2022	64	693	128	1457	1.05 (0.79, 1.40)	1.03
Harvey 2021	21	164	100	822	1.05 (0.67, 1.63)	0.48
Hwang 2022	376	3762	2000	19980	1.00 (0.90, 1.11)	3.69
Ibrahim 2025	667	12968	1637	28048	0.89 (0.81, 0.97)	4.19
Subgroup, DL (I^2 = 0.0%, p = 0.441)					0.94 (0.88, 1.00)	10.04
Other Total:	1156	17804	4041	51527		
Hispanic						
Cate 2022	46	400	264	2119	0.93 (0.69, 1.25)	0.98
Harvey 2021	99	952	367	3893	1.09 (0.88, 1.35)	1.66
Hwang 2022	1224	13374	6327	67837	0.98 (0.93, 1.04)	5.08
Janevic 2021	67	674	74	688	0.93 (0.68, 1.28)	0.88
Main 2021	4783	61238	21426	270293	0.99 (0.96, 1.02)	5.81
Subgroup, DL (I^2 = 0.0%, p = 0.878)					0.99 (0.96, 1.01)	14.41
Hispanic Total:	6219	76638	28458	344830		
Asian						
Cate 2022	14	151	80	856	0.99 (0.58, 1.71)	0.33
Harvey 2021	20	192	94	973	1.07 (0.68, 1.70)	0.45
Ibrahim 2025	3631	59627	8310	113593	0.84 (0.81, 0.87)	5.64
Main 2021	1378	19505	6261	92742	1.04 (0.99, 1.10)	5.15
Subgroup, DL (I^2 = 92.3%, p < 0.000)					0.96 (0.80, 1.15)	11.56
Asian Total:	5043	79475	14745	208164		
Heterogeneity between groups: p = 0.268						
Overall, DL (I^2 = 80.5%, p < 0.000)					0.96 (0.93, 0.99)	100.00
Total:	45234	676398	132792	1803011		

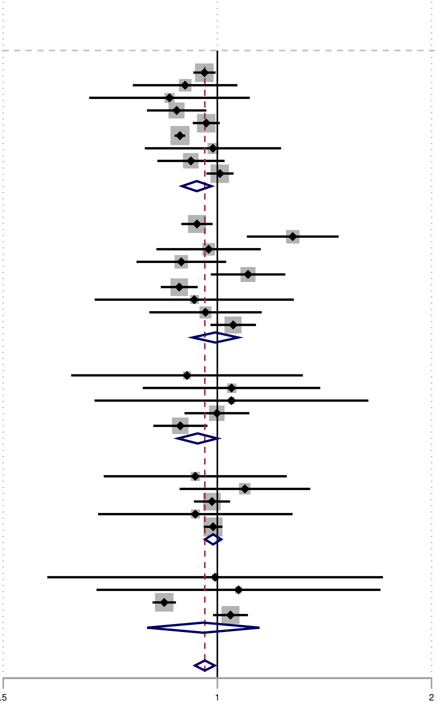

Supplementary Figure 1: Panel D

Deprivation level and First Author Last Name & Year	Lockdown Events	Lockdown Livebirths	Pre–Lockdown Events	Pre–Lockdown Livebirths	Risk Ratio (95% CI)	% Weight
**Low Deprivation**						
Aboulatta 2022	641	8492	2211	28481	0.97 (0.90, 1.06)	5.39
Been 2020	222	5379	4897	101520	0.86 (0.75, 0.98)	2.95
Fischer 2022	486	6318	483	6334	1.01 (0.89, 1.14)	3.34
Gurol–Urganci 2022	11508	220677	13381	250027	0.98 (0.95, 1.00)	11.62
Harvey 2021	448	4778	2607	24643	0.90 (0.81, 0.99)	4.64
Lemon 2021	170	2163	565	7034	0.98 (0.83, 1.16)	2.05
Shah 2021	3172	45322	65818	860236	0.92 (0.89, 0.95)	10.52
Subgroup, DL (I^2 = 52.2%, p = 0.051)					0.95 (0.91, 0.98)	40.50
Low Deprivation Total:	16647	293129	89962	1278275		
**Medium Deprivation**						
Been 2020	800	16299	16629	308177	0.91 (0.85, 0.98)	6.69
Harvey 2021	269	2275	1385	11337	0.97 (0.86, 1.10)	3.25
Lemon 2021	140	1385	532	4442	0.86 (0.72, 1.02)	1.82
Shah 2021	2139	26921	37734	475350	1.00 (0.96, 1.04)	9.64
Subgroup, DL (I^2 = 56.0%, p = 0.078)					0.95 (0.89, 1.02)	21.40
Medium Deprivation Total:	3348	46880	56280	799306		
**High Deprivation**						
Aboulatta 2022	806	8295	2707	26758	0.96 (0.89, 1.04)	6.17
Been 2020	251	4943	5092	95505	0.95 (0.84, 1.08)	3.25
Fischer 2022	188	1804	221	1720	0.83 (0.69, 1.00)	1.70
Gurol–Urganci 2022	13986	206262	16393	238330	0.99 (0.97, 1.01)	11.86
Harvey 2021	108	1054	694	5510	0.83 (0.69, 1.01)	1.57
Lemon 2021	187	1409	715	5130	0.96 (0.82, 1.11)	2.37
Shah 2021	4648	54259	80898	981126	1.04 (1.01, 1.07)	11.20
Subgroup, DL (I^2 = 63.9%, p = 0.011)					0.98 (0.94, 1.02)	38.10
High Deprivation Total:	20174	278026	106720	1354079		
Heterogeneity between groups: p = 0.503						
Overall, DL (I^2 = 65.0%, p < 0.000)					0.96 (0.94, 0.99)	100.00
Total:	40169	618035	252962	3431660		

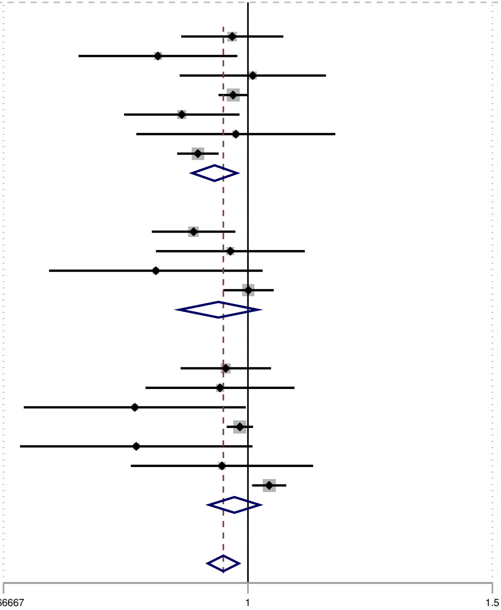

NOTE: Weights and between–subgroup heterogeneity test are from random–effects model

Supplementary Figure 1: Panel E

First Author Last Name & Year of Publication (Population)	Births	RR (95%CI)	Weight %
Been 2020 (Netherlands)	796531	0.86 [0.75, 0.99]	1.74
Calvert 2023 (Australia, New South Wales)	518281	0.97 [0.88, 1.07]	3.28
Calvert 2023 (Australia, Queensland)	58204	0.87 [0.67, 1.13]	0.52
Calvert 2023 (Belgium)	665086	0.96 [0.87, 1.06]	3.22
Calvert 2023 (Canada)	1.6e+06	0.95 [0.89, 1.01]	6.44
Calvert 2023 (Chile)	1.2e+06	0.97 [0.91, 1.04]	5.90
Calvert 2023 (Denmark, Central Region)	66481	1.15 [0.88, 1.50]	0.52
Calvert 2023 (Finland)	278376	1.07 [0.91, 1.26]	1.33
Calvert 2023 (Hong Kong)	199134	1.07 [0.90, 1.28]	1.14
Calvert 2023 (Hungary)	501860	0.98 [0.87, 1.10]	2.45
Calvert 2023 (Iceland)	23463	0.72 [0.41, 1.27]	0.12
Calvert 2023 (Norway)	316067	0.85 [0.74, 0.98]	1.70
Calvert 2023 (Poland, Single Facility 1)	8287	0.83 [0.32, 2.13]	0.04
Calvert 2023 (Poland, Single Facility 2)	42243	0.87 [0.66, 1.15]	0.47
Calvert 2023 (Scotland)	288118	1.05 [0.93, 1.18]	2.34
Calvert 2023 (Switzerland)	486357	1.04 [0.93, 1.16]	2.66
Calvert 2023 (USA)	2.1e+07	0.97 [0.95, 1.00]	14.56
Calvert 2023 (Uruguay)	232514	1.03 [0.88, 1.20]	1.45
Calvert 2023 (Wales)	176964	0.96 [0.84, 1.10]	1.76
Fresson 2022 (France)	3.2e+06	0.94 [0.90, 0.98]	10.06
Hwang 2022 (South Korea)	3.7e+06	0.99 [0.99, 1.00]	19.26
Maeda 2020 (Japan)	28474	0.85 [0.74, 0.98]	1.74
Morgan 2022 (England, Yorkshire & Humber)	1161	0.73 [0.56, 0.95]	0.55
Rusconi 2023 (Italy)	1.5e+06	0.96 [0.93, 1.00]	11.32
Stansfield 2022 (Australia, Melbourne)	35660	0.80 [0.05, 11.80]	0.01
Stumpfe 2022 (Germany, Bavaria)	1.3e+06	0.99 [0.92, 1.06]	5.42
Overall, DL (I^2 = 38.3%, p = 0.001)	38216801	0.97 [0.95, 0.99]	

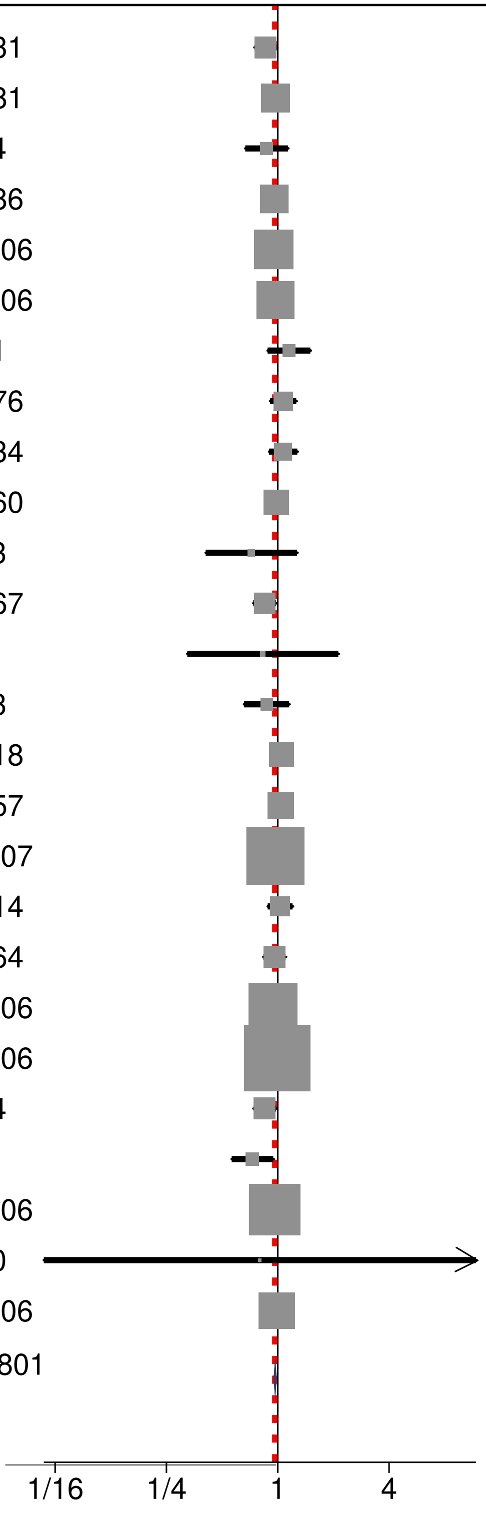

Supplementary Figure 2: Panel A

First Author Last Name & Year	Lockdown Events	Lockdown Total Births	Pre-Lockdown Events	Pre-Lockdown Total Births	Risk Ratio (95% CI)	% Weight
Arnaez 2021	5	1504	295	65496	0.74 (0.31, 1.79)	0.21
Balsa 2022	126	25307	230	45950	0.99 (0.80, 1.24)	2.83
Chen 2022	154	77193	176	88240	1.00 (0.81, 1.24)	2.84
Dagelic 2023	28	4153	21	4293	1.38 (0.78, 2.42)	0.50
Einarsdottir 2021	3	1060	8	3962	1.40 (0.37, 5.27)	0.09
Fischer 2022	17	8298	11	8246	1.53 (0.72, 3.27)	0.28
Fresson 2022	803	96880	24737	3108192	1.04 (0.97, 1.12)	10.28
Gallo 2022	6	1237	41	8796	1.04 (0.44, 2.45)	0.22
Gurol−Urganci 2022	481	126093	1818	496293	1.04 (0.94, 1.15)	7.76
Hui 2022	85	24817	125	50017	1.37 (1.04, 1.80)	1.89
Jones 2021	104	29031	562	130326	0.83 (0.67, 1.02)	3.00
Kim 2021	1	246	32	2765	0.35 (0.05, 2.58)	0.04
Lau 2023	2336	594728	6704	1815118	1.06 (1.01, 1.11)	12.36
Liu 2021	668	136445	3498	717905	1.00 (0.93, 1.09)	9.17
Mak 2023	15	3738	12	5076	1.69 (0.79, 3.62)	0.28
Meyer 2021	22	2594	290	28686	0.84 (0.55, 1.29)	0.83
Mikus 2021	18	2732	9	3277	2.39 (1.08, 5.31)	0.25
Molholm Hansen 2022	130	60323	507	244363	1.04 (0.86, 1.26)	3.41
Mor 2021	6	1556	5	4564	3.51 (1.07, 11.49)	0.12
Muin 2021	44	12517	158	66334	1.47 (1.06, 2.06)	1.34
Rusconi 2023	960	363089	3004	1120176	0.99 (0.92, 1.06)	10.04
Shukla 2023	628	74076	2125	236481	0.94 (0.86, 1.03)	8.67
Simoene 2022	3790	614093	3957	663620	1.03 (0.99, 1.08)	12.59
Speyer 2022	9	7342	10	8323	1.02 (0.41, 2.51)	0.20
Sun 2021	1422	152903	1532	172095	1.04 (0.97, 1.12)	10.11
Tate 2022	50	2590	26	2921	2.15 (1.34, 3.44)	0.70
Overall, DL (I^2 = 43.7%, p = 0.010)	11911	2424824	49893	9101854	1.04 (1.00, 1.08)	100.00

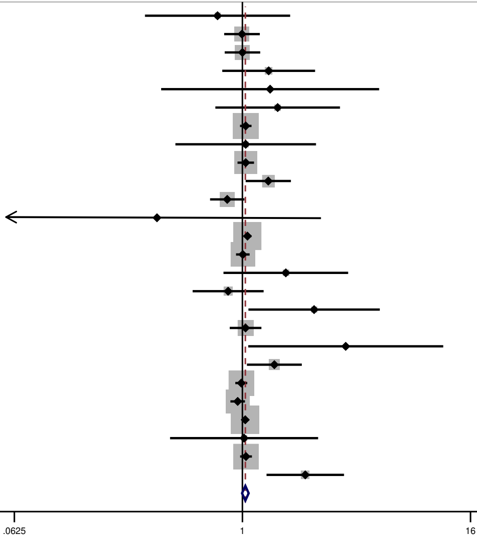

Supplementary Figure 2: Panel B

First Author Last Name & Year of Publication (Population)	Births	RR (95%CI)	Weight %
Balsa 2022 (Uruguay)	71257	1.00 [1.00, 1.00]	72.06
Calvert 2023 (Australia, New South Wales)	518281	0.82 [0.52, 1.29]	0.70
Calvert 2023 (Australia, Queensland)	58204	1.30 [0.57, 2.96]	0.21
Calvert 2023 (Belgium)	665086	1.03 [0.70, 1.51]	0.97
Calvert 2023 (Canada)	1.6e+06	0.94 [0.77, 1.15]	3.25
Calvert 2023 (Finland)	278376	1.80 [0.91, 3.57]	0.30
Calvert 2023 (Hong Kong)	199134	1.46 [0.74, 2.87]	0.31
Calvert 2023 (Hungary)	501860	0.92 [0.64, 1.33]	1.03
Calvert 2023 (Iceland)	23463	2.53 [0.91, 7.02]	0.14
Calvert 2023 (Norway)	316067	0.78 [0.41, 1.49]	0.34
Calvert 2023 (Poland, Single Facility 1)	8287	2.83 [0.54, 14.78]	0.05
Calvert 2023 (Poland, Single Facility 2)	42243	0.62 [0.23, 1.68]	0.14
Calvert 2023 (Scotland)	288118	1.09 [0.51, 2.33]	0.25
Calvert 2023 (Switzerland)	486357	0.96 [0.63, 1.46]	0.80
Calvert 2023 (Wales)	176964	0.91 [0.55, 1.51]	0.56
Fresson 2022 (France)	3.2e+06	1.05 [0.96, 1.15]	14.10
Morgan 2022 (England, Yorkshire & Humber)	1161	1.21 [0.98, 1.49]	3.11
Stumpfe 2022 (Germany, Bavaria)	161538	1.20 [0.90, 1.60]	1.69
Overall, DL (I = 4.92, p = 0.413)	8611979	1.02 [0.98, 1.06]	100

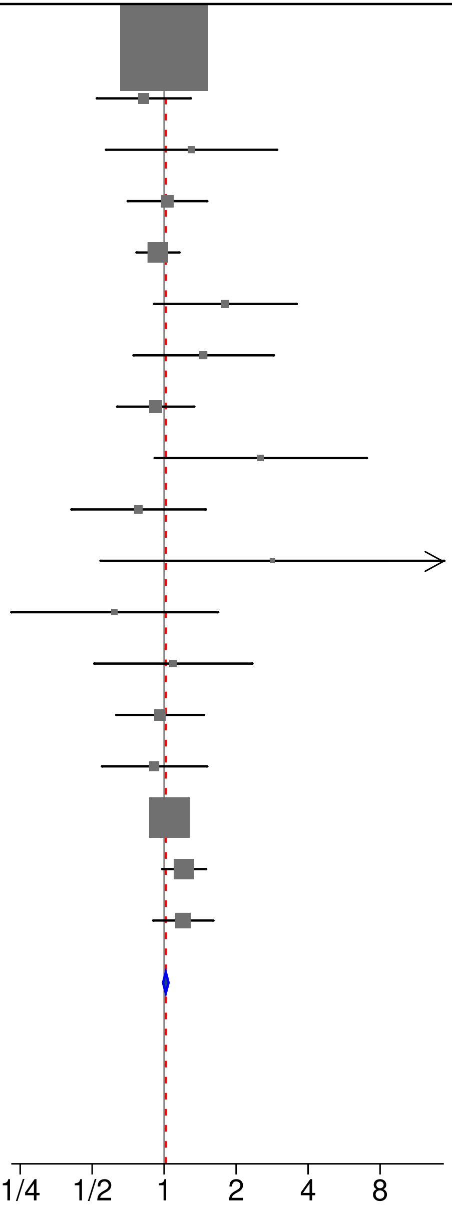

Supplementary Figure 3: Panel A

First Author Last Name & Year	Lockdown Events	Lockdown Livebirths	Pre−Lockdown Events	Pre−Lockdown Livebirths	Risk Ratio (95% CI)	% Weight
Alshaikh 2021	39	4334	348	24018	0.62 (0.45, 0.87)	1.52
Amadori 2021	16	256	19	293	0.97 (0.51, 1.84)	0.43
Arnaez 2021	17	1499	653	65201	1.13 (0.70, 1.83)	0.76
Balsa 2022	1645	25181	3109	45720	0.96 (0.91, 1.02)	12.41
Benyamini Raischer 2023	113	2442	136	2323	0.80 (0.63, 1.02)	2.58
Esposito 2023	824	13435	1017	14902	0.90 (0.83, 0.99)	9.46
Garabedian 2021	464	3809	460	4071	1.07 (0.95, 1.21)	6.95
Gemmill 2021	124574	1516206	1636908	1.99e+07	1.00 (0.99, 1.00)	16.03
Hwang 2022	10242	255024	129807	3481423	1.07 (1.05, 1.10)	15.54
Jones 2021	1875	28927	8218	129764	1.02 (0.97, 1.07)	13.30
Kirchengast 2021	26	669	1539	29084	0.74 (0.51, 1.09)	1.17
Llorca 2021	34	620	83	969	0.66 (0.45, 0.97)	1.13
Mak 2023	324	3723	375	5064	1.16 (1.01, 1.34)	5.73
Maki 2023	107	1650	454	5762	0.83 (0.68, 1.02)	3.44
Okawa 2022	18	187	18	185	0.99 (0.53, 1.85)	0.45
Philip 2020	3	1381	49	5663	0.25 (0.08, 0.81)	0.13
Rolnik 2021	281	3187	328	3229	0.88 (0.75, 1.02)	5.28
Stumpfe 2022	110	16015	1190	145018	0.84 (0.69, 1.02)	3.70
Overall, DL (I^2 = 81.9%, p = 0.000)	140712	1878545	1784711	23862689	0.97 (0.93, 1.01)	100.00

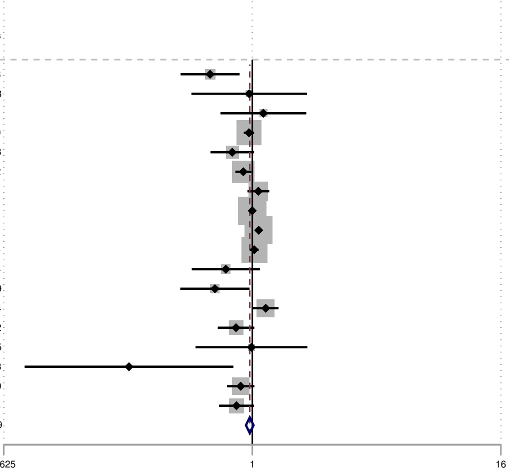

Supplementary Figure 3: Panel B

First Author Last Name & Year	Lockdown Events	Lockdown Livebirths	Pre–Lockdown Events	Pre–Lockdown Livebirths	Risk Ratio (95% CI)	% Weight
Ameh 2021	2	1461	1	1553	2.12 (0.19, 23.40)	0.19
Dagelic 2023	19	4153	21	4293	0.94 (0.50, 1.74)	2.92
Jones 2021	15	28927	78	129764	0.86 (0.50, 1.50)	3.67
Meyer 2021	3	2572	23	28396	1.44 (0.43, 4.79)	0.77
Quibel 2022	11	3885	15	4147	0.78 (0.36, 1.70)	1.86
Rao 2022	2	1058	7	1384	0.37 (0.08, 1.80)	0.45
Rolnik 2021	11	3187	9	3229	1.24 (0.51, 2.98)	1.45
Shukla 2023	257	73448	1031	234356	0.80 (0.69, 0.91)	60.23
Simpson 2021	113	67400	704	346834	0.83 (0.68, 1.01)	28.45
Overall, DL (I^2 = 0.0%, p = 0.887)	433	186091	1889	753956	0.82 (0.74, 0.91)	100.00

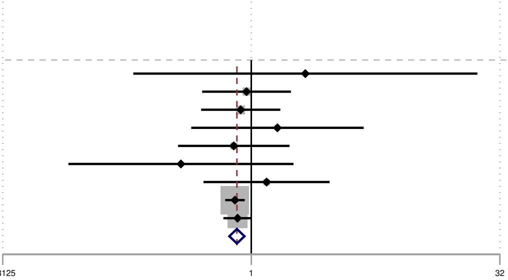

Supplementary Figure 3: Panel C

First Author Last Name & Year	Lockdown Events	Lockdown Livebirths	Pre–Lockdown Events	Pre–Lockdown Livebirths	Risk Ratio (95% CI)	% Weight
Ameh 2021	95	1461	83	1553	1.20 (0.90, 1.60)	2.01
Athar 2022	218	4336	230	3154	0.70 (0.59, 0.84)	3.70
Athiraman 2022	564	4738	1180	9538	0.97 (0.88, 1.06)	6.15
Bajaj 2022	5903	77983	19378	250896	0.98 (0.95, 1.01)	7.99
Debolt 2022	124	1078	189	1910	1.15 (0.92, 1.42)	2.99
Herzberger 2021	85	2668	82	2701	1.05 (0.78, 1.41)	1.88
Hiiragi 2021	62	279	55	339	1.30 (0.93, 1.82)	1.59
Hui 2022	551	24732	1113	49892	1.00 (0.90, 1.10)	5.94
Jeyamurugan 2023	160	360	224	427	0.89 (0.76, 1.06)	4.01
Justman 2020	49	610	59	742	1.01 (0.70, 1.45)	1.37
Khalil 2020	106	1702	103	1677	1.01 (0.78, 1.32)	2.28
Kim 2021	126	245	737	2733	1.60 (1.37, 1.87)	4.30
Maslin 2022	3815	42926	8419	91121	0.97 (0.93, 1.00)	7.83
Meyer 2021	78	2572	1048	28396	0.83 (0.66, 1.04)	2.80
Mor 2021	57	1550	212	4559	0.80 (0.60, 1.06)	2.00
Okawa 2022	10	187	13	185	0.77 (0.35, 1.72)	0.33
Quibel 2022	495	3885	478	4147	1.09 (0.97, 1.23)	5.37
Racine 2022	88	1210	57	1058	1.33 (0.96, 1.83)	1.66
Richter 2022	203	2558	1180	18849	1.25 (1.08, 1.44)	4.63
Riley 2021	2098	20704	8853	78718	0.91 (0.87, 0.95)	7.64
Roberts 2022	12495	93711	94275	702419	0.99 (0.98, 1.01)	8.14
Shukla 2023	7166	73448	20014	234356	1.13 (1.10, 1.16)	8.03
Wagner 2021	1395	33198	7271	188225	1.08 (1.03, 1.15)	7.36
Overall, DL (I^2 = 88.2%, p = 0.000)	39414	396141	168932	1677595	1.03 (0.98, 1.08)	100.00

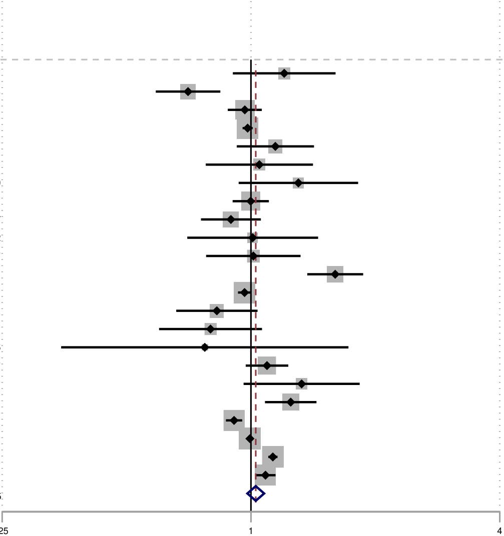

Supplementary Figure 4: Panel A

First Author Last Name & Year	Lockdown Events	Lockdown Total Births	Pre−Lockdown Events	Pre−Lockdown Total Births	Risk Ratio (95% CI)	% Weight
Balsa 2022	12020	25307	21213	45950	1.02 (1.00, 1.04)	9.72
Benyamini Raischer 2023	392	2442	400	2323	0.94 (0.83, 1.07)	1.51
Deli 2021	261	748	209	517	0.90 (0.77, 1.05)	1.10
Delius 2023	57	333	110	704	1.08 (0.80, 1.45)	0.32
Einarsdottir 2021	145	1060	643	3962	0.86 (0.73, 1.02)	0.94
Esposito 2023	3143	13476	3490	14952	1.00 (0.96, 1.04)	6.42
Fresson 2022	18963	96880	607325	3108192	1.00 (0.99, 1.01)	10.29
Gemmill 2021	482516	1516206	6344801	19906408	1.00 (1.00, 1.00)	10.93
Herzberger 2021	407	2668	435	2701	0.95 (0.84, 1.08)	1.58
Jones 2021	6224	29031	28489	130326	0.98 (0.96, 1.01)	8.90
Justman 2020	147	610	164	742	1.07 (0.88, 1.31)	0.68
Kim 2021	97	246	906	2765	1.15 (0.96, 1.37)	0.85
Llorca 2021	110	620	236	969	0.77 (0.63, 0.95)	0.64
Mak 2023	1143	3723	1305	5064	1.15 (1.07, 1.23)	3.86
Maki 2023	289	1650	1040	5762	0.97 (0.86, 1.10)	1.71
Meyer 2020	491	1666	463	1654	1.04 (0.93, 1.16)	1.92
Meyer 2021	661	2594	6990	28686	1.04 (0.97, 1.11)	3.77
Mikus 2021	881	2732	1024	3277	1.02 (0.95, 1.11)	3.31
Mor 2021	273	1556	809	4564	0.99 (0.87, 1.13)	1.56
Roberts 2022	28641	94126	200055	705767	1.06 (1.04, 1.07)	10.50
Rolnik 2021	1061	3150	989	3175	1.06 (0.98, 1.14)	3.50
Speyer 2022	2651	7342	2855	8323	1.04 (0.99, 1.09)	6.17
Suzuki 2020	40	138	43	148	1.00 (0.68, 1.46)	0.20
Wagner 2021	9002	33198	51516	188225	0.99 (0.97, 1.01)	9.55
Zanardo 2020	12	91	12	101	1.10 (0.52, 2.33)	0.05
Overall, DL (I^2 = 82.8%, p = 0.000)	569627	1841593	7275522	24175257	1.01 (1.00, 1.03)	100.00

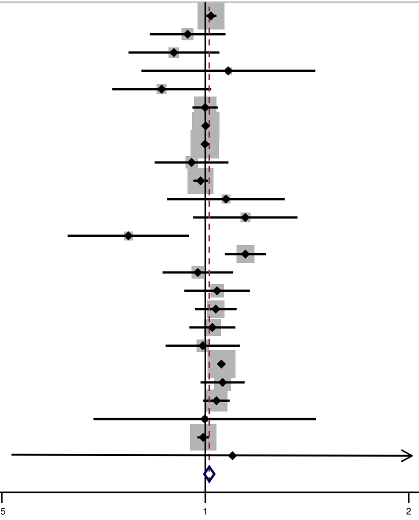

Supplementary Figure 4: Panel B

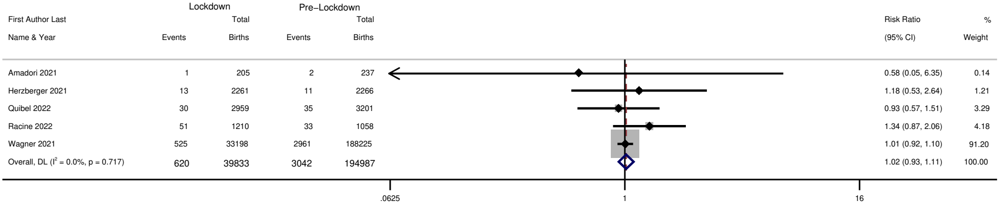

Supplementary Figure 4: Panel C

First Author Last Name & Year	Lockdown Events	Lockdown Total Births	Pre−Lockdown Events	Pre−Lockdown Total Births	Risk Ratio (95% CI)	% Weight
Debolt 2022	3	1078	2	1910	2.65 (0.44, 15.85)	4.51
Racine 2022	2	1210	3	1058	0.58 (0.10, 3.49)	4.52
Shukla 2022	54	74076	236	236481	0.73 (0.54, 0.98)	60.36
Wagner 2021	14	33198	67	188225	1.18 (0.67, 2.11)	30.61
Overall, DL (I^2 = 24.0%, p = 0.267)	73	109562	308	427674	0.89 (0.60, 1.31)	100.00

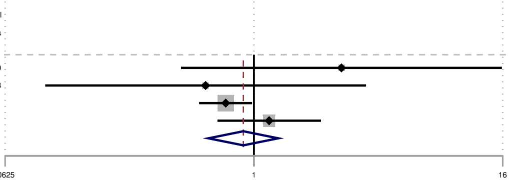

Supplementary Figure 4: Panel D

First Author Last Name & Year	Lockdown Events	Lockdown Total Births	Pre-Lockdown Events	Pre-Lockdown Total Births	Risk Ratio (95% CI)	% Weight
Debolt 2022	14	1078	19	1910	1.30 (0.66, 2.59)	6.11
Facco 2021	94	2417	88	2368	1.04 (0.79, 1.39)	13.09
Gulerson 2022	46	4038	50	4168	0.95 (0.64, 1.41)	10.68
Gurol−Urganci 2022	3161	122851	15888	418393	0.69 (0.66, 0.71)	17.10
Hirsch 2021	16	2343	13	2665	1.40 (0.67, 2.90)	5.63
Panzer 2022	22	643	25	715	0.98 (0.56, 1.72)	7.74
Racine 2022	16	1210	17	1058	0.83 (0.42, 1.63)	6.21
Riley 2021	354	20704	1178	78718	1.14 (1.01, 1.28)	16.32
Simoene 2022	7665	614093	8224	663620	1.01 (0.98, 1.04)	17.13
Overall, DL (I^2 = 97.0%, p = 0.000)	11388	769377	25502	1173615	0.98 (0.79, 1.21)	100.00

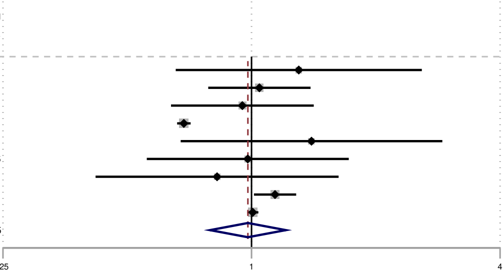

Supplementary Figure 4: Panel E

First Author Last Name & Year	Lockdown: Screened positive for depression in pregnancy	Lockdown: Pregnant Women	Pre–Lockdown: Screened positive for depression in pregnancy	Pre–Lockdown: Pregnant Women	Risk Ratio (95% CI)	% Weight
Berthelot 2020	137	1258	30	496	1.72 (1.18, 2.52)	11.64
Clifton 2022	27	156	37	243	1.12 (0.71, 1.77)	10.47
De Chiara 2022	18	72	8	68	1.90 (0.88, 4.12)	6.56
Gustafsson 2021	175	247	23	155	3.21 (2.16, 4.78)	11.40
Kuipers 2022	119	341	60	200	1.12 (0.86, 1.47)	13.29
Lantigua–Martinez 2022	2	240	12	626	0.44 (0.10, 1.95)	2.57
McKee 2023	137	785	208	1570	1.27 (1.04, 1.55)	14.20
Vacaru 2021	32	307	76	1337	1.76 (1.18, 2.61)	11.43
Zhang 2022	6	110	16	263	0.90 (0.36, 2.25)	5.36
Zilver 2021	145	1102	57	364	0.86 (0.65, 1.14)	13.07
Overall, DL (I^2 = 75.9%, p = 0.000)	798	4618	690	5692	1.37 (1.06, 1.78)	100.00

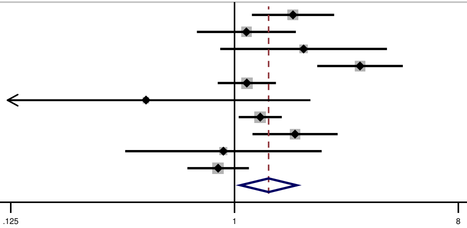

Supplementary Figure 4: Panel F

First Author Last Name & Year	Lockdown: Screened postive for depression in postpartum	Lockdown: Postpartum Women	Pre−Lockdown: Screened postive for depression in postpartum	Pre−Lockdown: Postpartum Women	Risk Ratio (95% CI)	% Weight
Bajaj 2022	8197	118622	10372	159478	1.06 (1.03, 1.09)	11.31
Boekhorst 2021	23	268	28	401	1.21 (0.71, 2.06)	3.56
Chrzan−Detkos 2021	58	78	40	61	1.08 (0.79, 1.47)	6.52
Clifton 2022	23	156	39	243	0.93 (0.57, 1.50)	4.08
Dol 2021	60	331	114	561	0.91 (0.68, 1.21)	6.94
Green 2022	73	677	55	420	0.84 (0.60, 1.17)	6.15
Gustafsson 2021	66	146	6	75	4.20 (1.90, 9.31)	1.92
Hiiragi 2021	38	279	34	339	1.32 (0.85, 2.04)	4.57
Hui 2021	23	802	69	2970	1.23 (0.77, 1.96)	4.24
Kuipers 2022	29	148	106	456	0.87 (0.60, 1.26)	5.45
Lantigua−Martinez 2022	8	310	28	938	0.87 (0.40, 1.88)	2.01
Loehr 2022	46	319	41	260	0.93 (0.62, 1.37)	5.18
Marino−Narvaez 2021	28	75	19	82	1.45 (0.86, 2.42)	3.73
Pariente 2020	38	223	39	123	0.60 (0.40, 0.90)	5.05
Sangtani 2021	25	387	49	390	0.54 (0.34, 0.86)	4.27
Silverman 2020	16	252	19	264	0.89 (0.47, 1.69)	2.70
Suzuki 2020	19	138	22	148	0.94 (0.53, 1.66)	3.19
Vitale 2021	57	156	60	110	0.76 (0.56, 1.02)	6.67
Waschmann 2021	91	504	94	557	1.06 (0.81, 1.38)	7.34
Zanardo 2020	26	91	12	101	2.09 (1.11, 3.94)	2.76
Zhang 2022	12	85	17	285	2.20 (1.09, 4.44)	2.35
Overall, DL (I^2 = 58.8%, p = 0.000)	8956	124125	11320	168638	1.01 (0.90, 1.14)	100.00

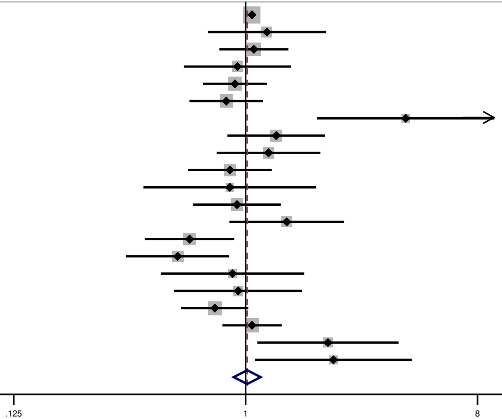

Supplementary Figure 4: Panel G

First Author Last Name & Year	Lockdown: Screened positive for anxiety in pregnancy	Lockdown: Pregnant Women	Pre−Lockdown: Screened postivie for anxiety in pregnancy	Pre−Lockdown: Pregnant Women	Risk Ratio (95% CI)	% Weight
Berthelot 2020	137	1258	30	496	1.72 (1.18, 2.52)	9.89
Clifton 2022	85	156	129	243	1.02 (0.82, 1.27)	11.17
De Chiara 2022	22	72	15	68	1.30 (0.72, 2.33)	8.02
Kuipers 2022	52	209	27	109	1.00 (0.66, 1.52)	9.58
Quistorff 2021	21	39	29	161	2.29 (1.42, 3.71)	8.98
Suzuki 2020	30	117	29	134	1.15 (0.72, 1.82)	9.18
Vacaru 2021	149	311	319	1337	1.68 (1.43, 1.98)	11.51
Vitale 2021	19	156	82	110	0.25 (0.16, 0.40)	9.22
Zhang 2022	79	110	200	263	0.97 (0.79, 1.18)	11.32
Zilver 2021	215	1102	84	364	0.87 (0.69, 1.09)	11.12
Overall, DL (I^2 = 89.4%, p < 0.000)	809	3530	944	3285	1.08 (0.81, 1.44)	100.00

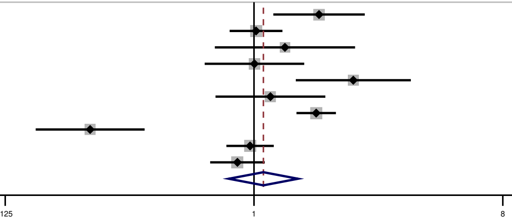

Supplementary Figure 5

Deprivation level and First Author Last Name & Year	Lockdown Events	Lockdown Livebirths	Pre−Lockdown Events	Pre−Lockdown Livebirths	Risk ratio (95% CI)	% Weight
Low Deprivation						
Been 2020	222	5379	4897	101520	0.86 (0.75, 0.98)	3.81
Fischer 2022	486	6318	483	6334	1.01 (0.89, 1.14)	4.42
Gurol−Urganci 2022	11508	220677	13381	250027	0.98 (0.95, 1.00)	32.42
Lemon 2021	170	2163	565	7034	0.98 (0.83, 1.16)	2.50
Subgroup, DL (I^2 = 18.3%, p = 0.299)					0.97 (0.93, 1.01)	43.15
Low Deprivation Total:	12386	234537	19326	364915		
Medium Deprivation						
Been 2020	800	16299	16629	308177	0.91 (0.85, 0.98)	11.24
Lemon 2021	140	1385	532	4442	0.86 (0.72, 1.02)	2.19
Subgroup, DL (I^2 = 0.0%, p = 0.518)					0.91 (0.85, 0.97)	13.43
Medium Deprivation Total:	940	17684	17161	312619		
High Deprivation						
Been 2020	251	4943	5092	95505	0.95 (0.84, 1.08)	4.28
Fischer 2022	188	1804	221	1720	0.83 (0.69, 1.00)	2.04
Gurol−Urganci 2022	13986	206262	16393	238330	0.99 (0.97, 1.01)	34.16
Lemon 2021	187	1409	715	5130	0.96 (0.82, 1.11)	2.95
Subgroup, DL (I^2 = 19.7%, p = 0.291)					0.97 (0.92, 1.02)	43.42
High Deprivation Total:	14612	214418	22421	340685		
Heterogeneity between groups: p = 0.201						
Overall, DL (I^2 = 32.5%, p = 0.148)					0.96 (0.94, 0.99)	100.00
Total:	27938	466639	58908	1018219		

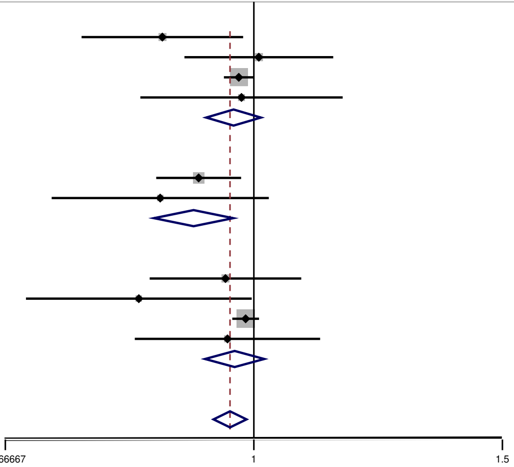

NOTE: Weights and between−subgroup heterogeneity test are from random−effects model

Supplementary Figure 6: Panel A

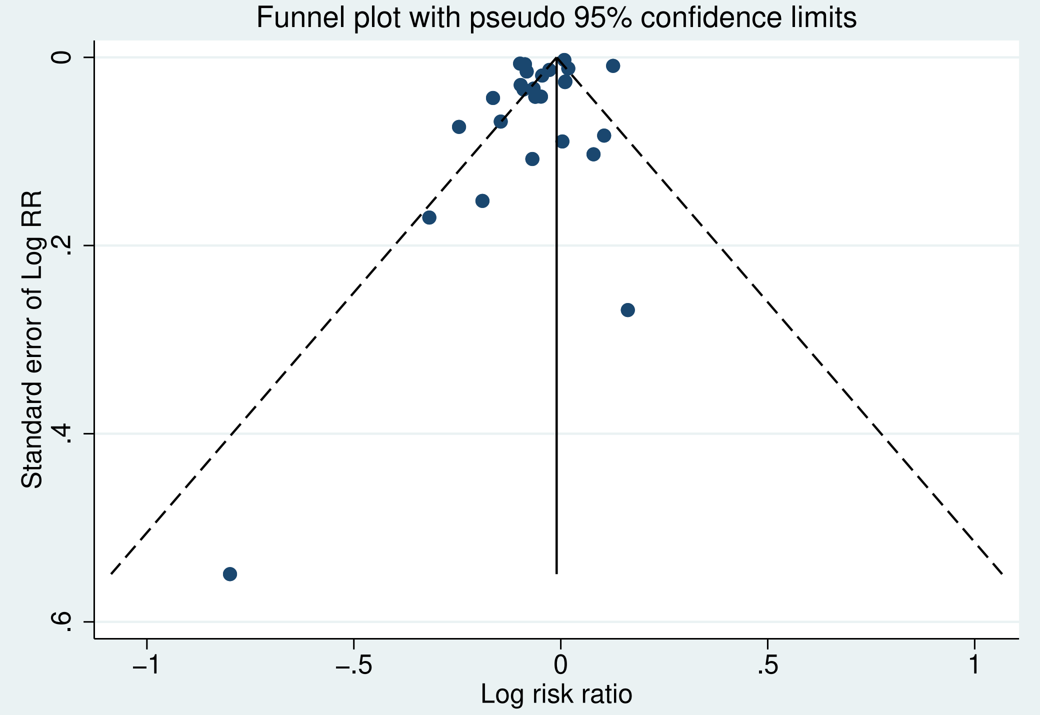

Supplementary Figure 6: Panel B

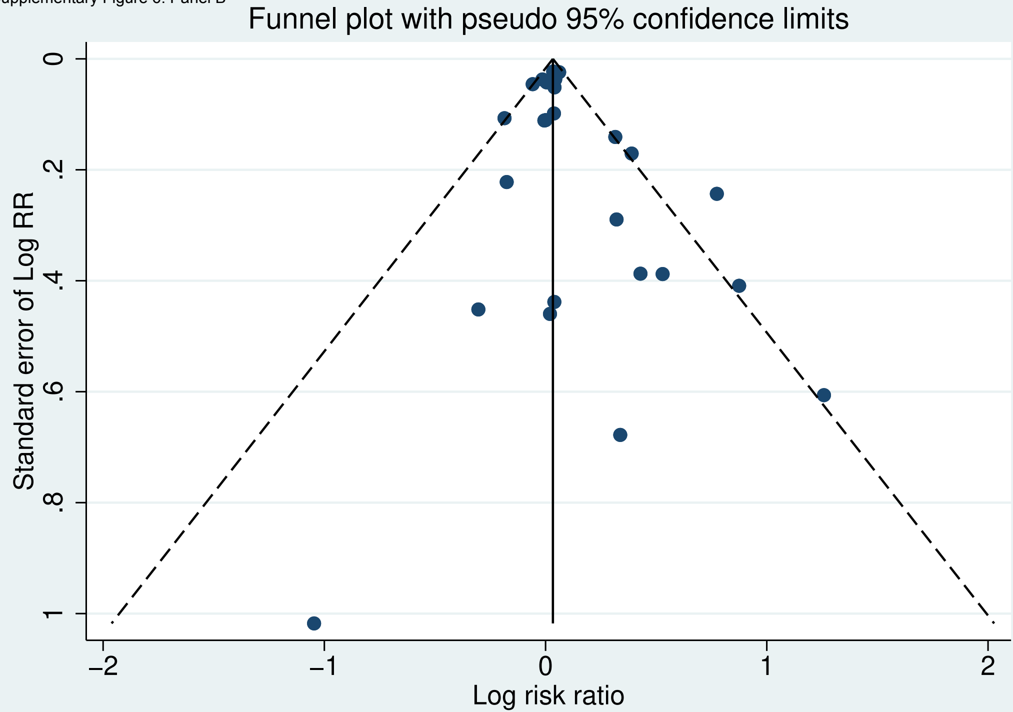

Supplementary Figure 6: Panel C

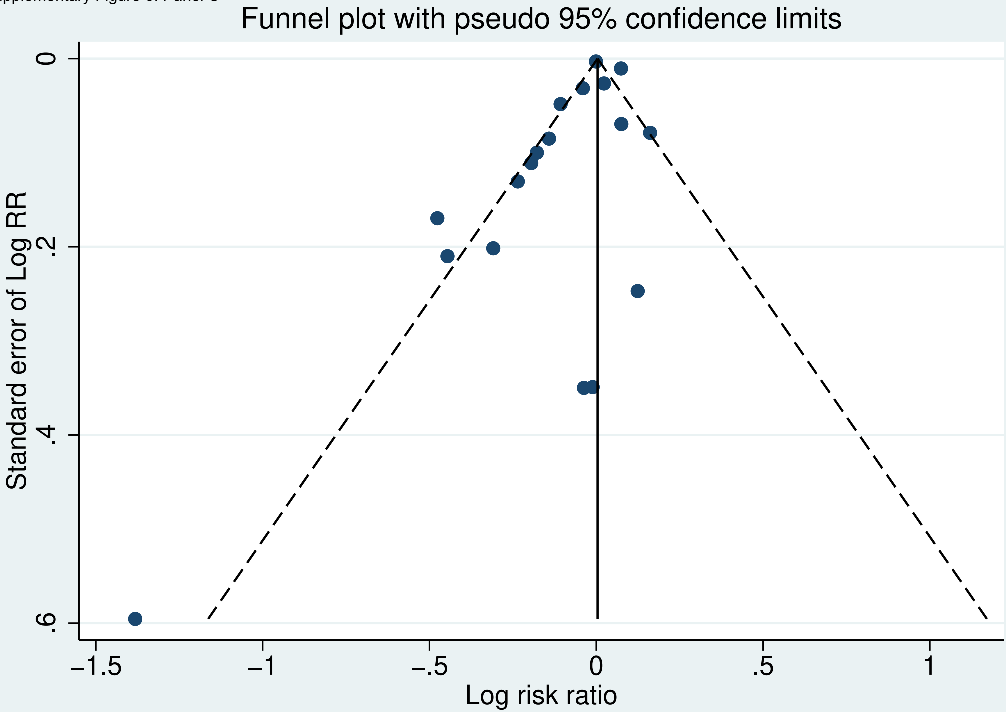

Supplementary Figure 6: Panel D

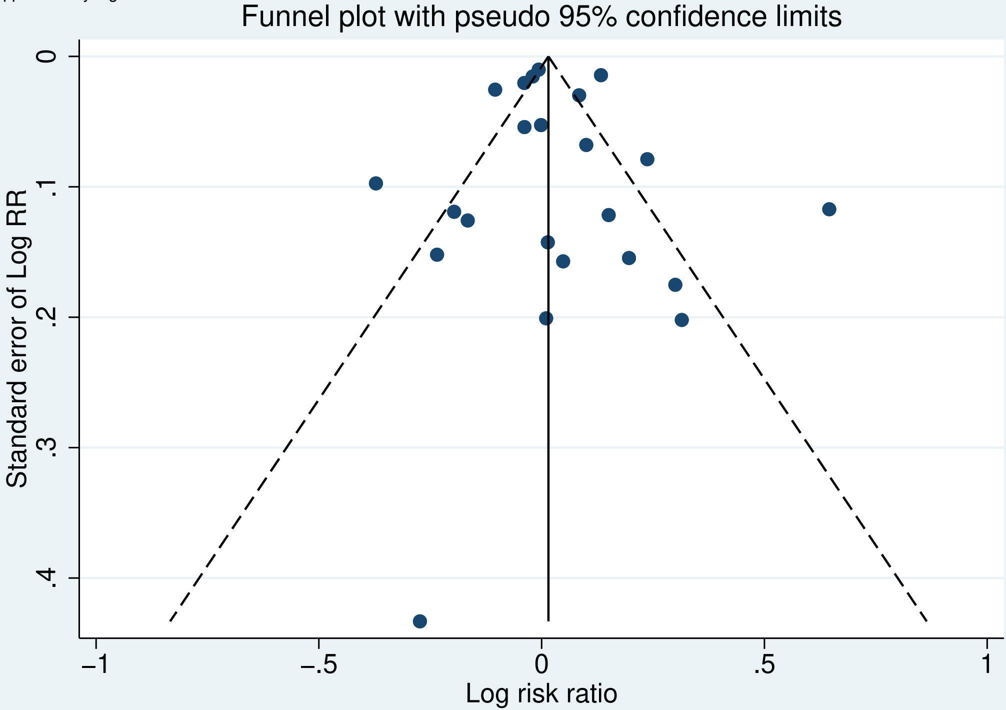

Supplementary Figure 6: Panel E

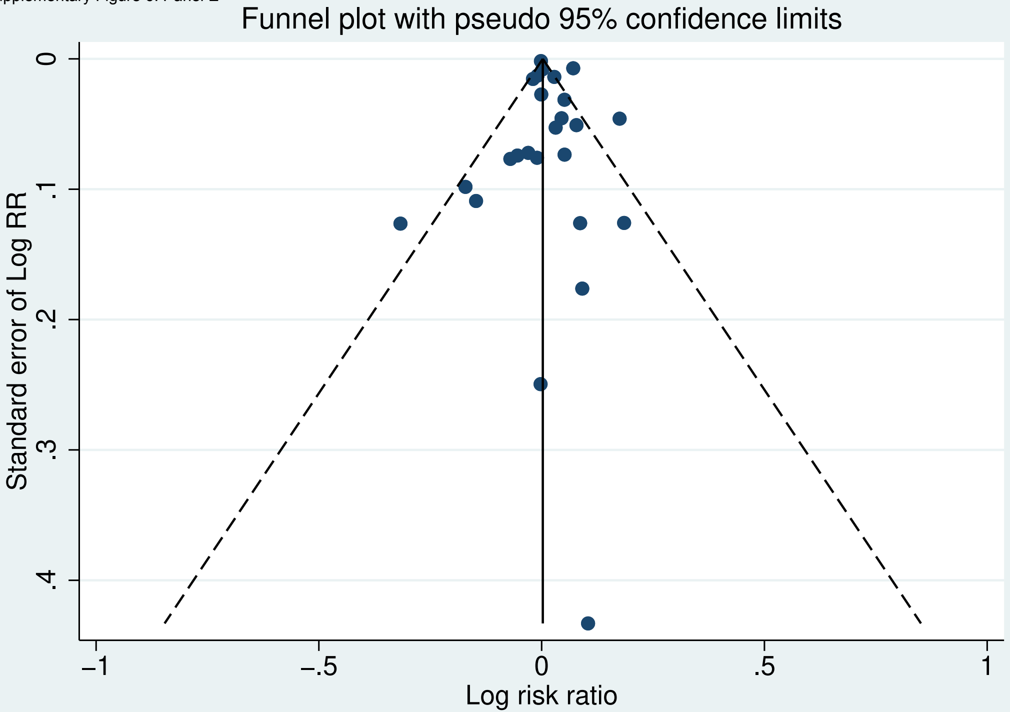

Supplementary Figure 6: Panel F

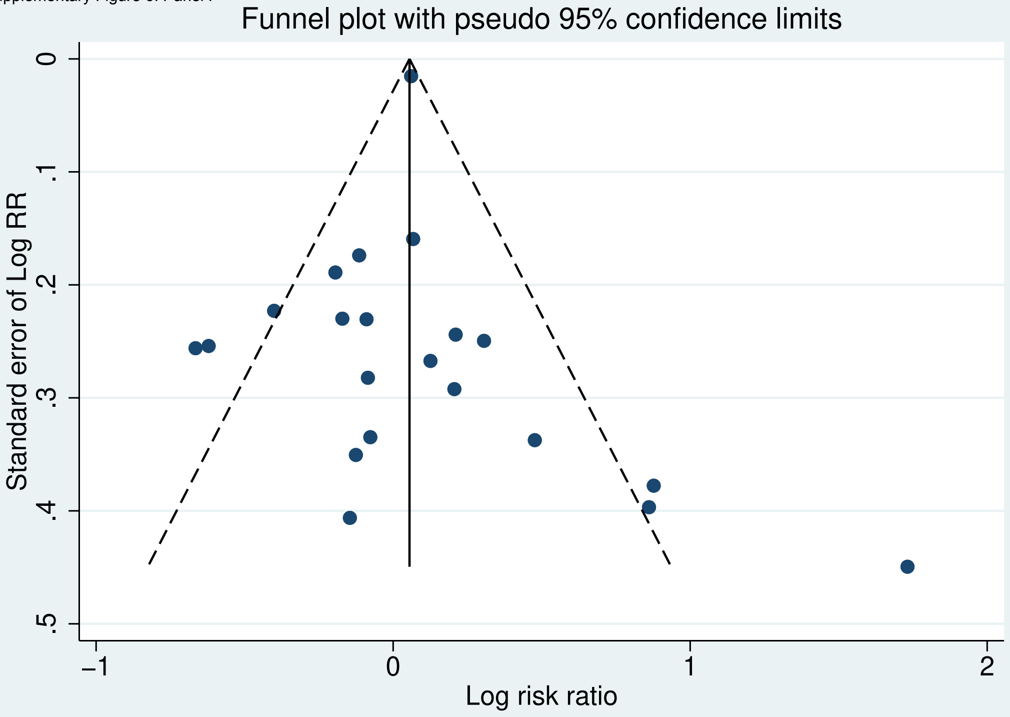

Supplementary Figure 7: Panel A

First Author Last Name & Year	Lockdown 2 Events	Lockdown 2 Livebirths	Pre-Lockdown 2 Events	Pre-Lockdown 2 Livebirths	Risk Ratio (95% CI)	% Weight
Einarsdottir 2021	58	1038	180	4009	1.23 (0.92, 1.64)	3.01
Esposito 2023	1550	25532	1858	29222	0.96 (0.90, 1.02)	18.99
Gemmill 2021	88031	871174	1978639	19906408	1.02 (1.01, 1.02)	26.59
Gurol-Urganci 2022	11082	181609	13649	222014	0.99 (0.97, 1.02)	25.27
Ohashi 2023	154	6021	456	11936	0.68 (0.57, 0.81)	6.55
Stumpfe 2022	1037	18159	11156	168989	0.87 (0.82, 0.93)	19.59
Overall, DL (I^2 = 89.8%, p = 0.000)	101912	1103533	2005938	20342578	0.95 (0.90, 1.00)	100.00

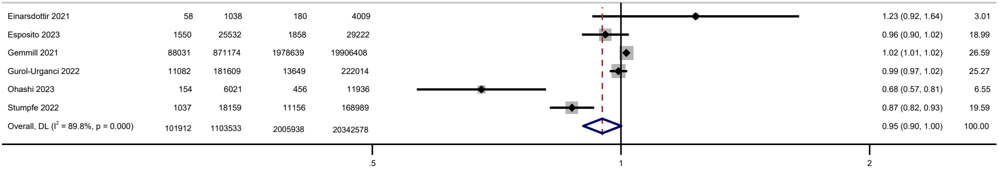

Supplementary Figure 7: Panel B

First Author Last Name & Year	Post-Lockdown Events	Post-Lockdown Livebirths	Pre-Lockdown Events	Pre-Lockdown Livebirths	Risk Ratio (95% CI)	% Weight
Arnaez 2021	102	1489	4333	65201	1.03 (0.85, 1.24)	8.51
Garabedian 2021	308	3992	286	4147	1.11 (0.95, 1.30)	12.13
Jasper 2022	947	10686	1945	21568	0.98 (0.91, 1.06)	35.87
Leibovitch 2021	1219	25875	3943	78792	0.94 (0.89, 1.01)	43.13
Okawa 2022	6	181	11	185	0.57 (0.22, 1.51)	0.36
Overall, DL (I^2 = 23.4%, p = 0.265)	2582	42223	10518	169893	0.98 (0.93, 1.04)	100.00

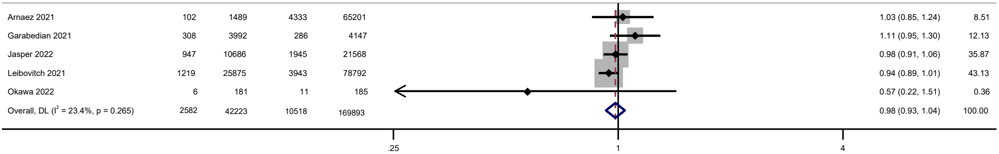

Supplementary Figure 8: Panel A

First Author Last Name & Year	Lockdown Events	Lockdown Livebirths	Pre-Lockdown Events	Pre-Lockdown Livebirths	Risk Ratio (95% CI)	% Weight
Berghella 2020	57	1197	59	911	0.75 (0.52, 1.06)	1.79
Einarsdottir 2021	24	1057	105	3954	0.86 (0.55, 1.33)	1.20
Fischer 2022	415	8281	444	8235	0.93 (0.82, 1.06)	9.70
Handley 2022	159	3432	336	7123	0.98 (0.82, 1.18)	5.74
Hui 2022	662	24732	1397	49892	0.96 (0.87, 1.05)	14.99
Jasper 2022	16	547	52	1095	0.63 (0.36, 1.09)	0.77
Lemon 2021	272	5396	1004	17687	0.89 (0.78, 1.02)	9.68
Mak 2023	95	3723	87	5064	1.47 (1.10, 1.96)	2.64
Oakley 2022	1017	31839	6813	202678	0.95 (0.89, 1.02)	20.21
Rao 2022	48	1058	58	1384	1.08 (0.74, 1.57)	1.62
Roberts 2022	2877	93711	23583	702419	0.92 (0.88, 0.95)	26.35
Wood 2021	203	4712	193	4644	1.04 (0.85, 1.26)	5.32
Overall, DL (I^2 = 36.0%, p = 0.102)	5845	179685	34131	1005086	0.95 (0.90, 0.99)	100.00

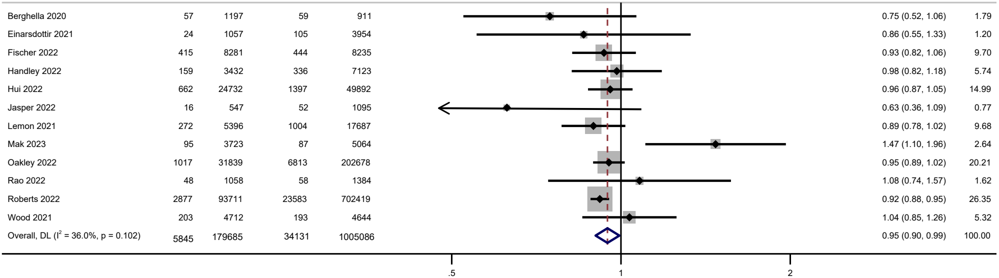

Supplementary Figure 8: Panel B

First Author Last Name & Year	Lockdown Events	Lockdown Livebirths	Pre-Lockdown Events	Pre-Lockdown Livebirths	Risk Ratio (95% CI)	% Weight
Adeluwoye 2021	19	567	23	560	0.82 (0.45, 1.49)	1.58
Berghella 2020	60	1197	56	911	0.82 (0.58, 1.17)	3.79
David 2023	137	38782	562	119586	0.75 (0.62, 0.91)	8.51
Einarsdottir 2021	19	1057	116	3954	0.62 (0.38, 1.00)	2.31
Fischer 2022	262	8281	286	8235	0.91 (0.77, 1.08)	9.50
Handley 2022	156	3432	355	7123	0.92 (0.76, 1.10)	8.62
Hui 2022	744	24732	1632	49892	0.92 (0.85, 1.00)	13.65
Jasper 2022	11	547	39	1095	0.57 (0.30, 1.11)	1.31
Lemon 2021	273	5396	959	17687	0.94 (0.82, 1.07)	11.21
Oakley 2022	833	31839	4834	202678	1.09 (1.02, 1.18)	14.29
Rao 2022	44	1058	24	1384	2.34 (1.43, 3.83)	2.22
Roberts 2022	2981	93711	22243	702419	1.00 (0.97, 1.04)	15.68
Wood 2021	169	4712	151	4644	1.10 (0.89, 1.36)	7.33
Overall, DL (I^2 = 71.1%, p = 0.000)	5708	215311	31280	1120168	0.95 (0.88, 1.03)	100.00

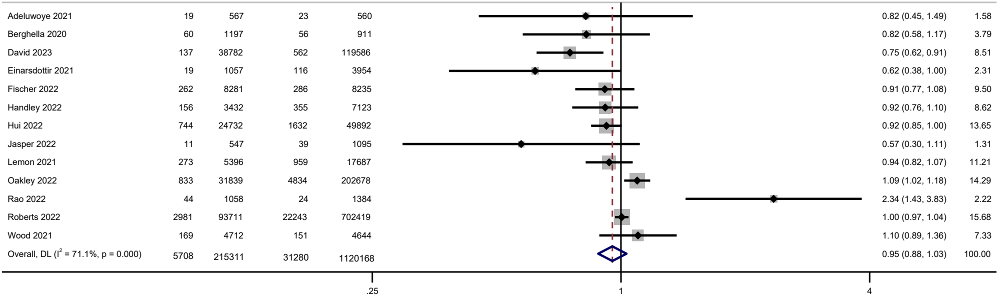

Supplementary Figure 9: Panel A.

First Author Last Name & Year	Lockdown Events	Lockdown Livebirths	Pre-Lockdown Events	Pre-Lockdown Livebirths	Risk Ratio (95% CI)	% Weight
Arnaez 2021	8	1499	181	65201	1.92 (0.95, 3.89)	0.80
Athiraman 2022	34	4738	63	9538	1.09 (0.72, 1.65)	2.03
Berghella 2020	7	1197	14	911	0.38 (0.16, 0.95)	0.51
Cate 2022	45	2646	218	15574	1.21 (0.88, 1.67)	3.05
Cheung 2021	49	5546	89	6007	0.60 (0.42, 0.85)	2.69
David 2023	245	38782	642	119586	1.18 (1.02, 1.36)	6.97
Feldman 2021	3	1135	8	1306	0.43 (0.12, 1.63)	0.24
Fresson 2022	259	96076	8494	3083456	0.98 (0.87, 1.11)	7.76
Handley 2022	23	3432	58	7123	0.82 (0.51, 1.33)	1.59
Hui 2022	51	24732	119	49892	0.86 (0.62, 1.20)	2.93
Huseynova 2021	23	1763	107	5463	0.67 (0.43, 1.05)	1.80
Jasper 2022	116	10701	205	22034	1.16 (0.93, 1.46)	4.71
Jones 2021	167	28927	753	129764	0.99 (0.84, 1.18)	6.32
Kim 2021	2	245	49	2733	0.46 (0.11, 1.88)	0.21
Klumper 2021	76	26924	285	107326	1.06 (0.83, 1.37)	4.15
Lemon 2021	45	5396	176	17687	0.84 (0.61, 1.16)	2.95
Main 2021	592	132853	2578	580714	1.00 (0.92, 1.10)	8.93
Mak 2023	13	3723	19	5064	0.93 (0.46, 1.88)	0.81
Maki 2023	4	1648	9	5753	1.55 (0.48, 5.03)	0.30
Mikus 2021	43	2714	28	3268	1.84 (1.14, 2.95)	1.64
Molholm Hansen 2022	150	60143	711	243592	0.85 (0.72, 1.02)	6.06
Molina-Merino 2021	27	9478	150	51484	0.98 (0.65, 1.47)	2.08
Muin 2021	40	12473	213	66176	1.00 (0.71, 1.40)	2.81
Rasmussen 2021	188	3378	201	3536	0.98 (0.81, 1.19)	5.55
Rusconi 2023	999	362129	3504	1117172	0.88 (0.82, 0.94)	9.52
Shah 2021	638	126740	13296	2324866	0.88 (0.81, 0.95)	9.24
Stumpfe 2022	50	16015	477	145018	0.95 (0.71, 1.27)	3.46
Wood 2021	20	4712	14	4644	1.41 (0.71, 2.78)	0.86
Overall, DL (I^2 = 51.6%, $p < 0.000$)	3917	989745	32661	8194888	0.97 (0.91, 1.03)	100.00

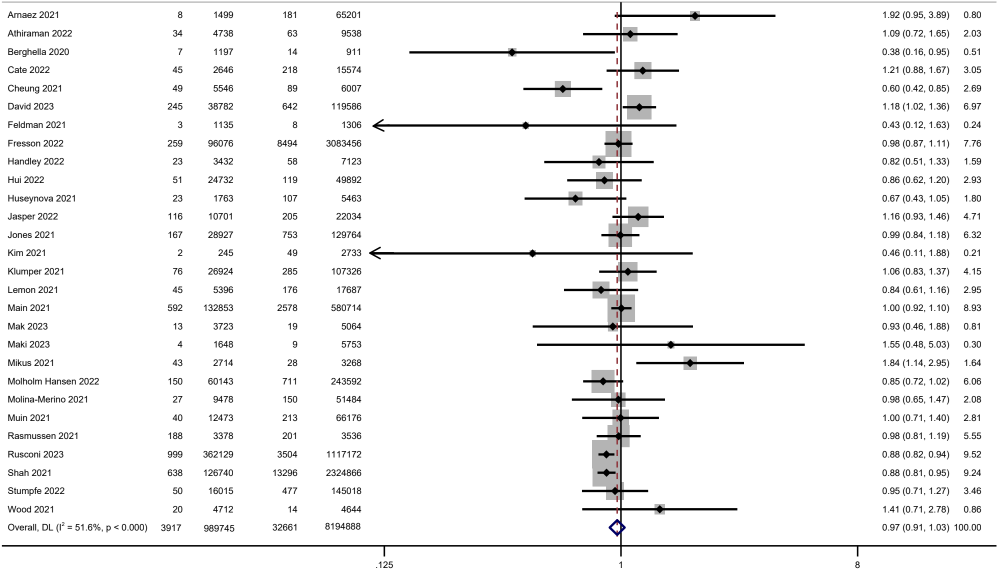

Supplementary Figure 9: Panel B

First Author Last Name & Year	Lockdown Events	Lockdown Livebirths	Pre-Lockdown Events	Pre-Lockdown Livebirths	Risk Ratio (95% CI)	% Weight
Arnaez 2021	6	1499	392	65201	0.67 (0.30, 1.49)	1.39
Athiraman 2022	79	4738	195	9538	0.82 (0.63, 1.06)	3.68
Balsa 2022	405	25181	643	45720	1.14 (1.01, 1.29)	4.33
Cheung 2021	43	5546	77	6007	0.61 (0.42, 0.88)	3.06
David 2023	777	38782	2603	119586	0.92 (0.85, 1.00)	4.46
Fresson 2022	482	96076	15405	3083456	1.00 (0.92, 1.10)	4.43
Handley 2022	28	3432	86	7123	0.68 (0.44, 1.04)	2.78
Hui 2022	194	24732	422	49892	0.93 (0.78, 1.10)	4.14
Huseynova 2021	55	1763	160	5463	1.06 (0.79, 1.44)	3.45
Janevic 2021	53	3834	62	4192	0.94 (0.65, 1.35)	3.10
Jasper 2022	81	10701	169	22034	0.99 (0.76, 1.29)	3.66
Jones 2021	262	28927	1207	129764	0.97 (0.85, 1.11)	4.29
Kim 2021	3	245	108	2733	0.32 (0.10, 0.99)	0.81
Klumper 2021	200	26924	942	107326	0.85 (0.73, 0.99)	4.22
Leibovitch 2021	120	25639	481	77903	0.76 (0.62, 0.93)	4.00
Main 2021	1491	132853	3531	580714	1.84 (1.73, 1.95)	4.50
Mak 2023	43	3723	62	5064	0.94 (0.64, 1.39)	2.98
Maki 2023	3	1648	24	5753	0.44 (0.13, 1.45)	0.75
Maslin 2022	404	42926	927	91121	0.93 (0.82, 1.04)	4.35
Mikus 2021	99	2714	99	3268	1.20 (0.91, 1.57)	3.60
Molholm Hansen 2022	349	60143	1575	243592	0.90 (0.80, 1.01)	4.35
Molina-Merino 2021	52	9478	331	51484	0.85 (0.64, 1.14)	3.51
Muin 2021	68	12473	397	66176	0.91 (0.70, 1.18)	3.70
Oakley 2022	313	31839	1914	202678	1.04 (0.92, 1.17)	4.34
Rusconi 2023	3087	362129	10900	1117172	0.87 (0.84, 0.91)	4.54
Shah 2021	1045	126740	22617	2324866	0.85 (0.80, 0.90)	4.50
Stumpfe 2022	115	16015	1312	145018	0.80 (0.66, 0.96)	4.04
Wood 2021	62	4712	46	4644	1.32 (0.91, 1.93)	3.02
Overall, DL (I^2 = 94.8%, $p < 0.000$)	9919	1105446	66687	8577553	0.93 (0.83, 1.04)	100.00

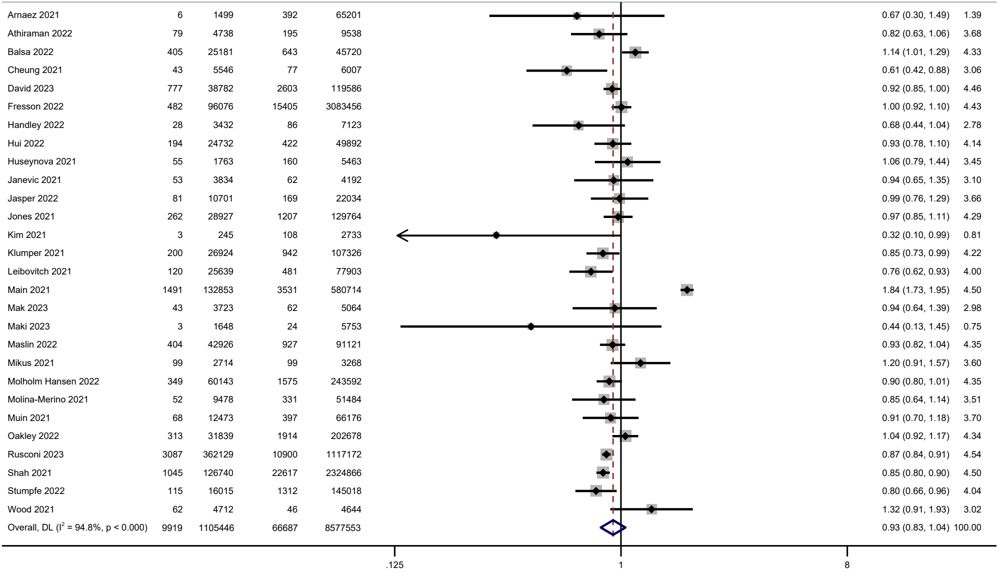

Supplementary Figure 9: Panel C

First Author Last Name & Year	Lockdown Events	Lockdown Livebirths	Pre-Lockdown Events	Pre-Lockdown Livebirths	Risk Ratio (95% CI)	% Weight
Arnaez 2021	79	1499	3760	65201	0.92 (0.74, 1.14)	1.24
Athiraman 2022	228	4738	506	9538	0.91 (0.78, 1.06)	2.22
Balsa 2022	2075	25181	3814	45720	0.99 (0.94, 1.04)	7.00
Cheung 2021	388	5546	550	6007	0.78 (0.69, 0.88)	2.97
Einarsdottir 2021	34	1057	194	3954	0.67 (0.47, 0.95)	0.50
Garabedian 2021	173	3885	184	4147	1.00 (0.82, 1.23)	1.39
Huseynova 2021	188	1763	787	5463	0.77 (0.66, 0.89)	2.26
Janevic 2021	277	3834	303	4192	1.00 (0.85, 1.17)	2.12
Jasper 2022	703	10701	1597	22034	0.91 (0.84, 0.99)	4.69
Jones 2021	1907	28927	8902	129764	0.96 (0.92, 1.01)	7.26
Lau 2023	22744	592392	74518	1808414	0.93 (0.92, 0.95)	9.37
Leibovitch 2021	1049	25639	3405	77903	0.94 (0.88, 1.00)	5.81
Main 2021	8352	132853	36521	580714	1.00 (0.98, 1.02)	8.97
Mak 2023	175	3723	232	5064	1.02 (0.85, 1.24)	1.54
Maki 2023	48	1648	199	5753	0.85 (0.62, 1.15)	0.65
Maslin 2022	1324	42926	2974	91121	0.95 (0.89, 1.01)	6.09
Molholm Hansen 2022	3003	60143	12564	243592	0.97 (0.93, 1.01)	7.93
Molina-Merino 2021	586	9478	3774	51484	0.85 (0.78, 0.93)	4.77
Muin 2021	608	12473	3375	66176	0.96 (0.88, 1.04)	4.78
Rusconi 2023	22463	362129	75047	1117172	0.93 (0.91, 0.94)	9.37
Shah 2021	8297	126740	149133	2324866	1.02 (1.00, 1.04)	9.05
Overall, DL (I^2 = 81.7%, $p < 0.000$)	74701	145309	382339	6668344	0.94 (0.92, 0.97)	100.00

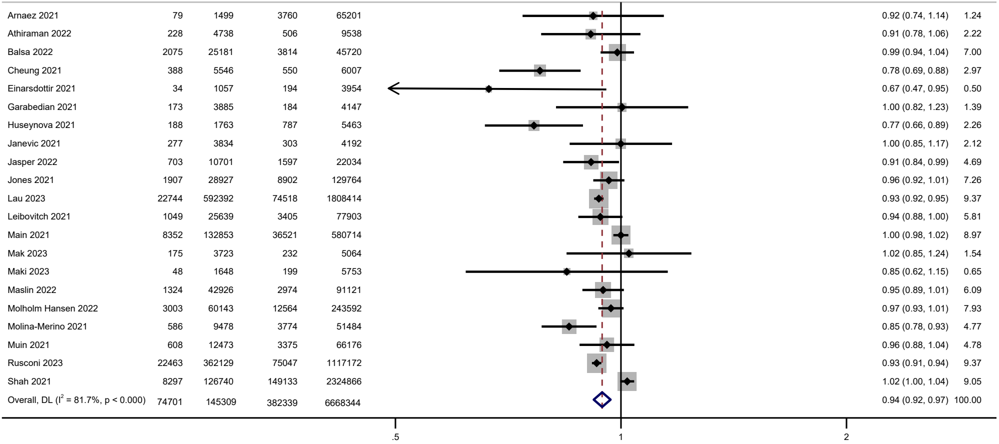

Supplementary Figure 10: Panel A.

First Author Last Name & Year	Lockdown Events	Lockdown Livebirths	Pre-Lockdown Events	Pre-Lockdown Livebirths	Risk Ratio (95% CI)	% Weight
Afonina 2021	64	858	67	968	1.07 (0.77, 1.49)	1.57
Alshaikh 2021	29	4334	204	24018	0.79 (0.54, 1.16)	1.16
Amadori 2021	13	256	13	293	1.14 (0.54, 2.41)	0.32
Athar 2022	133	4336	99	3154	0.98 (0.76, 1.26)	2.52
Gurol-Urganci 2022	7210	123966	28514	489054	1.00 (0.97, 1.02)	25.49
Handley 2022	16886	252155	52325	742113	0.95 (0.94, 0.97)	26.81
Maki 2023	86	1648	419	5753	0.73 (0.58, 0.92)	3.16
Molholm Hansen 2022	315	60143	1537	243592	0.83 (0.74, 0.94)	8.61
Rolnik 2021	322	3187	336	3229	0.97 (0.84, 1.13)	6.57
Simpson 2021	3826	67400	19225	346834	1.02 (0.99, 1.06)	23.80
Overall, DL (I^2 = 72.3%, p < 0.000)	28884	518383	102739	1859008	0.96 (0.92, 1.01)	100.00

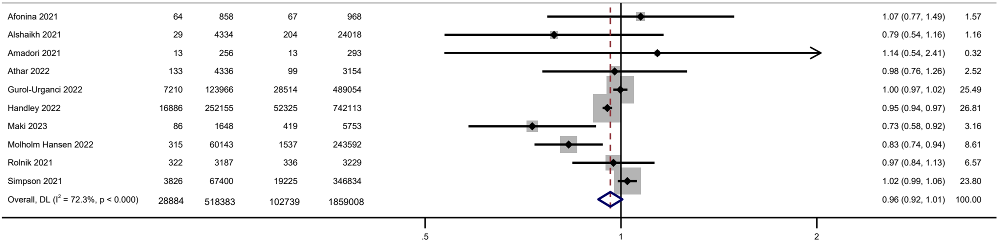

Supplementary Figure 10: Panel B

First Author Last Name & Year	Lockdown Events	Lockdown Livebirths	Pre-Lockdown Events	Pre-Lockdown Livebirths	Risk Ratio (95% CI)	% Weight
Alshaikh 2021	39	4334	348	24018	0.62 (0.45, 0.87)	7.58
Arnaez 2021	17	1499	653	65201	1.13 (0.70, 1.83)	4.60
Balsa 2022	380	25181	597	45720	1.15 (1.02, 1.31)	14.97
Garabedian 2021	127	3809	102	4071	1.32 (1.02, 1.71)	9.81
Gemmill 2021	20740	1516206	276839	19906408	0.98 (0.97, 1.00)	18.05
Jones 2021	201	28927	988	129764	0.91 (0.79, 1.06)	14.00
Kim 2021	3	245	100	2733	0.34 (0.11, 1.07)	1.02
Kirchengast 2021	6	669	274	29084	0.95 (0.43, 2.13)	1.95
Mak 2023	41	3723	56	5064	1.00 (0.67, 1.49)	5.93
Maki 2023	7	1648	46	5753	0.53 (0.24, 1.18)	2.00
Philip 2020	3	1381	49	5663	0.25 (0.08, 0.81)	0.99
Rolnik 2021	48	3187	79	3229	0.62 (0.44, 0.89)	6.92
Stumpfe 2022	110	16015	1190	145018	0.84 (0.69, 1.02)	12.19
Overall, DL (I^2 = 69.5%, p < 0.000)	21722	1606824	281321	20371706	0.91 (0.81, 1.03)	100.00

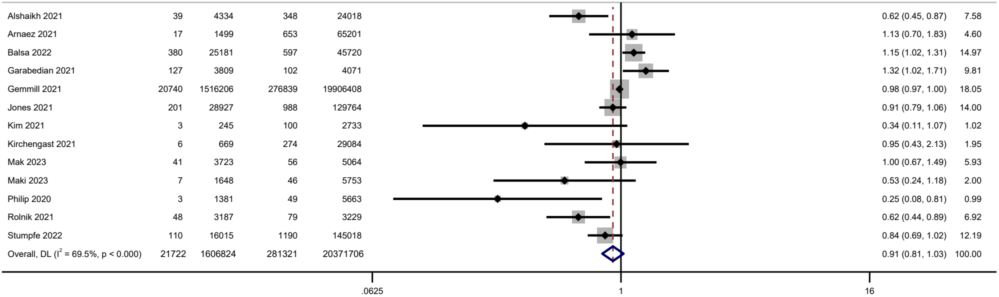

Supplementary Figure 10: Panel C

First Author Last Name & Year	Lockdown Events	Lockdown Livebirths	Pre-Lockdown Events	Pre-Lockdown Livebirths	Risk Ratio (95% CI)	% Weight
Alshaikh 2021	10	4334	125	24018	0.44 (0.23, 0.85)	12.13
Arnaez 2021	10	1499	224	65201	1.94 (1.03, 3.64)	12.39
Jeyamurugan 2023	2	360	11	427	0.22 (0.05, 0.99)	3.50
Jones 2021	166	28927	766	129764	0.97 (0.82, 1.15)	25.20
Kim 2021	2	245	54	2733	0.42 (0.10, 1.70)	3.92
Kirchengast 2021	2	669	130	29084	0.67 (0.17, 2.70)	3.97
Mak 2023	14	3723	22	5064	0.87 (0.44, 1.69)	11.61
Maki 2023	3	1648	11	5753	0.95 (0.27, 3.41)	4.61
Philip 2020	0	1381	22	5663	0.09 (0.01, 1.51)	1.10
Stumpfe 2022	47	16015	548	145018	0.78 (0.58, 1.05)	21.56
Overall, DL (I^2 = 53.4%, p = 0.023)	256	58801	1913	412725	0.80 (0.59, 1.08)	100.00

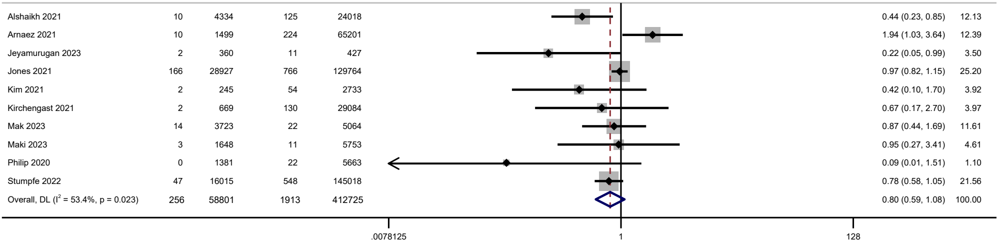

Supplementary Figure 10: Panel D

Subgroup and First Author Last Name & Year	Lockdown Events	Lockdown Livebirths	Pre-Lockdown Events	Pre-Lockdown Livebirths	Risk Ratio (95% CI)	% Weight
North America						
Alshaikh 2021	39	4334	348	24018	0.62 (0.45, 0.87)	1.52
Gemmill 2021	124574	1516206	1636908	1.99e+07	1.00 (0.99, 1.00)	16.03
Subgroup, DL (I^2 = 87.2%, p = 0.005)					0.81 (0.52, 1.29)	17.55
North America Total:	124613	1520540	1639256	19930426		
Europe						
Amadori 2021	16	256	19	293	0.97 (0.51, 1.84)	0.43
Arnaez 2021	17	1499	653	65201	1.13 (0.70, 1.83)	0.76
Esposito 2023	824	13435	1017	14902	0.90 (0.83, 0.99)	9.46
Garabedian 2021	464	3809	460	4071	1.07 (0.95, 1.21)	6.95
Jones 2021	1875	28927	8218	129764	1.02 (0.97, 1.07)	13.30
Kirchengast 2021	26	669	1539	29084	0.74 (0.51, 1.09)	1.17
Llorca 2021	34	620	83	969	0.66 (0.45, 0.97)	1.13
Philip 2020	3	1381	49	5663	0.25 (0.08, 0.81)	0.13
Stumpfe 2022	110	16015	1190	145018	0.84 (0.69, 1.02)	3.70
Subgroup, DL (I^2 = 63.5%, p = 0.005)					0.93 (0.84, 1.03)	37.02
Europe Total:	3369	66611	13228	394965		
Middle East						
Benyamini Raischer 2023	113	2442	136	2323	0.80 (0.63, 1.02)	2.58
Subgroup, DL (I^2 = 0.0%, p = .)					0.80 (0.63, 1.02)	2.58
Asia						
Hwang 2022	10242	255024	129807	3481423	1.07 (1.05, 1.10)	15.54
Mak 2023	324	3723	375	5064	1.16 (1.01, 1.34)	5.73
Maki 2023	107	1650	454	5762	0.83 (0.68, 1.02)	3.44
Okawa 2022	18	187	18	185	0.99 (0.53, 1.85)	0.45
Subgroup, DL (I^2 = 57.9%, p = 0.068)					1.04 (0.93, 1.17)	25.17
Asia Total:	10691	260584	130654	3492434		
Australia						
Rolnik 2021	281	3187	328	3229	0.88 (0.75, 1.02)	5.28
Subgroup, DL (I^2 = 0.0%, p = .)					0.88 (0.75, 1.02)	5.28
South America						
Balsa 2022	1645	25181	3109	45720	0.96 (0.91, 1.02)	12.41
Subgroup, DL (I^2 = 0.0%, p = .)					0.96 (0.91, 1.02)	12.41
Heterogeneity between groups: p = 0.292						
Overall, DL (I^2 = 81.9%, p = 0.000)					0.97 (0.93, 1.01)	100.00

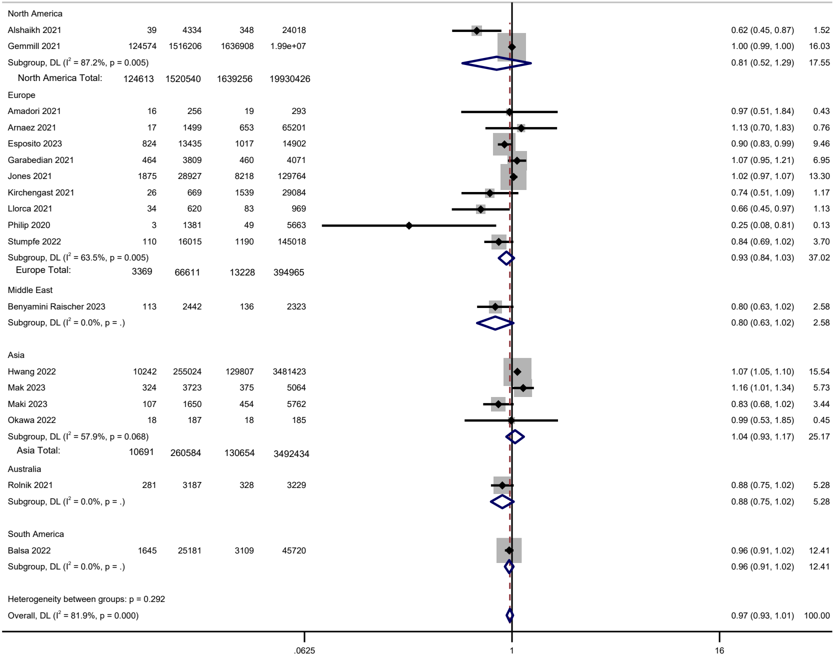

Supplementary Figure 11

Subgroup and First Author Last Name & Year	Lockdown Events	Lockdown Livebirths	Pre-Lockdown Events	Pre-Lockdown Livebirths	Risk Ratio (95% CI)	% Weight
North America						
Bajaj 2022	5903	77983	19378	250896	0.98 (0.95, 1.01)	7.99
Debolt 2022	124	1078	189	1910	1.15 (0.92, 1.42)	2.99
Jeyamurugan 2023	160	360	224	427	0.89 (0.76, 1.06)	4.01
Racine 2022	88	1210	57	1058	1.33 (0.96, 1.83)	1.66
Richter 2022	203	2558	1180	18849	1.25 (1.08, 1.44)	4.63
Riley 2021	2098	20704	8853	78718	0.91 (0.87, 0.95)	7.64
Roberts 2022	12495	93711	94275	702419	0.99 (0.98, 1.01)	8.14
Shukla 2023	7166	73448	20014	234356	1.13 (1.10, 1.16)	8.03
Subgroup, DL (I^2 = 93.8%, p < 0.000)					1.03 (0.97, 1.10)	45.10
North America Total:	28237	271052	144170	1288633		
Europe						
Ameh 2021	95	1461	83	1553	1.20 (0.90, 1.60)	2.01
Athiraman 2022	564	4738	1180	9538	0.97 (0.88, 1.06)	6.15
Khalil 2020	106	1702	103	1677	1.01 (0.78, 1.32)	2.28
Maslin 2022	3815	42926	8419	91121	0.97 (0.93, 1.00)	7.83
Quibel 2022	495	3885	478	4147	1.09 (0.97, 1.23)	5.37
Wagner 2021	1395	33198	7271	188225	1.08 (1.03, 1.15)	7.36
Subgroup, DL (I^2 = 68.5%, p = 0.007)					1.03 (0.96, 1.10)	30.99
Europe Total:	6470	87908	17534	296261		
Middle East						
Athar 2022	218	4336	230	3154	0.70 (0.59, 0.84)	3.70
Herzberger 2021	85	2668	82	2701	1.05 (0.78, 1.41)	1.88
Justman 2020	49	610	59	742	1.01 (0.70, 1.45)	1.37
Meyer 2021	78	2572	1048	28396	0.83 (0.66, 1.04)	2.80
Mor 2021	57	1550	212	4559	0.80 (0.60, 1.06)	2.00
Subgroup, DL (I^2 = 39.5%, p = 0.158)					0.84 (0.72, 0.97)	11.76
Middle East Total:	487	11736	1631	39552		
Asia						
Hiiragi 2021	62	279	55	339	1.30 (0.93, 1.82)	1.59
Kim 2021	126	245	737	2733	1.60 (1.37, 1.87)	4.30
Okawa 2022	10	187	13	185	0.77 (0.35, 1.72)	0.33
Subgroup, DL (I^2 = 49.6%, p = 0.138)					1.38 (1.05, 1.83)	6.21
Asia Total:	198	711	805	3257		
Australia						
Hui 2022	551	24732	1113	49892	1.00 (0.90, 1.10)	5.94
Subgroup, DL (I^2 = 0.0%, p < 0.000)					1.00 (0.90, 1.10)	5.94
Heterogeneity between groups: p = 0.018						
Overall, DL (I^2 = 88.2%, p < 0.000)					1.03 (0.98, 1.08)	100.00

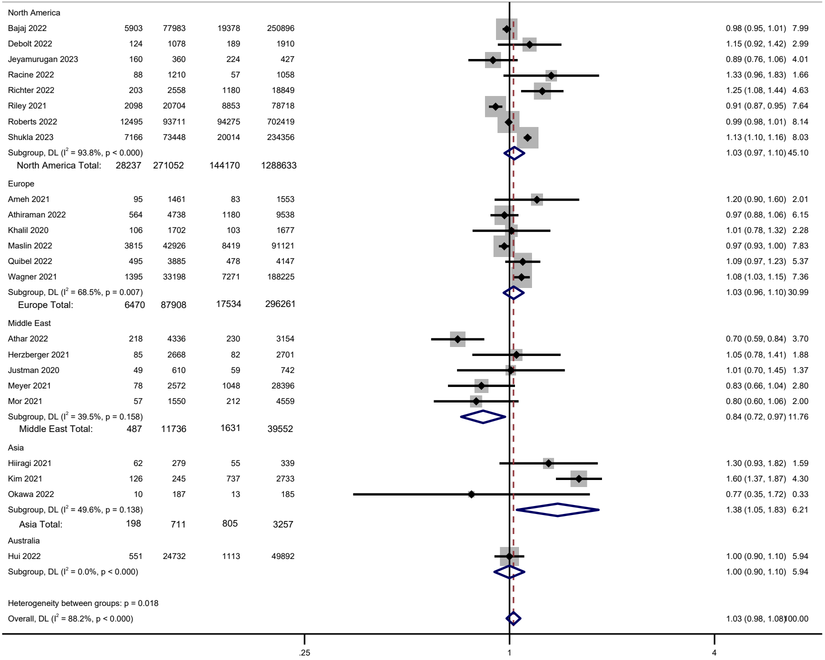

Supplementary Figure 12: Panel A

First Author Last Name & Year	Lockdown Events	Lockdown Total Births	Pre-Lockdown Events	Pre-Lockdown Total Births	Risk Ratio (95% CI)	% Weight
Amadori 2021	15	256	28	293	0.63 (0.35, 1.16)	0.41
Boehler-Tatman 2022	5	254	14	235	0.34 (0.13, 0.94)	0.15
Einarsdottir 2021	47	1060	255	3962	0.70 (0.52, 0.95)	1.56
Esposito 2023	1996	13476	2164	14952	1.02 (0.96, 1.08)	20.50
Gurol-Urganci 2022	17045	126093	64079	496293	1.04 (1.02, 1.06)	34.44
Hirsch 2021	147	2343	161	2665	1.04 (0.83, 1.29)	2.94
Jasper 2022	79	547	125	1095	1.23 (0.95, 1.60)	2.03
Jones 2021	231	29031	1110	130326	0.93 (0.81, 1.08)	6.27
Mor 2021	91	1556	255	4564	1.04 (0.83, 1.32)	2.59
Okawa 2022	20	187	21	185	0.95 (0.53, 1.69)	0.44
Rao 2022	185	1058	208	1384	1.14 (0.95, 1.37)	3.98
Rolnik 2021	444	3150	428	3175	1.04 (0.92, 1.18)	7.68
Sinnott 2021	13	1019	9	890	1.26 (0.54, 2.93)	0.21
Speyer 2022	1288	7342	1367	8323	1.06 (0.99, 1.14)	16.61
Zanardo 2020	9	91	8	101	1.23 (0.49, 3.06)	0.18
Overall, DL (I^2 = 27.9%, p = 0.149)	21615	187463	70232	668443	1.03 (0.99, 1.07)	100.00

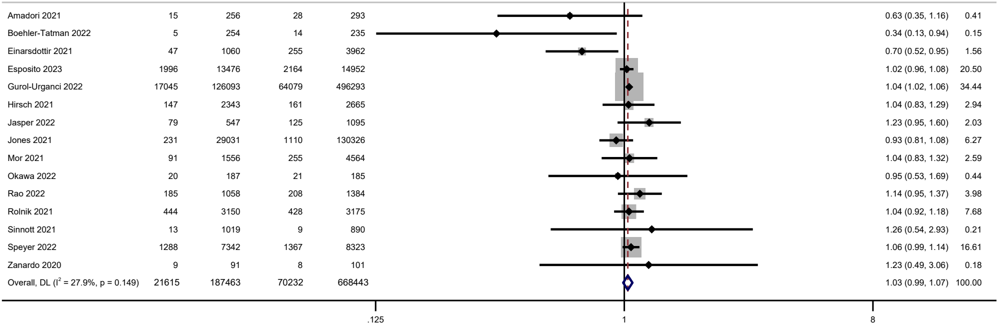

Supplementary Figure 12: Panel B

First Author Last Name & Year	Lockdown Events	Lockdown Total Births	Pre-Lockdown Events	Pre-Lockdown Total Births	Risk Ratio (95% CI)	% Weight
Amadori 2021	36	256	28	293	1.41 (0.89, 2.26)	0.37
Einarsdottir 2021	98	1060	388	3962	0.95 (0.77, 1.17)	1.72
Esposito 2023	1147	13476	1326	14952	0.96 (0.89, 1.04)	9.22
Gurol-Urganci 2022	21841	126093	84156	496293	1.02 (1.00, 1.03)	24.27
Hirsch 2021	149	2343	154	2665	1.09 (0.88, 1.36)	1.62
Jasper 2022	68	547	193	1095	0.74 (0.57, 0.96)	1.17
Jones 2021	3090	29031	14761	130326	0.95 (0.91, 0.98)	18.07
Justman 2020	71	610	105	742	0.84 (0.63, 1.12)	0.99
Lau 2023	7812	594728	23534	1815118	1.01 (0.99, 1.04)	21.39
Mor 2021	54	1556	156	4564	1.01 (0.75, 1.38)	0.86
Okawa 2022	16	187	20	185	0.81 (0.43, 1.51)	0.21
Racine 2022	217	1210	214	1058	0.90 (0.76, 1.07)	2.49
Rolnik 2021	495	3150	457	3175	1.08 (0.96, 1.22)	4.79
Sinnott 2021	195	1019	176	890	0.97 (0.81, 1.17)	2.18
Speyer 2022	1363	7342	1488	8323	1.03 (0.96, 1.10)	10.62
Zanardo 2020	3	91	4	101	0.84 (0.19, 3.65)	0.04
Overall, DL (I^2 = 48.6%, p = 0.015)	36655	782699	127160	2483742	0.99 (0.97, 1.02)	100.00

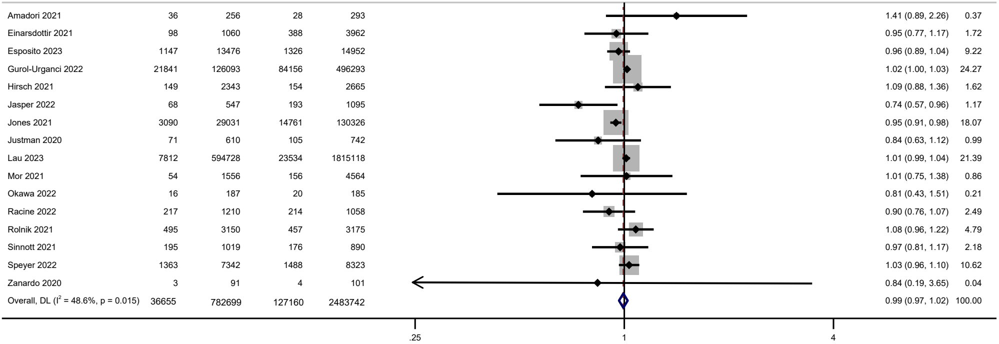

Supplementary Figure 12: Panel C

Subgroup and First Author Last Name & Year	Lockdown Events	Lockdown Total Births	Pre-Lockdown Events	Pre-Lockdown Total Births	Risk Ratio (95% CI)	% Weight
North America						
Gemmill 2021	482516	1516206	6344801	19906408	1.00 (1.00, 1.00)	10.93
Roberts 2022	28641	94126	200055	705767	1.06 (1.04, 1.07)	10.50
Subgroup, DL (I^2 = 99.0%, p < 0.000)					1.03 (0.97, 1.08)	21.43
North America Total:	511057	1610332	6544857	20612175		
Europe						
Deli 2021	261	748	209	517	0.90 (0.77, 1.05)	1.10
Delius 2023	57	333	110	704	1.08 (0.80, 1.45)	0.32
Einarsdottir 2021	145	1060	643	3962	0.86 (0.73, 1.02)	0.94
Esposito 2023	3143	13476	3490	14952	1.00 (0.96, 1.04)	6.42
Fresson 2022	18963	96880	607325	3108192	1.00 (0.99, 1.01)	10.29
Jones 2021	6224	29031	28489	130326	0.98 (0.96, 1.01)	8.90
Llorca 2021	110	620	236	969	0.77 (0.63, 0.95)	0.64
Mikus 2021	881	2732	1024	3277	1.02 (0.95, 1.11)	3.31
Speyer 2022	2651	7342	2855	8323	1.04 (0.99, 1.09)	6.17
Wagner 2021	9002	33198	51516	188225	0.99 (0.97, 1.01)	9.55
Zanardo 2020	12	91	12	101	1.10 (0.52, 2.33)	0.05
Subgroup, DL (I^2 = 38.3%, p = 0.094)					1.00 (0.98, 1.01)	47.69
Europe Total:	41339	185511	695909	3459547		
Middle East						
Benyamini Raischer 2023	392	2442	400	2323	0.94 (0.83, 1.07)	1.51
Herzberger 2021	407	2668	435	2701	0.95 (0.84, 1.08)	1.58
Justman 2020	147	610	164	742	1.07 (0.88, 1.31)	0.68
Meyer 2020	491	1666	463	1654	1.04 (0.93, 1.16)	1.92
Meyer 2021	661	2594	6990	28686	1.04 (0.97, 1.11)	3.77
Mor 2021	273	1556	809	4564	0.99 (0.87, 1.13)	1.56
Subgroup, DL (I^2 = 0.0%, p = 0.679)					1.01 (0.97, 1.06)	11.04
Middle East Total:	2371	11536	9261	40670		
Asia						
Kim 2021	97	246	906	2765	1.15 (0.96, 1.37)	0.85
Mak 2023	1143	3723	1305	5064	1.15 (1.07, 1.23)	3.86
Maki 2023	289	1650	1040	5762	0.97 (0.86, 1.10)	1.71
Suzuki 2020	40	138	43	148	1.00 (0.68, 1.46)	0.20
Subgroup, DL (I^2 = 47.1%, p = 0.129)					1.08 (0.98, 1.19)	6.61
Asia Total:	1569	5757	3293	13739		
Australia						
Rolnik 2021	1061	3150	989	3175	1.06 (0.98, 1.14)	3.50
Subgroup, DL (I^2 = 0.0%, p < 0.000)					1.06 (0.98, 1.14)	3.50
South America						
Balsa 2022	12020	25307	21213	45950	1.02 (1.00, 1.04)	9.72
Subgroup, DL (I^2 = 0.0%, p < 0.000)					1.02 (1.00, 1.04)	9.72
Heterogeneity between groups: p = 0.189						
Overall, DL (I^2 = 82.8%, p < 0.000)					1.01 (1.00, 1.03)	100.00

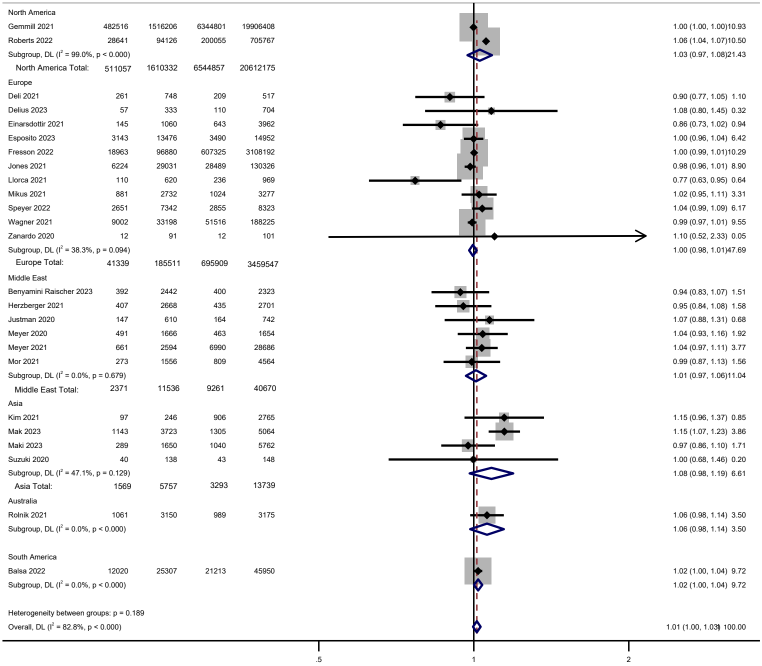

Supplementary Figure 13

Subgroup and First Author Last Name & Year	Lockdown Events	Lockdown Total Births	Pre-Lockdown Events	Pre-Lockdown Total Births	Risk Ratio (95% CI)	% Weight
North America						
Debolt 2022	14	1078	19	1910	1.30 (0.66, 2.59)	6.11
Facco 2021	94	2417	88	2368	1.04 (0.79, 1.39)	13.09
Gulerson 2022	46	4038	50	4168	0.95 (0.64, 1.41)	10.68
Panzer 2022	22	643	25	715	0.98 (0.56, 1.72)	7.74
Racine 2022	16	1210	17	1058	0.83 (0.42, 1.63)	6.21
Riley 2021	354	20704	1178	78718	1.14 (1.01, 1.28)	16.32
Simoene 2022	7665	614093	8224	663620	1.01 (0.98, 1.04)	17.13
Subgroup, DL (I^2 = 0.0%, p = 0.543)					1.02 (0.99, 1.05)	77.27
North America Total:	8211	644183	9601	752557		
Europe						
Gurol-Urganci 2022	3161	122851	15888	418393	0.69 (0.66, 0.71)	17.10
Subgroup, DL (I^2 = 0.0%, p < 0.000)					0.69 (0.66, 0.71)	17.10
Middle East						
Hirsch 2021	16	2343	13	2665	1.40 (0.67, 2.90)	5.63
Subgroup, DL (I^2 = 0.0%, p < 0.000)					1.40 (0.67, 2.90)	5.63
Heterogeneity between groups: p = 0.000						
Overall, DL (I^2 = 97.0%, p < 0.000)					0.98 (0.79, 1.21)	100.00

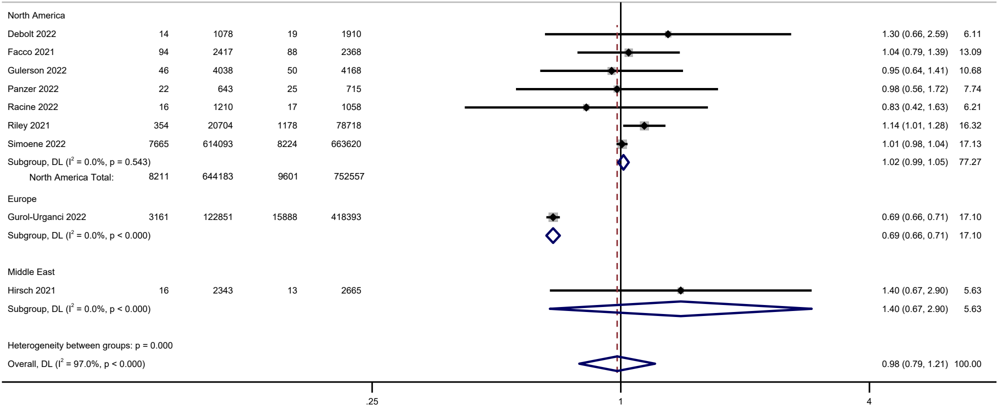

Supplementary Figure 14: Panel A

Subgroup and First Author Last Name & Year	Lockdown: Screened positive for depression in pregnancy	Lockdown: Pregnant Women	Pre-Lockdown: Screened positive for depression in pregnancy	Pre-Lockdown: Pregnant Women	Risk Ratio (95% CI)	% Weight
North America						
Berthelot 2020	137	1258	30	496	1.72 (1.18, 2.52)	11.64
Gustafsson 2021	175	247	23	155	3.21 (2.16, 4.78)	11.40
Lantigua-Martinez 2022	2	240	12	626	0.44 (0.10, 1.95)	2.57
McKee 2023	137	785	208	1570	1.27 (1.04, 1.55)	14.20
Zhang 2022	6	110	16	263	0.90 (0.36, 2.25)	5.36
Subgroup, DL (I^2 = 80.9%, p < 0.000)					1.51 (0.94, 2.44)	45.17
North America Total:	457	2640	289	3110		
Europe						
De Chiara 2022	18	72	8	68	1.90 (0.88, 4.12)	6.56
Kuipers 2022	119	341	60	200	1.12 (0.86, 1.47)	13.29
Vacaru 2021	32	307	76	1337	1.76 (1.18, 2.61)	11.43
Zilver 2021	145	1102	57	364	0.86 (0.65, 1.14)	13.07
Subgroup, DL (I^2 = 70.1%, p = 0.018)					1.24 (0.88, 1.75)	44.36
Europe Total:	314	1822	201	1969		
Australia						
Clifton 2022	27	156	37	243	1.12 (0.71, 1.77)	10.47
Subgroup, DL (I^2 = 0.0%, p < 0.000)					1.12 (0.71, 1.77)	10.47
Heterogeneity between groups: p = 0.662						
Overall, DL (I^2 = 75.9%, p < 0.000)					1.37 (1.06, 1.78)	100.00

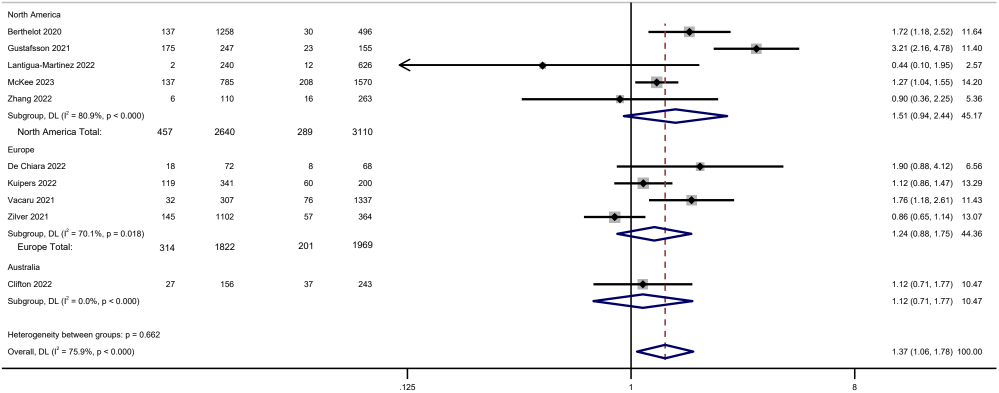

Supplementary Figure 14: Panel B

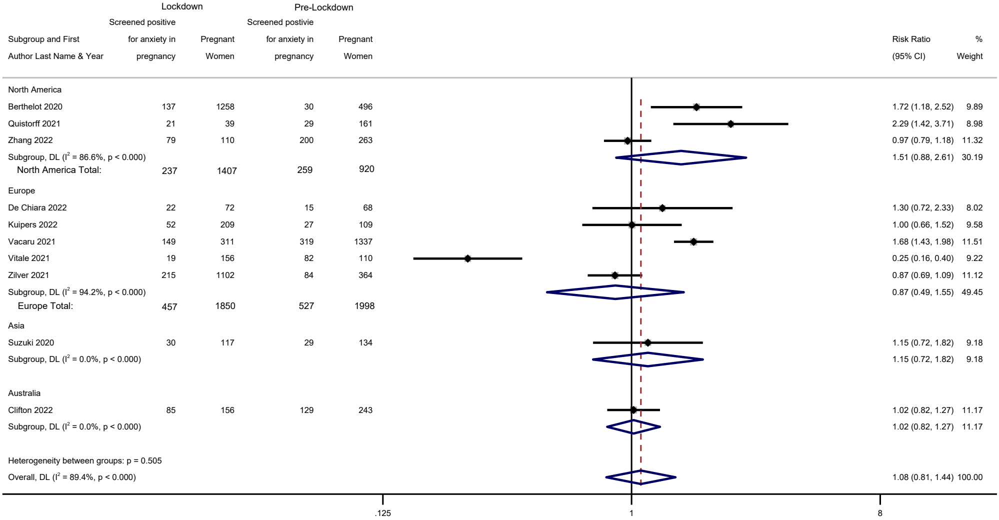

Supplementary Figure 15: Panel A.
Funnel plot of studies reporting spontaneous PTB, with pseudo 95% confidence limits

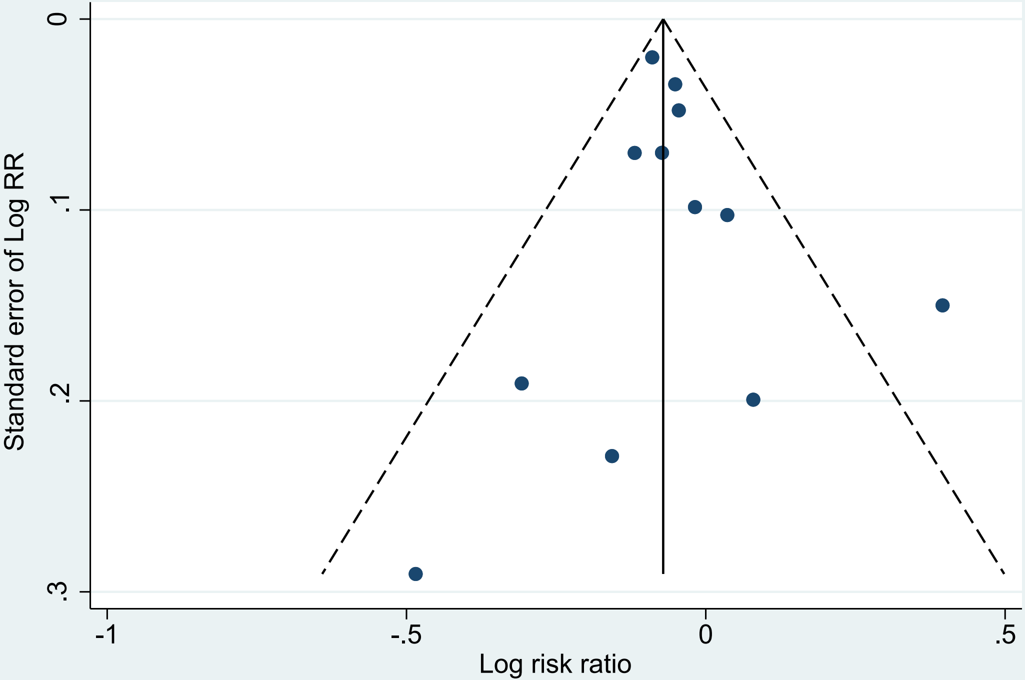

Label: Egger's test p-value=0.533

Supplementary Figure 15: Panel B.
Funnel plot of studies reporting iatrogenic PTB, with pseudo 95% confidence limits

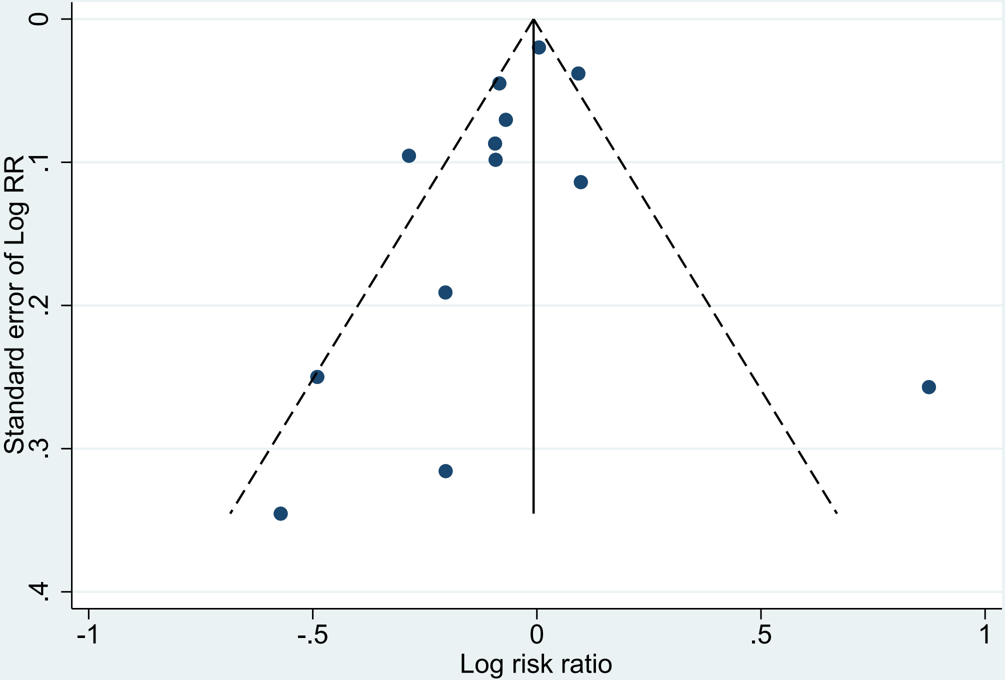

Label: Egger's test p-value=0.304

Supplementary Figure 15: Panel C.
Funnel plot of studies reporting extreme PTB, with pseudo 95% confidence limits

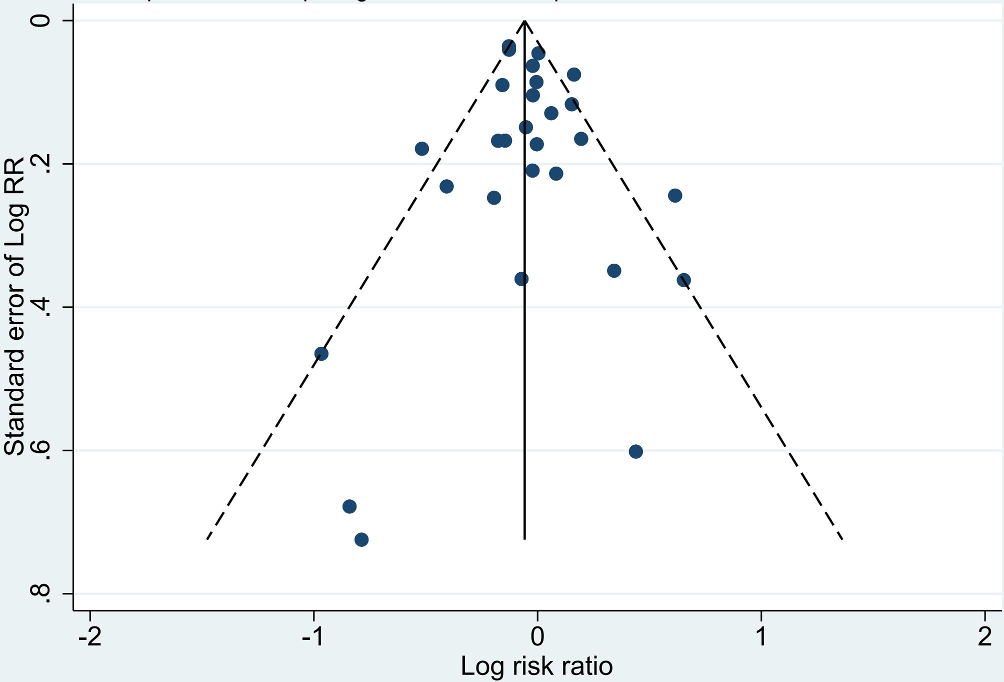

Label: Egger's test p-value=0.535

Supplementary Figure 15: Panel D.
Funnel plot of studies reporting very PTB, with pseudo 95% confidence limits

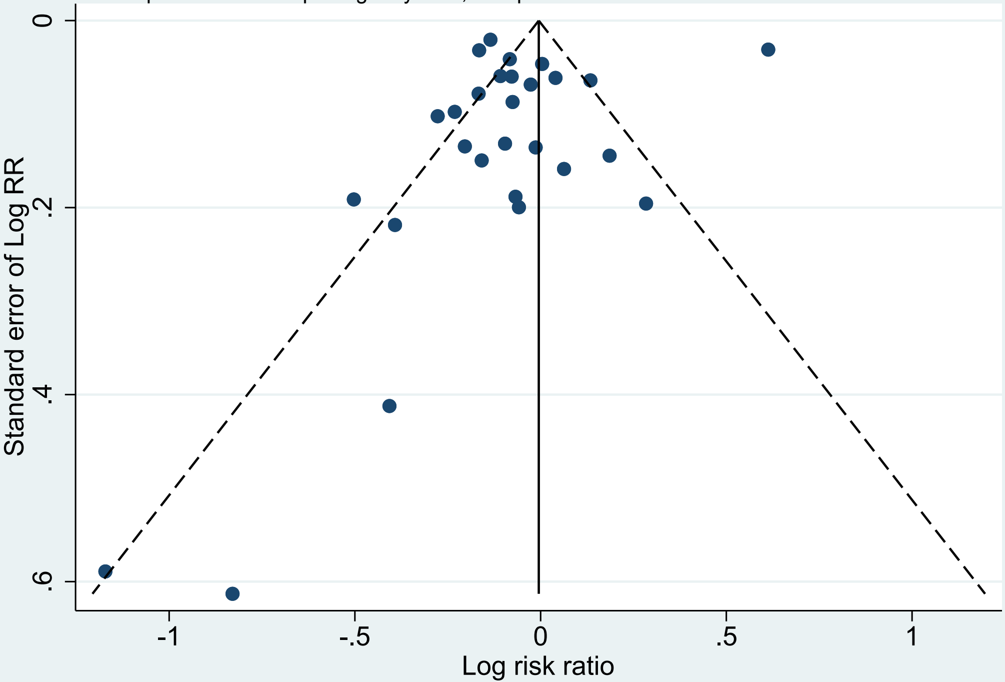

Label: Egger's test p-value=0.481

Supplementary Figure 15: Panel E.
Funnel plot of studies reporting moderate to late PTB, with pseudo 95% confidence limits

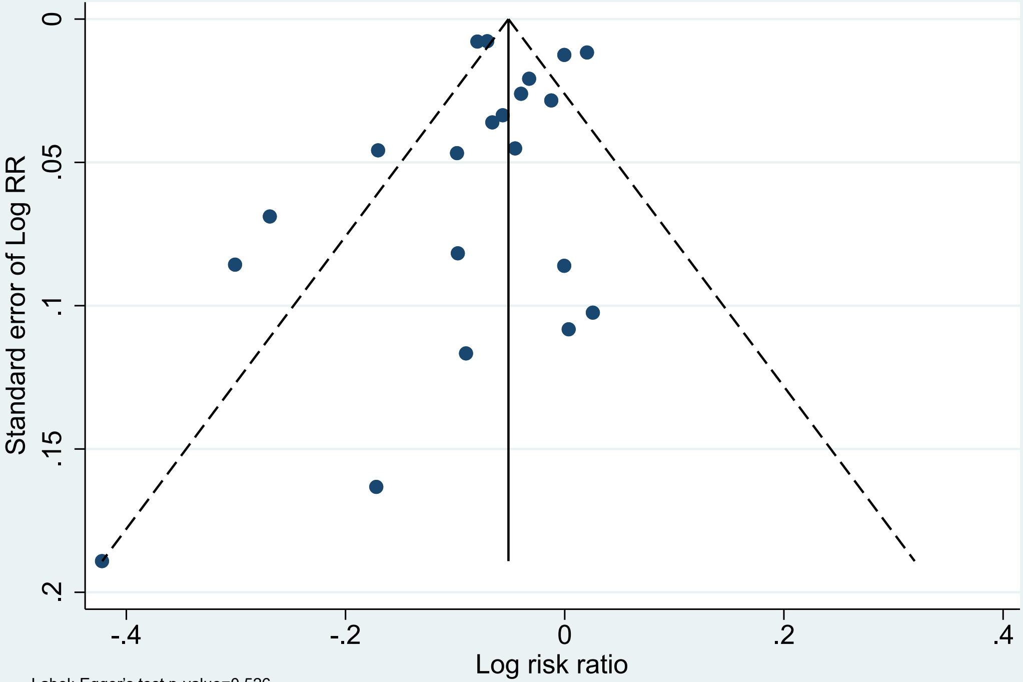

Label: Egger's test p-value=0.526

Supplementary Figure 15: Panel F.
Funnel plot of studies reporting VLBW, with pseudo 95% confidence limits

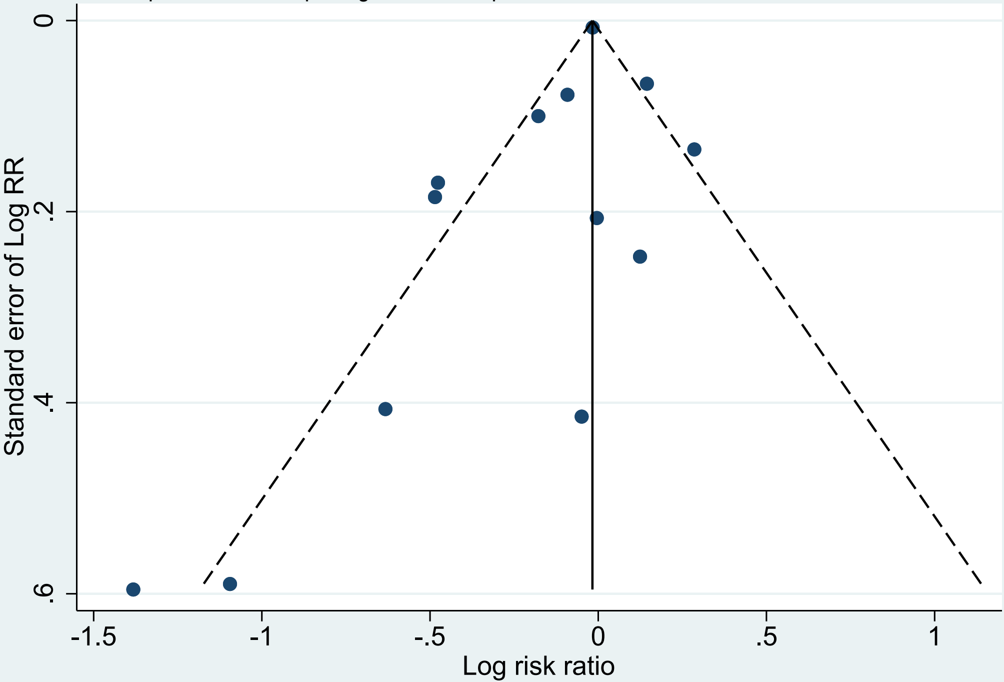

Label: Egger's test p-value=0.184

Supplementary Figure 15: Panel G.
Funnel plot of studies reporting planned caesarean section, with pseudo 95% confidence limits

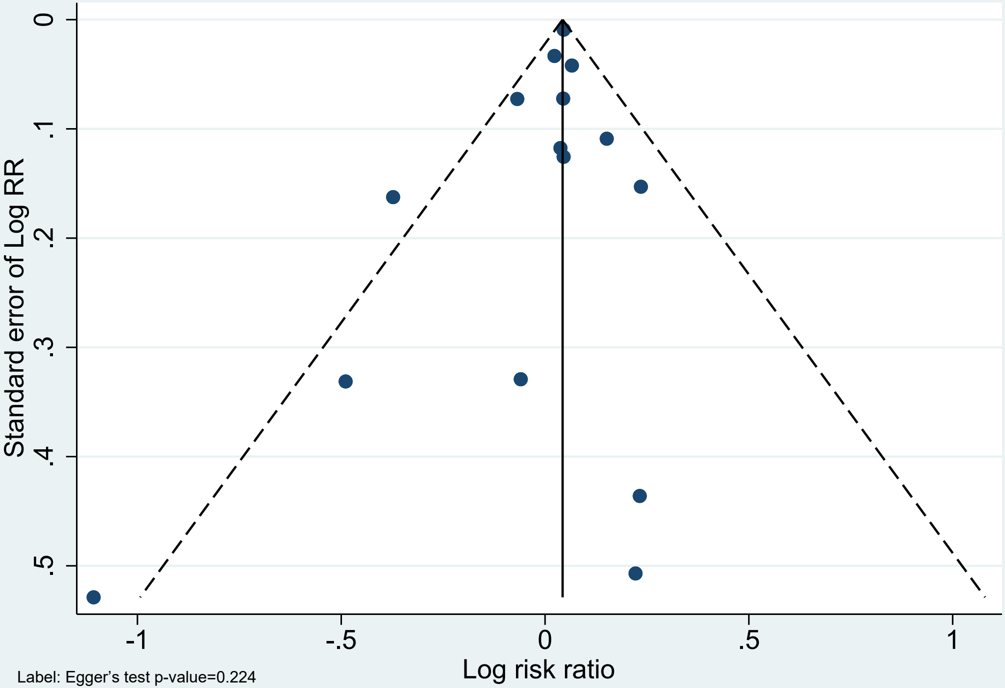

Supplementary Figure 15: Panel H.
Funnel plot of studies reporting emergency caesarean section, with pseudo 95% confidence limits

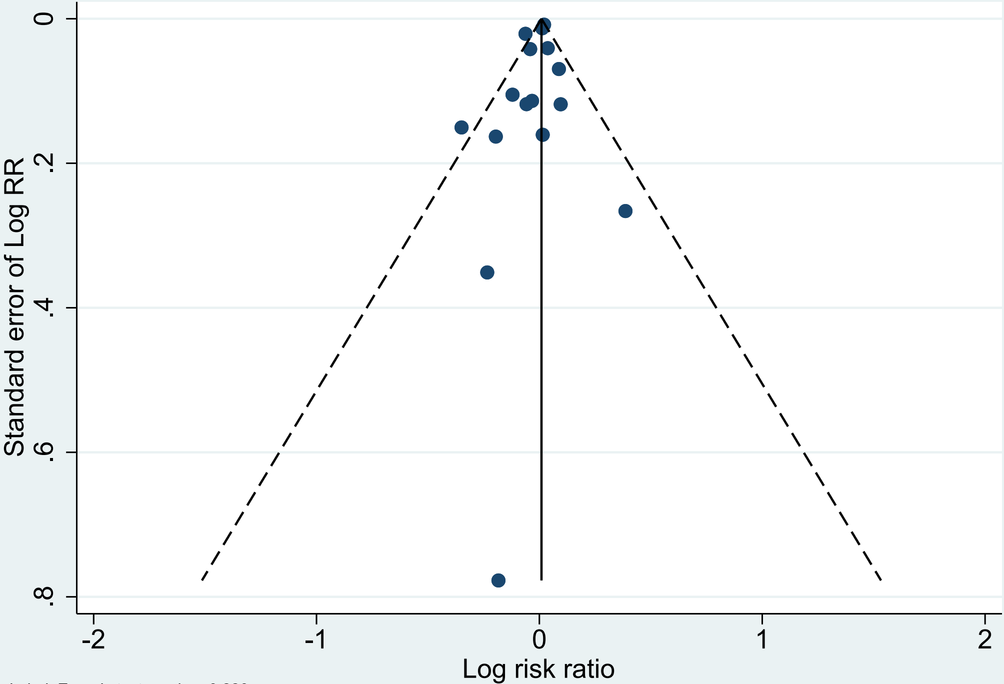

Label: Egger's test p-value=0.220
